# CLDN6 triggers NRF2-mediated ferroptosis through recruiting DLG1/PBK complex in breast cancer

**DOI:** 10.1038/s41419-025-07448-9

**Published:** 2025-02-21

**Authors:** Da Qi, Yan Lu, Huinan Qu, Yuan Dong, Qiu Jin, Minghao Sun, Chengshi Quan

**Affiliations:** 1 The Key Laboratory of Pathobiology, Ministry of Education, College of Basic Medical Sciences, Jilin University, 126 Xinmin Avenue, Changchun, 130021 China; 2 Department of Anatomy, College of Basic Medical Sciences, Jilin University, 126 Xinmin Avenue, Changchun, 130021 China; 3 Department of Histology and Embryology, College of Basic Medical Sciences, Jilin University, 126 Xinmin Avenue, Changchun, 130021 China

**Keywords:** Breast cancer, Cell death, Prognostic markers, Tumour biomarkers, Protein translocation

## Abstract

We previously identified CLDN6 as a pivotal tumor suppressor in breast cancer and unexpectedly discovered that overexpression of CLDN6 resulted in characteristic ultrastructural alterations of ferroptosis. However, the exact mechanism by which CLDN6 triggers ferroptosis is still elusive in breast cancer. Our study showed that CLDN6 was associated with ferroptosis in breast cancer patients. The integration of CLDN6 and ferroptosis demonstrated remarkable predictive prognostic performance. We observed that CLDN6 triggers NRF2-mediated ferroptosis in vitro and in vivo. Mechanistically, CLDN6 enhanced nuclear export of NRF2 by regulating the PBK-dependent AKT/GSK3β/FYN axis. Further CLDN6 recruited PBK to the cell membrane through the endosomal pathway and bound with the DLG1/PBK complex, thereby promoted the degradation of PBK by the UPS. This study elucidates the previously unrecognized mechanism of CLDN6 triggering NRF2-mediated ferroptosis through recruiting DLG1/PBK complex. This study provides a reliable biomarker for predicting prognosis and is anticipated to guide the selection of therapies targeting ferroptosis in breast cancer.

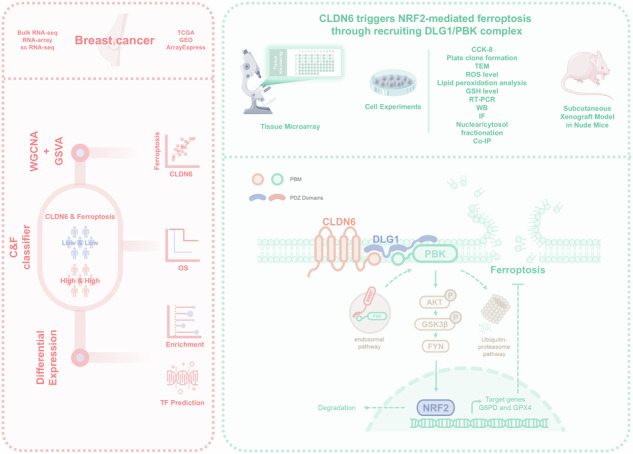

## Introduction

Ferroptosis as a unique form of regulated cell death (RCD) is driven by iron-dependent lethal lipid peroxides [1]. Recent investigations have corroborated that ferroptosis, as a potential prognostic predictor, possesses the capacity to impede the growth and progression of breast cancer [2, 3]. Ferroptosis is regulated by multiple factors, wherein NRF2 is a transcriptional coactivator that plays an important role in suppressing ferroptosis through its target genes, such as GPX4 and G6PD [4, 5]. At present, there are still broad prospects for the trigger mechanism of ferroptosis in breast cancer.

CLDN6 belongs to the family of tight junction proteins that regulate permeability and form barriers [6]. Prior to this study, our group had cloned the CLDN6 gene from mammary epithelial cells of COP rats for the first time and found that CLDN6 exerted tumor suppressive function while its expression was low in breast cancer [7–9]. We previously made the unexpected discovery that ultrastructural changes of ferroptosis occurred in breast cancer cells overexpressing CLDN6. Meanwhile, we found that CLDN6 was associated with multiple NRF2 target genes in the TCGA breast cancer cohort. It is possible therefore that CLDN6 inhibits ferroptosis through NRF2. Currently, a variety of molecules are responsible for regulating NRF2, but there remains no mechanistic explanation for the effect of CLDN6 on NRF2.

The C-terminus of CLDN6 contains PBM, which plays important functions such as signal transduction by binding to the proteins having the PDZ domain. DLG1, a scaffold protein, contains three PDZ domains, in which the PDZ2 domain mediates the formation of the DLG1/PBK complex [10–12]. PBK is a serine/threonine protein kinase, and the PBM possessed on it is a potential binding site for SNX27, a sorter that regulates the transport of cargo by endosomal pathway to the cell membrane [13, 14]. Reportedly, PBK leads to the activation of AKT [15]. It has been conclusively shown that the AKT/GSK3β/FYN axis regulates the export of NRF2 from the nucleus to the cytoplasm for degradation [16–18]. Currently, it has not been reported that CLDN6 regulated the AKT/GSK3β/FYN axis through PBK to promote NRF2 nuclear export.

Our study showed that the integration of CLDN6 and ferroptosis exhibited exceptional predictive performance. We observed that CLDN6 triggers NRF2-mediated ferroptosis in vitro and in vivo. In terms of mechanism, CLDN6 enhanced the nuclear export of NRF2 by regulating the PBK-dependent AKT/GSK3β/FYN axis. Further CLDN6 recruited PBK to the cell membrane through the endosomal pathway and bound with the DLG1/PBK complex, then promoted the degradation of PBK by the UPS. In conclusion, this study opens up new possibilities for CLDN6 integrated with ferroptosis to predict the prognosis and reveals novel insights into the mechanism by which CLDN6 activates the ferroptosis process in breast cancer.

## Material and methods

### Immunohistochemistry (IHC)

We purchased BrcSur2201 tissue microarray (TMA) containing 80 breast cancer and para-carcinoma paired samples (74 with complete prognostic follow-up information) from Hunan Aifang Biotechnology Co., LTD. Informed consent has been obtained from all patients. IHC assay was performed as previously described [9]. The antibodies are listed in Table. S1.

### Transmission electron microscopy (TEM)

Fixation in glutaraldehyde 4% and post-fixation in OsO_4_ 1% was performed on the samples. The samples were dehydrated by ethanol solutions, then embedded in Eponate 12 epoxy resin. Uranyl acetate and lead citrate were used to counterstain ultrathin sections. Observation and photography were conducted using transmission electron microscopy equipment (FEI Tecnai Spirit, USA).

### Detection of intracellular ROS

1 × 10^5^ cells were seeded in 48-well light-proof plates with 3 parallel wells per group in 500 µL media. Incubation was continued for 24 h at 37 °C. DCFH-DA (Meilune, China) was then loaded into the wells at 10 µM, a fluorescent probe for ROS. A fluorescence microscope (Olympus, Japan) was used for observation after further culture for 20 min in the dark.

### Lipid peroxidation analysis

1 × 10^4^ cells were incubated with 15 μM BODIPY-C11 (ABclonal, China) for 30 min at 37 °C. Cells were washed 3 times with PBS, harvested, and suspended in a serum-free medium observed by fluorescence microscope (Olympus, Japan). After washing 3 times with PBS, the cells were observed by fluorescence microscope (Olympus, Japan).

### Detection of GSH level

The measurement of GSH level was assessed using GSH Assay Kit (Solarbio, China). 5 × 10^6^ cells cultured in DMEM were collected and repeated freeze-thaw lysing in liquid nitrogen. Microplate readers (Thermo, Germany) was used to measure the sample at 420 nm. The results were normalized by the number of cells in each sample.

### Immunofluorescence (IF)

Cells were fixed with 4% paraformaldehyde and then incubated with 0.5% Triton X-100 (if desired) and 5% BSA. Incubated overnight with the first antibody and stained with DAPI following the secondary antibody, the cells were visualized under fluorescence microscope (Olympus, Japan) or confocal laser scanning microscope (Olympus, Japan). A list of the antibodies used in IF can be found in Table. S4.

### Co-immunoprecipitation (Co-IP) assay

In accordance with the previous description, the co-IP assay was performed [19]. An anti-CLDN6 (E2S5M, Cell Signaling Technology, USA) and anti-PBK (A19947, ABclonal, China) antibody was used for co-IP.

Details for bioinformatics analysis, cell culture, transfection, cell counting kit-8, plate clone formation, in vivo tumor xenograft model, RNA extraction and RT-PCR, western blot, nuclear/cytosol fractionation, molecular docking, medical illustrations, statistics analysis, ethics approval and consent to participate see Supplementary Methods.

## Results

### The prognostic significance of integrating CLDN6 and ferroptosis

**Fig. 1:**
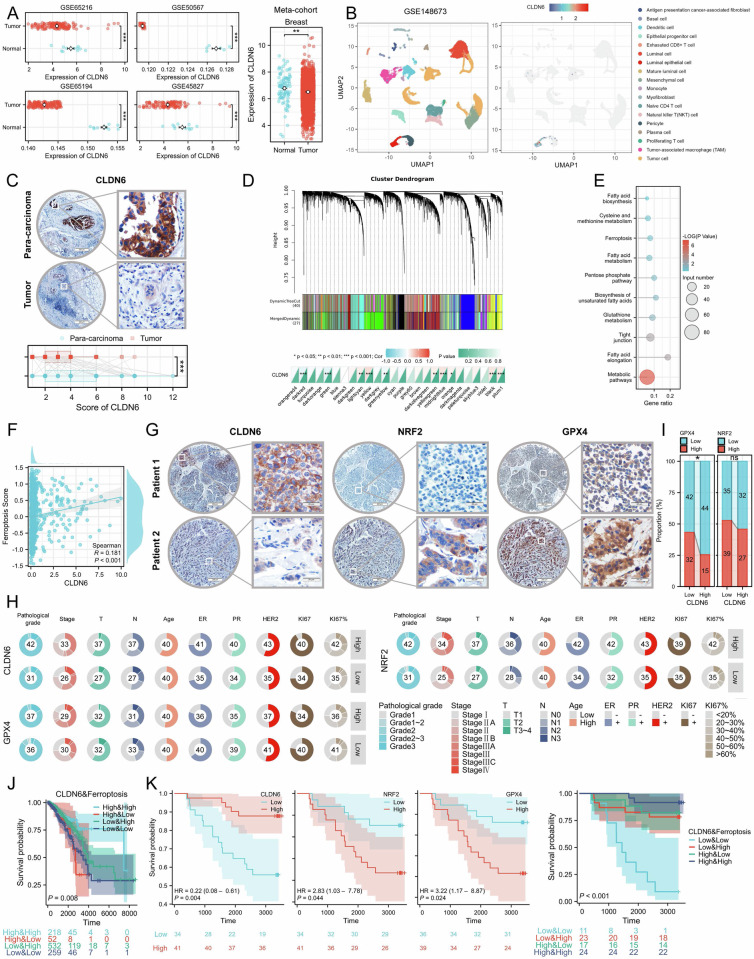
Prognostic significance of integrating CLDN6 with ferroptosis. **A** CLDN6 expression in four independent cohorts and one meta-cohort of breast cancer and normal tissue. **B** Feature plots display expression of CLDN6 across individual cells. **C** Representative IHC images of CLDN6 expression in para-carcinoma and breast cancer tissues. Scale bar, 200 µm (left), 20 µm (right). **D** The hierarchical gene dendrogram and module color of WGCNA, and heatmaps showed that CLDN6 co-expressed modules. **E** The KEGG analysis results of the genes co-expressed with CLDN6. **F** Correlation between CLDN6 and ferroptosis score in TCGA breast cancer cohort. **G** Representative IHC images of low and high CLDN6, NRF2, and GPX4 expression in breast cancer tissues. Scale bar, 200 µm (left), 20 µm (right). **H** The distribution of clinicopathologic features in low and high CLDN6, NRF2 and GPX4 groups. The total number of patients is shown in the pie diagrams. **I** The distribution of NRF2 and GPX4 in low and high CLDN6 groups. **J** Survival analysis showed differences in OS between patients with different CLDN6 expression and ferroptosis score in TCGA. **K** Survival analysis showed differences in OS between patients with different CLDN6, NRF2, GPX4 expression and C&F classifier in TMA.

Our previous data showed that CLDN6, as a suppressor gene, is underexpressed in breast cancer and here our validation of CLDN6 expression extends to RNA array and single-cell sequencing datasets. We found that CLDN6 was highly expressed in normal luminal epithelial but was underexpressed in breast cancer cell, and showed tissue specificity in various tumors and normal (Fig. 1A, B and S1A). A TMA exhibited low expression of CLDN6 in breast cancer compared to para-carcinoma tissues (Fig. 1C). WGCNA demonstrated that the gene co-expressed with CLDN6 was predominantly enriched in ferroptosis, glutathione metabolism, and pentose phosphate pathways (Fig. 1D, E and S1B). Furthermore, CLDN6 was positively correlated with the ferroptosis score as calculated by GSVA in breast cancer patients (Fig. 1F and Table. S7). We analyzed the expression and relationship of CLDN6 and ferroptosis-related genes (NRF2 and GPX4) in TMA of breast cancer. The representative images and clinicopathological features are shown in Fig. 1G and H. Notably, patients with low CLDN6 expression had higher NRF2 expression (Fig. 1I). These results indicated that CLDN6 was underexpressed and associated with ferroptosis in breast cancer patients.

Next, we analyzed the impact of CLDN6 integrated with ferroptosis on the prognosis, and found that patients with high CLDN6 expression and high ferroptosis score had more favorable prognosis (Fig. 1J, K). Accordingly, we defined the classification pattern integrating CLDN6 expression and ferroptosis score as the CLDN6 & ferroptosis (C&F) classifier. The C&F classifier demonstrated strong predictive power in TCGA and the TMA of breast cancer, outperforming the CLDN6 expression and ferroptosis in robustness (Fig. S2A). Cox survival analysis verified that the C&F classifier was an independent predictor, and demonstrated exceptional predictive accuracy when used with clinicopathological features in TCGA and TMA (Fig. S2B-C and Table. S8-9). The aforementioned results showed that the integration of CLDN6 and ferroptosis has prognostic significance.

### CLDN6 triggers ferroptosis in breast cancer cells

**Fig. 2:**
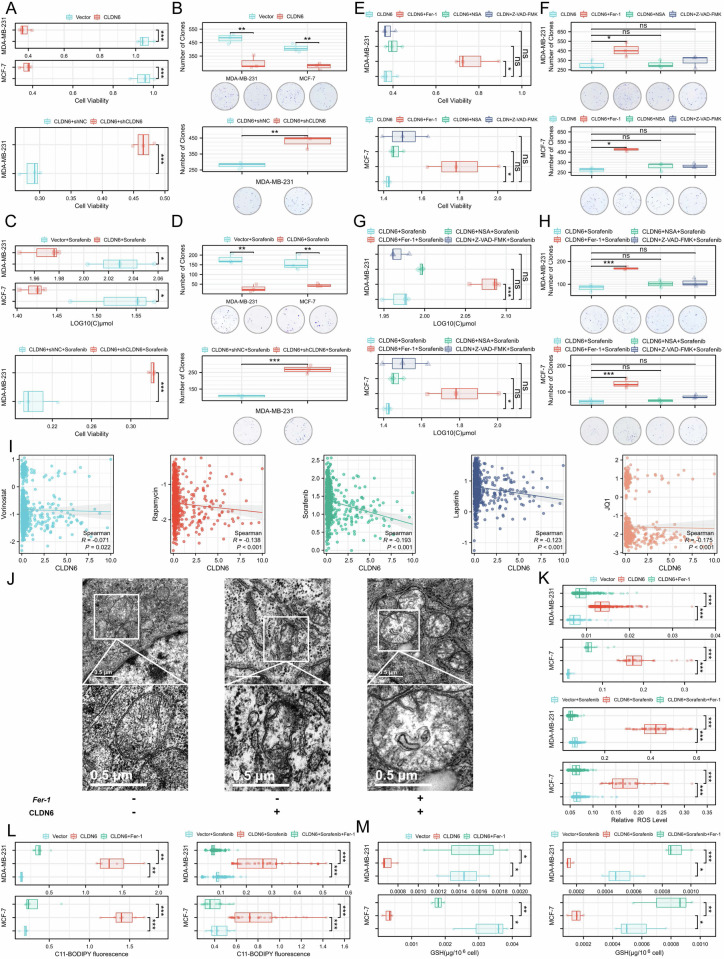
CLDN6 triggers ferroptosis in breast cancer cells. **A** Cell viability was measured in the indicated cells. **B** Clone formation assay in the indicated cells. **C** Cell viability was measured and IC50 was calculated by treating the indicated cells with an increased dose of sorafenib for 24 h. **D** Clone formation assay with sorafenib treatment in the indicated cells. **E** Cell viability was measured and IC50 was calculated, and Fer-1 (5 μM), NSA (10 μM), and Z-VAD-FMK (50 μM) were added to the indicated cells and treated for 24 h. **F** Clone formation assay in the indicated cells. **G** Cell viability was measured and IC50 was calculated by treating the indicated cells with an increased dose of sorafenib for 24 h. Meanwhile, Fer-1 (5 μM), NSA (10 μM), and Z-VAD-FMK (50 μM) were added to the indicated cells and treated for 24 h. **H** Clone formation assay in the indicated cells. **I** Correlation between CLDN6 and FINs. **J** The subcellular structure of MDA-MB-231 treated with Fer-1 (5 μM) was observed by TEM. Scale bar: 0.5 μm (**K**) ROS was measured by DCFH-DA staining. **L** Lipid peroxidation was measured by BODIPY-C11 staining. **M** GSH levels were measured in the indicated cells. ns, no significance, *P < 0.05, **P < 0.01, ***P < 0.001.

To ascertain the impact of CLDN6 on ferroptosis, cell death was first assessed using CCK-8 and plate clone formation assay in breast cancer cells. We found that CLDN6 inhibited cell viability, number of clones, which could be restored by silencing CLDN6 (Fig. 2A, B). Subsequently, we examined the effect of CLDN6 on FINs sensitivity. We further discovered that CLDN6 increased the sensitivity to multiple FINs (sorafenib and RSL3), and silencing CLDN6 reversed the enhanced sensitivity to sorafenib (Fig. 2C, D and S3A). Interestingly, only the ferroptosis inhibitor Fer-1 reversed this effect, not the necrosis inhibitor NSA or the apoptosis inhibitor Z-VAD-FMK (Fig. 2E-H and S3A). According to the oncoPredict result, CLDN6 negatively correlates with the IC50 of several FINs, including sorafenib (Fig. 2I). These data indicated that ferroptosis was the predominant form of cell death induced by CLDN6.

The effect of CLDN6 on ferroptosis was further detected at ultrastructural and biochemical levels. We observed that overexpression of CLDN6 resulted in decreased mitochondrial volume and increased membrane density, along with increased ROS and lipid peroxidation, and decreased GSH levels. Fer-1 could reverse these changes in breast cancer cells. After multiple FINs treatment, we found that CLDN6 aggravated the biochemical processes changes of ferroptosis. These changes could be restored by Fer-1 (Fig. 2J-M and S3B-D). These data indicated that CLDN6 could cause ferroptosis in breast cancer cells.

### CLDN6 promotes the NRF2 nuclear export to induce ferroptosis by the AKT/GSK3β/FYN axis

**Fig. 3:**
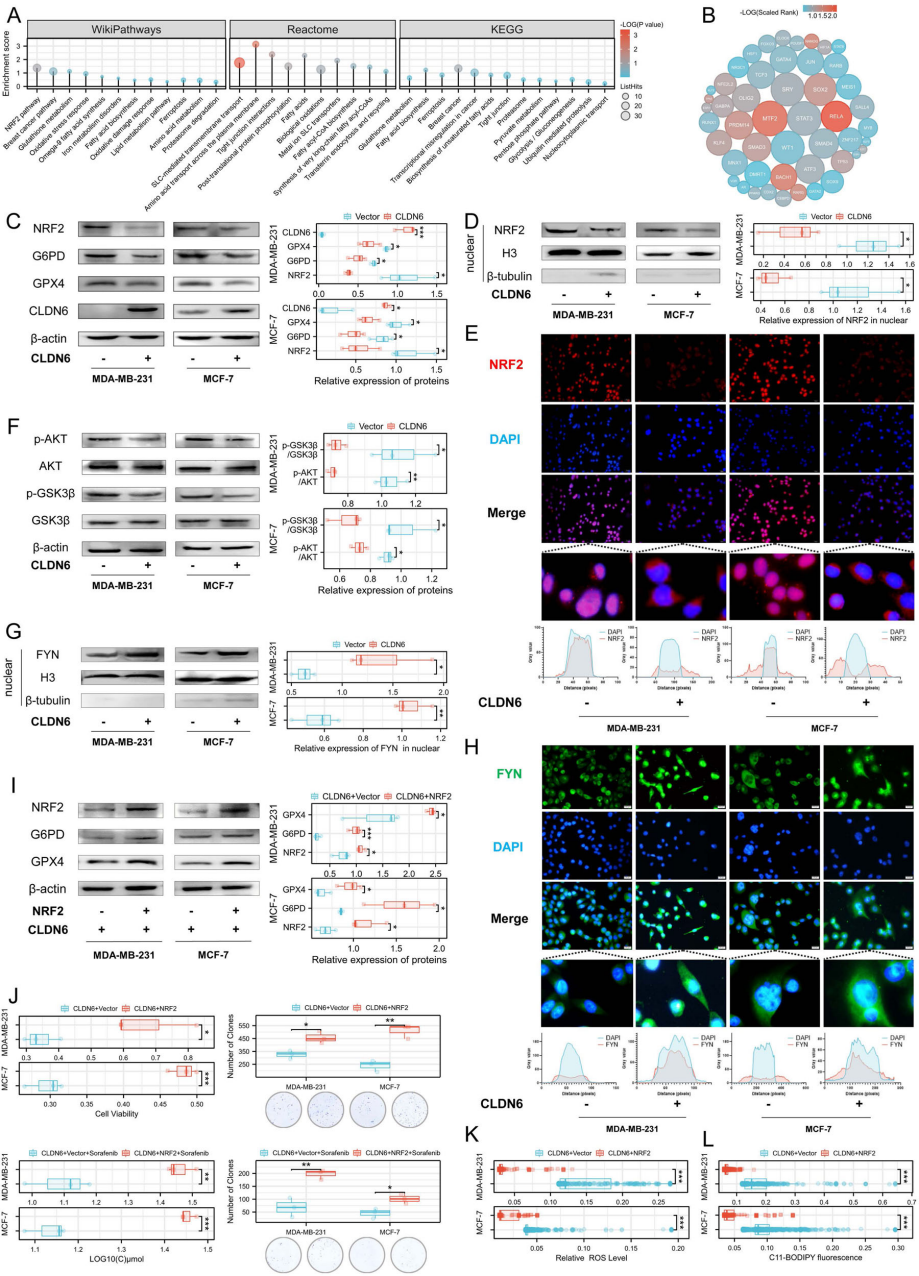
CLDN6 promotes the NRF2 nuclear export to induce ferroptosis by the AKT/GSK3β/FYN axis. **A** Results of enrichment analysis based on differentially expressed genes. **B** Predict and screen candidate transcription factors that regulate differentially expressed genes through ChEA3. **C** WB was used to assess NRF2, G6PD, GPX4, and CLDN6 expression levels in whole cell lysates from the indicated cells. **D** The NRF2 expression levels in the nuclear fraction from the indicated cells were analyzed using WB. **E** Fluorescent microscopy was used to observe the location of NRF2 in the indicated cells. Red: NRF2; Blue: DAPI. Scale bar: 50 μm. **F** and (**G**) WB was used to detect p-Akt (Ser473) and p-GSK3β (Ser9) and FYN in the nuclear fraction from the indicated cells. **H** FYN location in the indicated cells was observed using fluorescent microscopy. Green: FYN; Blue: DAPI. Scale bar: 50 μm. **I** NRF2, G6PD, and GPX4 expression levels from the indicated cells were assessed using WB. **J** Cell viability was measured and IC50 was calculated by treating the indicated cells with an increased dose of sorafenib for 24 h. Clone formation assay with sorafenib treatment. **K** ROS was measured by DCFH-DA staining. **L** Lipid peroxidation was measured by BODIPY-C11 staining. *P < 0.05, **P < 0.01, ***P < 0.001.

Further analysis of the C&F classifier revealed that the differentially expressed genes between High&High group and Low&Low group were mainly enriched in tight junction, ferroptosis, fatty acid biosynthesis, glutathione metabolism and NRF2 pathway (Fig. 3A and S4A). We also employed the ChEA3 to predict candidate transcription factors that regulate differential genes, such as NRF2 (Fig. 3B). The findings implied that NRF2 served as a potential intermediary between CLDN6 and ferroptosis. Our results showed that CLDN6 did not alter NRF2 mRNA levels, but reduced protein levels of NRF2 as well as its target genes, including GPX4 and G6PD (Fig. 3C and S4B). Silencing CLDN6 restored the protein levels of NRF2 and GPX4 (Fig. S4C). CLDN6 also reduced the protein level and localization of NRF2 in nucleus (Fig. 3D and E). These results indicated that CLDN6 inhibited the activation and nuclear localization of NRF2.

Export and import of NRF2 are significant factors influencing its nuclear localization. NRF2 is sequestrated by KEAP1 for degradation in the cytoplasm, and is imported into the nucleus for transcriptional activity when its Ser40 is phosphorylated [5, 20]. Our result showed that CLDN6 did not affect the phosphorylation level of NRF2 at Ser40 and the expression of KEAP1 (Fig. S4B). Therefore, we speculated that CLDN6 inhibited the activity of NRF2 by promoting its nuclear export. Extensive research has shown that the AKT/GSK3β/FYN axis is a vital signal for regulating NRF2 nuclear export [16–18]. Our data showed that CLDN6 decreased the phosphorylation of AKT on Ser473 residue and GSK3β on Ser9 residue, and increased the expression of FYN in nuclear (Fig. 3F-H). Thus, the findings implied that CLDN6 might promote the nuclear export of NRF2 via the AKT/GSK3β/FYN axis.

To determine whether the effects of CLDN6 on ferroptosis were dependent on NRF2, we overexpressed NRF2 in CLDN6-overexpressing breast cancer cells and detected NRF2 target genes. Our results showed that NRF2 increased the expression of GPX4 and G6PD (Fig. 3I). Further, we found that NRF2 decreased the sensitivity to multiple FINs, ROS, and lipid peroxidation levels (Fig. 3J-L and S4D-F). According to these results, CLDN6 induced ferroptosis through NRF2.

### CLDN6 regulates the axis AKT/GSK3β/FYN axis through PBK

**Fig. 4:**
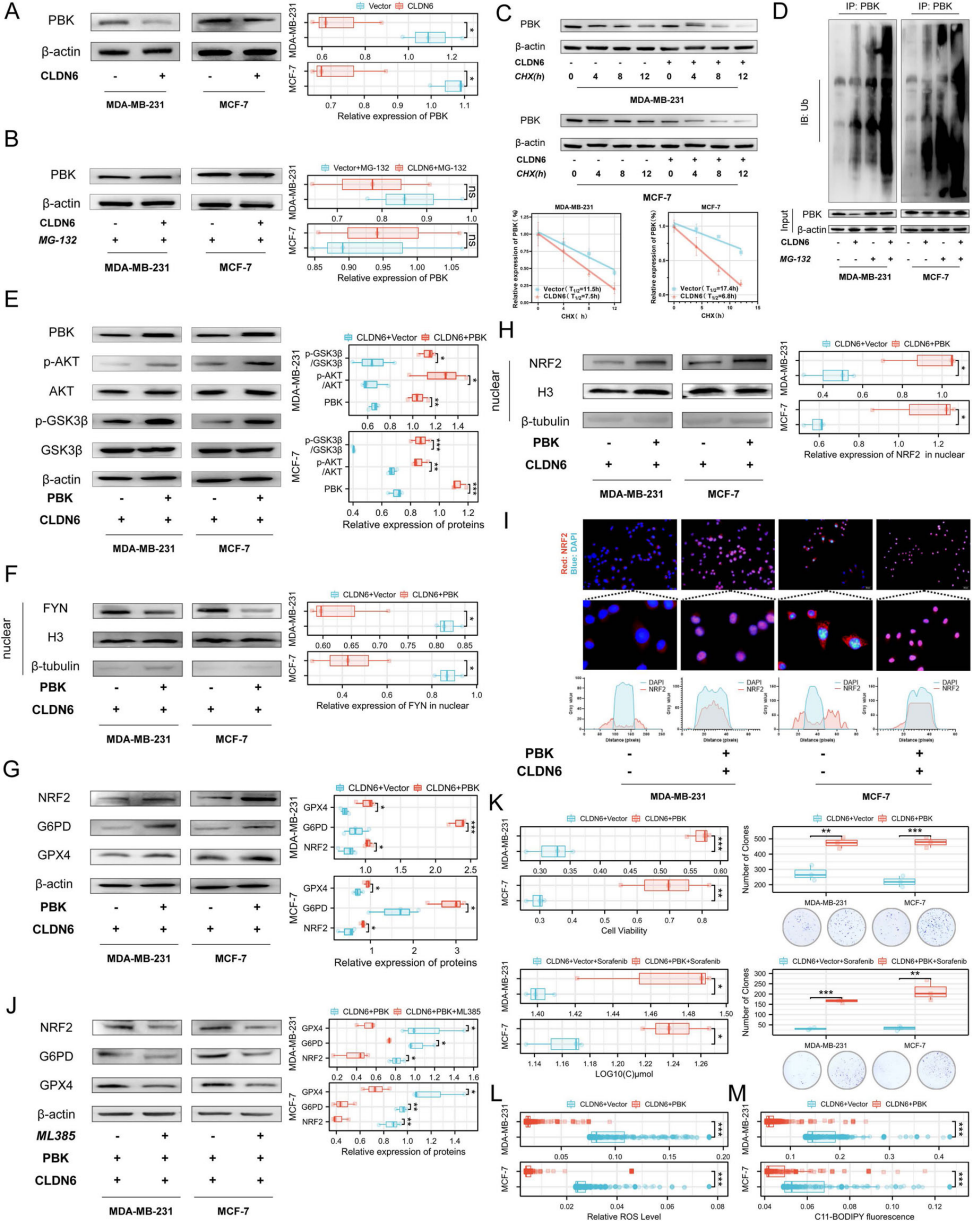
CLDN6 regulates the axis AKT/GSK3β/FYN axis through PBK. **A** PBK expression levels from the indicated cells were assessed using WB. **B** PBK expression levels from the indicated cells were assessed using WB. Cells are collected with 10 μM MG-132 processing for 24 h. **C** The half-life of PBK in the indicated cells was assayed. Cells were incubated with 10 μM cycloheximide (CHX) and lysed at indicated time points followed by WB. **D** The ubiquitination level of PBK in the indicated cells was analyzed. The cells were incubated with or without 10 μM MG-132 for 24 h and then lysed to immunoprecipitate with anti-PBK antibody followed by WB with an anti-ubiquitin antibody. **E** WB was used to detect p-Akt (Ser473) and p-GSK3β (Ser9) and PBK from the indicated cells. **F** WB was used to detect FYN in the nuclear fraction from the indicated cells. **G** NRF2, G6PD, and GPX4 expression levels from the indicated cells were assessed using WB. **H** The NRF2 expression levels in the nuclear fraction from the indicated cells were analyzed using WB. **I** NRF2 location in the indicated cells was observed using fluorescent microscopy. Red: NRF2; Blue: DAPI. Scale bar: 50 μm. **J** NRF2, G6PD, and GPX4 expression levels from the indicated cells were assessed using WB (ML385, 10 μM). **K** Cell viability was measured and IC50 was calculated by treating the indicated cells with an increased dose of sorafenib for 24 h. Clone formation assay with sorafenib treatment. **L** ROS was measured by DCFH-DA staining. **M** Lipid peroxidation was measured by BODIPY-C11 staining. ns, no significance, *P < 0.05, **P < 0.01, ***P < 0.001.

Research has shown that PBK leads to the activation of AKT [15]. We found that CLDN6 did not significantly affect the mRNA level, but decreased protein expression of PBK (Fig. 4A and S5A). The UPS-mediated degradation plays a significant role in the stability of the PBK protein [21]. Consequently, we investigated the effect of CLDN6 on PBK degradation through the UPS pathway. Our data showed that CLDN6 did not affect the protein level of PBK after treatment with the proteasome inhibitor MG-132 (Fig. 4B). We also found that CLDN6 significantly reduced the half-life of PBK, and increased the ubiquitination level of PBK, particularly when proteasomal degradation was inhibited (Fig. 4C and D). The above results showed that CLDN6 accelerated the degradation of PBK through the UPS.

Our results showed that overexpression of PBK increased the phosphorylation level of AKT and GSK3β, and decreased the FYN nuclear level (Fig. 4E and F). As we expected, PBK increased total protein, nuclear protein, nuclear localization of NRF2 and expression of G6PD and GPX4 (Fig. 4G-I). Then, we explored whether CLDN6 regulates G6PD and GPX4 via PBK in a NRF2-dependent manner. After using ML385, an inhibitor of NRF2, we found that the expression of NRF2, G6PD, and GPX4 was reduced in cells overexpressing CLDN6 and PBK (Fig. 4J). Further, our results showed that PBK decreased the sensitivity to multiple FINs, ROS, and lipid peroxide levels (Fig. 4K-M and S5B-D). Taken together, it appeared from these findings that CLDN6 regulates the AKT/GSK3β/FYN axis by PBK to induce NRF2-mediated ferroptosis.

### The interaction between CLDN6 and the DLG1/PBK complex necessitates the endosomal pathways

**Fig. 5:**
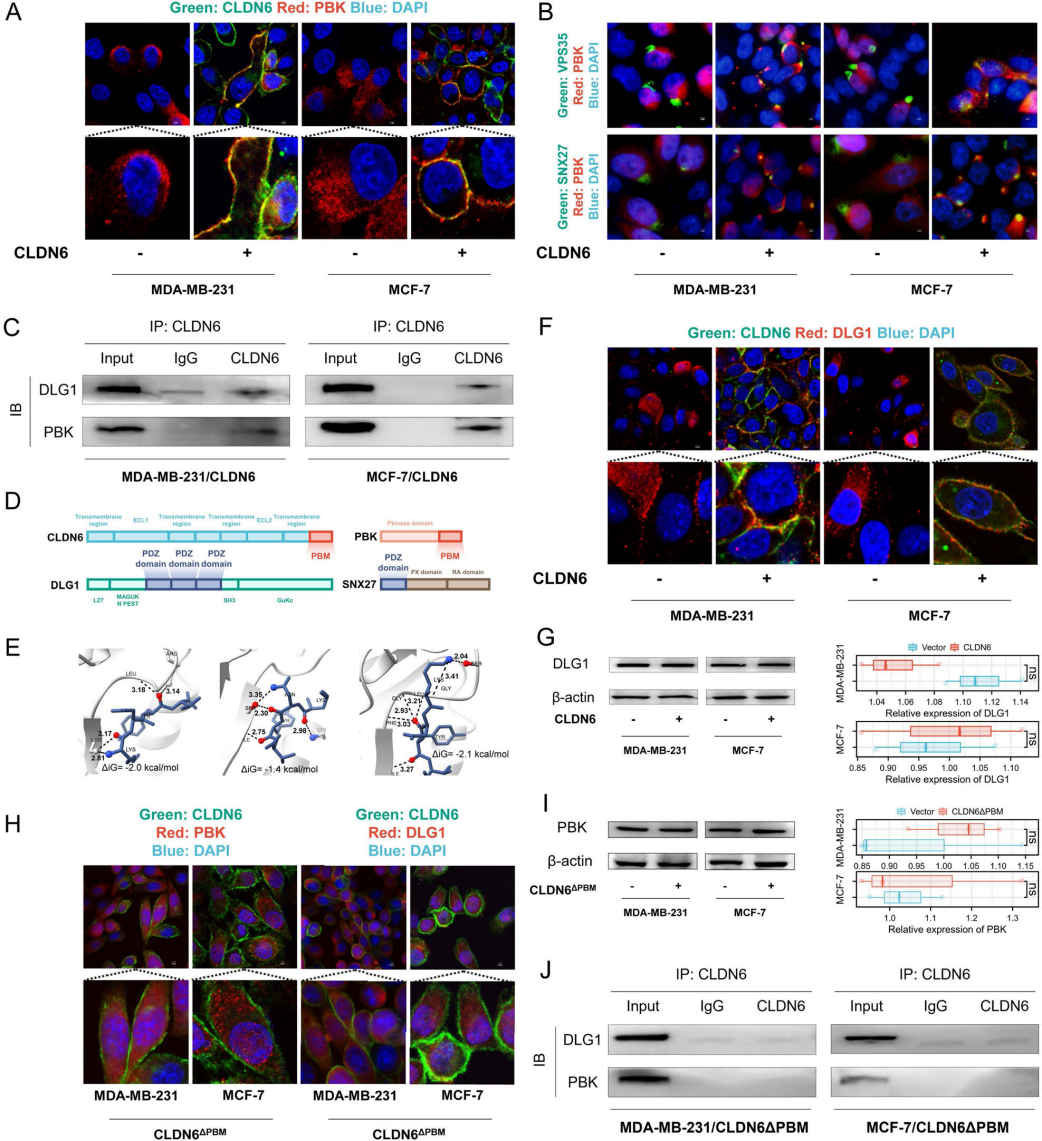
The interaction between CLDN6 and the DLG1/PBK complex necessitates the endosomal pathways. **A** The location of PBK and CLDN6 in the indicated cells was observed using fluorescent microscopy. Green: CLDN6; Red: PBK; Blue: DAPI. Scale bar: 3 μm. **B** VPS35, SNX27, and PBK location in the indicated cells was observed using fluorescent microscopy. Green: VPS35 or SNX27; Red: PBK; Blue: DAPI. Scale bar: 3 μm. **C** The interaction of CLDN6 and PBK or DLG1 was detected by co-IP assay in the indicated cells. **D** Schematic diagram showed the structure of CLDN6, DLG1, PBK and SNX27. **E** Molecular docking of PBM of CLDN6 with three PDZ domains of DLG1. Moreover, the tyrosine, asparagine, and lysine in the PBM of CLDN6 formed hydrogen bond interactions with the PDZ domain of DLG1 protein, respectively. **F** The location of DLG1 and CLDN6 in the indicated cells was observed using fluorescent microscopy. Green: CLDN6; Red: DLG1; Blue: DAPI. Scale bar: 3 μm. **G** DLG1 expression levels from the indicated cells were assessed using WB. **H** The location of PBK, DLG1 and CLDN6 in the indicated cells was observed using fluorescent microscopy. Green: CLDN6; Red: PBK or DLG1; Blue: DAPI. Scale bar: 3 μm. **I** PBK expression levels from the indicated cells were assessed using WB. **J** The interaction of CLDN6 and PBK or DLG1 was detected by Co-IP assay in the indicated cells. ns, no significance.

Research has established that the subcellular localization of PBK played a key role in its function [22–24]. It was surprising to find that CLDN6 co-localized with PBK on the cell membrane (Fig. 5A and S6A). Trafficking and recycling of endosomes to the cell membrane requires the involvement of VPS35 (which forms the retromer complex) and SNX27 (which sorts proteins through its PDZ domain) [13, 25, 26]. It has been predicted that PBK binds to SNX27 via PBM [14]. Therefore, we examined the co-localization of PBK with the VPS35-positive retromer complex and SNX27. We found that CLDN6 promotes their colocalization (Fig. 5B and S6B). These results indicated that CLDN6 trafficked PBK to the cell membrane by promoting the retromer complex mediated-endosomal pathway.

To explore the specific mechanism behind the co-localization of CLDN6 and PBK, we performed co-IP assays and found that PBK had binding affinities for CLDN6 (Fig. 5C). Structural analysis revealed that both CLDN6 and PBK contained PBM, while DLG1 contained three PDZ domains (Fig. 5D). We found that the PBM of CLDN6 bound stably to the PDZ domain of DLG1 according to the binding free energy by molecular docking (Fig. 5E). Our data suggested that CLDN6 failed to increase DLG1 expression, but could be integrated with DLG1 and co-located on the cell membrane (Fig. 5C, F, G and S6C). These data suggested that CLDN6 recruited DLG1/PBK complex to the cell membrane.

To clarify the role of PBM in CLDN6 linking DLG1 to PBK, we transfected PBM-deficient CLDN6 in breast cancer cells. We observed that PBM-deficient CLDN6 was no longer co-located with DLG1 or PBK (Fig. 5H and S6A-C). PBM-deficient CLDN6 did not affect the PBK protein level and was unable to bind DLG1 or PBK (Fig. 5I and J). This highlights that the PBM of CLDN6 was indispensable in the recruitment of DLG1/PBK complex. The above results provided evidence that CLDN6 recruited PBK to the cell membrane by endosomal pathway and bound with the DLG1/PBK complex.

### CLDN6 triggers breast cancer to undergo ferroptosis in vivo

**Fig. 6:**
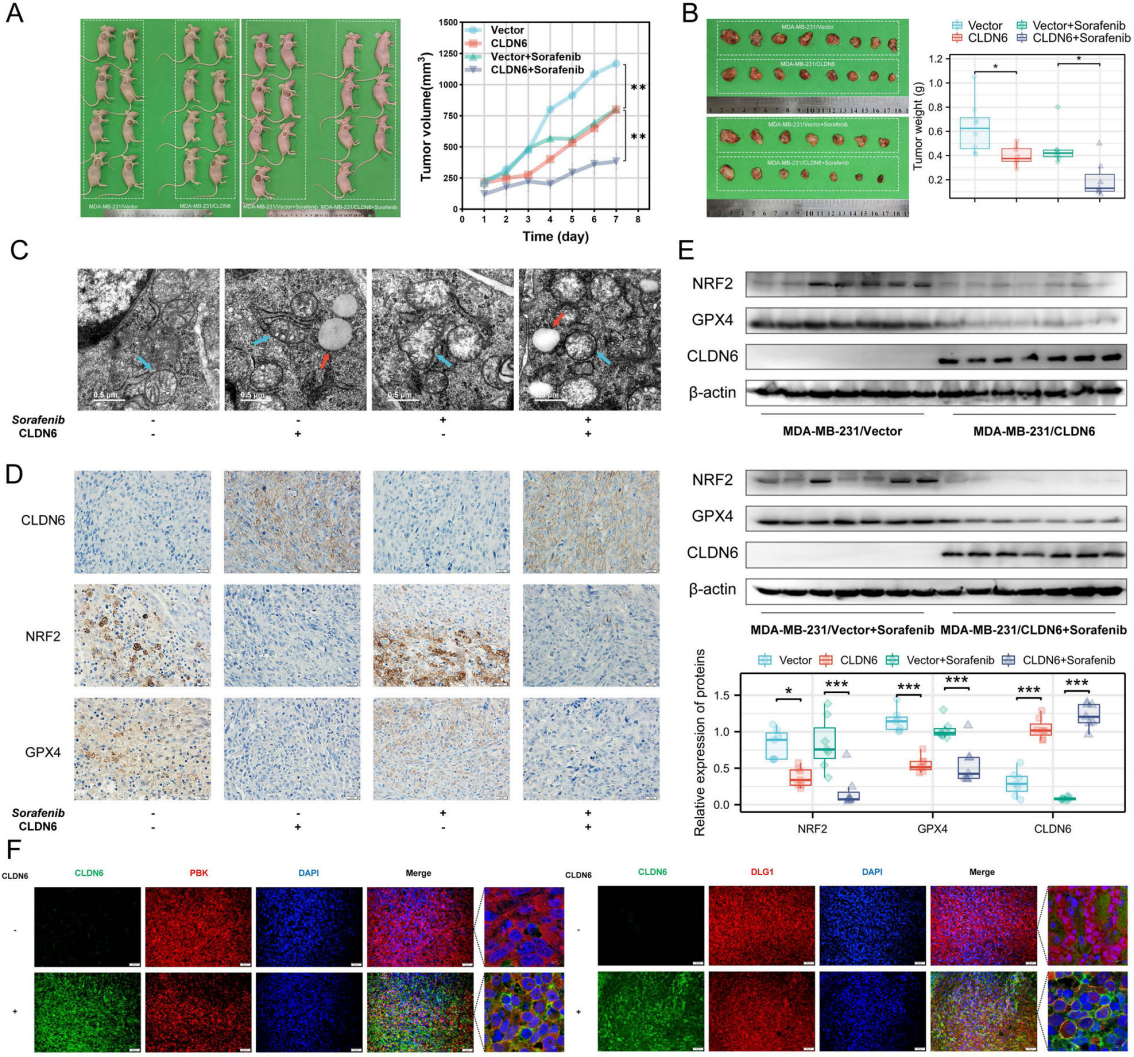
CLDN6 triggers breast cancer to undergo ferroptosis in vivo. **A** and (**B**) The BALB/c nude mice were implanted into MDA-MB-231/Vector or MDA-MB-231/CLDN6 cells. After 10 d of implantation, mice were given 15 mg/kg sorafenib by gavage every day. After 7 d of the administration, mice were killed and the tumor weight was measured. **C** The subcellular structure of the xenograft tumor was observed by TEM. The mitochondria were shown by the blue arrow. Scale bar, 0.5 μm. **D** The representative IHC images showed CLDN6, NRF2, and GPX4 in xenograft tumor tissue. Scale bar, 20 μm. **E** The protein levels of CLDN6, NRF2, and GPX4 in tumor tissue of the xenograft tumor model were detected by WB. **F** PBK, DLG1, and CLDN6 location in the indicated cells was observed using fluorescent microscopy. Green: CLDN6; Red: PBK or DLG1; Blue: DAPI. Scale bar: 50 μm (left), 3 μm (right). *P < 0.05, **P < 0.01, ***P < 0.001.

To evaluate the effect of CLDN6 on ferroptosis in vivo, we established a subcutaneous xenograft model in nude mice. We found that the volume and weight of tumors in the CLDN6 overexpression group were smaller than those in the control group, and sorafenib treatment exacerbated these changes (Fig. 6A and B). Additionally, sorafenib treatment was well tolerated in nude mice by body weight and H&E staining (Fig. S7A and B). CLDN6 caused an increase in mitochondrial membrane density and lipid droplet, and a decrease in mitochondrial cristae, which were aggravated after treatment with sorafenib as observed by TEM in xenograft tumor tissue (Fig. 6C). These results indicated that CLDN6 induced ferroptosis in vivo.

IHC staining and WB in xenograft tumor tissues revealed lower NRF2 and GPX4 expressions in the CLDN6 overexpression and sorafenib-treated CLDN6 overexpression groups compared to controls (Fig. 6D and E). IF staining showed CLDN6, DLG1, and PBK co-located on the cell membrane (Fig. 6F). The above findings suggested that CLDN6 induced ferroptosis and inhibited NRF2 and GPX4 expression in vivo.

## Discussion

Increasingly, the anticancer effect of ferroptosis in breast cancer has attracted attention. We have previously identified CLDN6 as a pivotal tumor suppressor in breast cancer [6]. Nonetheless, there have been no studies that integrate CLDN6 and ferroptosis to predict prognosis, and the impact of CLDN6 on ferroptosis is still elusive in breast cancer. In this study, we confirmed the prognostic significance of integrating CLDN6 with ferroptosis, and elucidated a novel mechanism by which CLDN6 triggers NRF2-mediated ferroptosis in breast cancer.

Given our finding that CLDN6 is associated with ferroptosis in breast cancer patients, CLDN6 triggering ferroptosis in breast cancer cells was subsequently observed in terms of cell viability, ultrastructure, and biochemical processes. Ferroptosis was the predominant form of cell death induced by CLDN6. Our results supported the view that CLDN6 was associated with ferroptosis in xenograft tumor models. Here we proposed for the first time that ferroptosis was an integral link for CLDN6 to exert the anticancer effect in breast cancer. The integration of CLDNs family members with other features has proven effective for prognostic predictions in breast cancer [27, 28]. The emerging view is that ferroptosis exhibits certain predictive prognostic power in breast cancer [3]. While CLDN6 showed limited predictive capacity independently [29], the integration of CLDN6 with ferroptosis through the application of C&F classifier enabled a more precise differentiation of patients with varying prognoses. We noted the presence of patients with low CLDN6 and high ferroptosis scores and patients with high CLDN6 and low ferroptosis scores. However, the distribution of these samples was not concentrated and did not have a decisive effect on the overall correlation of CLDN6 and ferroptosis scores in the population.

NRF2, a transcriptional co-activator, is crucial for inhibiting ferroptosis [5]. This research predicted that NRF2 served as a potential intermediary between CLDN6 and ferroptosis. However, the regulation of NRF2 by CLDN6 remains unexplored. The nuclear export is essential for the inactivation of NRF2 [19, 30]. The AKT/GSK3β/FYN axis is well-studied for regulating NRF2 nuclear export in non-cancerous [17, 18, 31], but is poorly understood in cancer, particularly breast cancer. Here we found that CLDN6 promotes the NRF2 nuclear export to induce ferroptosis by the AKT/GSK3β/FYN axis in breast cancer cells. Multiple members of the CLDNs family, such as CLDN5, 7, and 18, can inhibit the phosphorylation of AKT [32]. We have previously demonstrated that CLDN6 inhibits AKT phosphorylation by upregulating PTEN in colorectal cancer [33]. Our findings revealed a novel mechanism by which CLDN6 regulated NRF2 nuclear export through the AKT/GSK3β/FYN axis.

According to current views, PBK activates AKT, and its function is influenced by subcellular localization [15, 24]. Here we found that CLDN6 regulated the AKT/GSK3β/FYN axis by PBK to induce NRF2-mediated ferroptosis. Remarkably, CLDN6 did not change the mRNA levels but reduced the protein expression of PBK, suggesting that CLDN6 primarily affected PBK protein stability. As we discovered, CLDN6 accelerated PBK degradation by the UPS. Noteworthy, the E3 ubiquitin ligase LNX1 localizes at the membrane of breast cancer in HPA [34], and has been shown to mediate the degradation of the PBK by the UPS [35]. Whether LNX1 is involved in the promotion of PBK degradation by CLDN6 is the focus of our future studies.

Another important finding was that CLDN6 inhibited PBK and co-localizes with PBK at the cell membrane, which has not been previously described. One of our interesting findings was that CLDN6 drove PBK to the cell membrane by enhancing SNX27 mediated-endosomal pathway. Actin is reported to be involved in SNX27-mediated endosomal transport [36]. We have previously shown that CLDN6 promoted the rearrangement of actin in breast cancer cells [9]. Consequently, we hypothesize that actin may act as a conduit for CLDN6 to regulate the endosomal pathway and traffics PBK to the cell membrane, which still needs to be confirmed by subsequent experiments.

PBM of CLDN6 is an important structure to exert signal transduction in tumor progression [6]. There is evidence that CLDN6 interacted with JNK via its PBM, and then enhanced the activation of JNK/c-Jun signaling [9]. Here we showed that the PBM of CLDN6 was essential for recruiting the DLG1/PBK complex. As a scaffold protein, DLG1 forms a complex with PBK through its PDZ2 domain, potentially bridging CLDN6 and PBK [10]. Our results indicate that CLDN6 depended on its PBM to form a complex with DLG1 and PBK at the cell membrane. It is the first study to reveal the mechanism of CLDN6 recruiting PBK through the endosomal pathway and binding with DLG1/PBK complex, thereby increasing ferroptosis. Further research should explore how DLG1 bridges CLDN6 and PBK.

In conclusion, we confirm the prognostic significance of integrating CLDN6 with ferroptosis, and reveal that CLDN6 has a previously unrecognized function and mechanism in triggering NRF2-mediated ferroptosis. This study provides a reliable biomarker for predicting prognosis and is anticipated to guide the selection of therapies targeting ferroptosis in breast cancer.

## Supplementary information

SUPPLEMENTAL MATERIAL

Original western blot data

## Data Availability

The analyzed datasets generated during the present study are available from the corresponding author on reasonable request.
