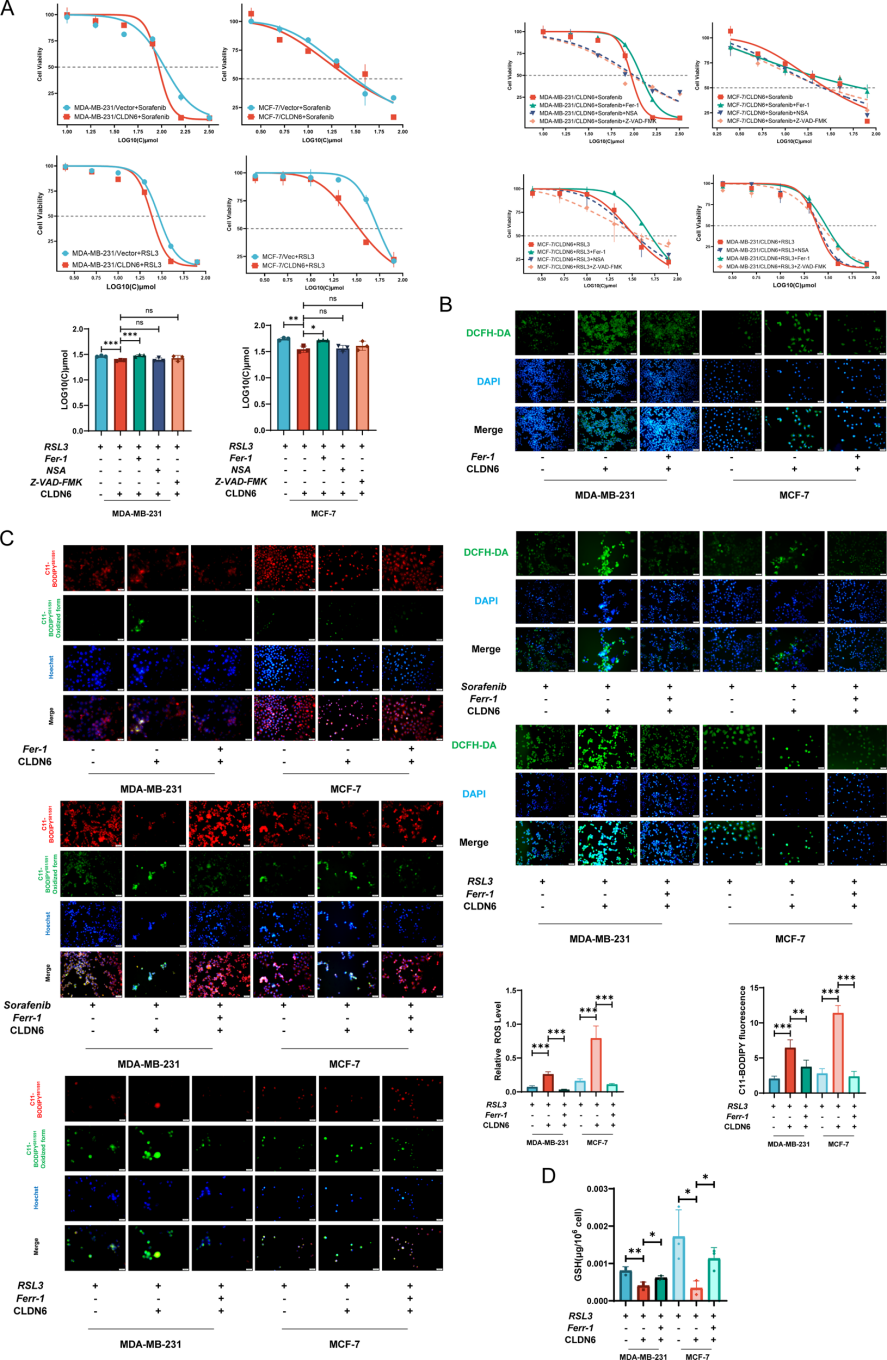# Correction: CLDN6 triggers NRF2-mediated ferroptosis through recruiting DLG1/PBK complex in breast cancer

**DOI:** 10.1038/s41419-025-07910-8

**Published:** 2025-08-21

**Authors:** Da Qi, Yan Lu, Huinan Qu, Yuan Dong, Qiu Jin, Minghao Sun, Chengshi Quan

**Affiliations:** 1 The Key Laboratory of Pathobiology, Ministry of Education, College of Basic Medical Sciences, Jilin University, 126 Xinmin Avenue, Changchun, 130021 China; 2 Department of Anatomy, College of Basic Medical Sciences, Jilin University, 126 Xinmin Avenue, Changchun, 130021 China; 3 Department of Histology and Embryology, College of Basic Medical Sciences, Jilin University, 126 Xinmin Avenue, Changchun, 130021 China

**Keywords:** Breast cancer, Cell death, Prognostic markers, Tumour biomarkers, Protein translocation

Correction to: *Cell Death and Disease* 10.1038/s41419-025-07448-9, published online 21 February 2025

Upon reviewing our published work, we realized that we mistakenly used incorrect Fig. S3 B and C. Due to our oversight, the same images were used for DCFH-DA staining of RSL3-treated MDA-MB-231 cells and MCF-7 cells in Fig. S3 B. In Fig. S3 C, the same images were used for C11 staining of MCF-7 cells without ferroptosis inhibitor and MDA-MB-231 cells treated with sorafenib group. We have revised the two errors and shown the correction images in the corrected file. Critically, the statistical analysis and conclusion of the article are based on the correct original figures, and these corrections do not affect the validity or interpretation of the reported findings.