# Supplementary material for: CLDN6 triggers NRF2-mediated ferroptosis through recruiting DLG1/PBK complex in breast cancer
Source: Cell Death Dis. 2025 Feb 21;16(1):122. doi: 10.1038/s41419-025-07448-9 (PMC11845765; doi:10.1038/s41419-025-07448-9)
Supplement: Supplementary file 1 — SUPPLEMENTAL MATERIAL [file 41419_2025_7448_MOESM1_ESM.doc]

# Supplementary Material

**CLDN6 triggers NRF2-mediated ferroptosis through recruiting DLG1/PBK complex in breast cancer**

Da Qi^1^, Yan Lu^2^, Huinan Qu^3^, Yuan Dong^1^, Qiu Jin^1^, Minghao Sun^1^, Chengshi Quan^1^, *

**Author information:** ^1^ The Key Laboratory of Pathobiology, Ministry of Education, College of Basic Medical Sciences, Jilin University, 126 Xinmin Avenue, Changchun 130021, China.

^2^ Department of Anatomy, College of Basic Medical Sciences, Jilin University, 126 Xinmin Avenue, Changchun 130021, China.

^3^ Department of Histology and Embryology, College of Basic Medical Sciences, Jilin University, 126 Xinmin Avenue, Changchun 130021, China.

*Corresponding author.

Chengshi Quan, Email: quancs@jlu.edu.cn

**Supplementary Methods**

#### Single-cell sequencing, WGCNA, GSVA, enrichment and drug sensitivity analyses

The expression data of CLDN6 in breast cancer and other tumors were downloaded from GENT2 database (Table.S5-6) [1]. Single-cell sequencing data and cell annotation were downloaded from CellMarker 2.0 [2]. A WGCNA R package was used to construct mRNA co-expression networks in The Cancer Genome Atlas (TCGA) breast cancer cohort [3]. Among all soft thresholds (β) with R^2^ > 0.802, we selected the automatic value β (β = 10) returned by the WGCNA pick Soft Threshold function. The network merge height was set at 0.25. We used default settings for other WGCNA parameters. Enrichment analysis was conducted on all identified differentially expressed genes using the cluster Profiler Package (version 3.14.3). The ferroptosis related gene set was selected based on the published literature, and the gene set was scored using the “GSVA” R package. All gene sets are shown in Table S7. Ferroptosis score = score of promoting ferroptosis gene set -score of inhibiting ferroptosis gene set. Patients in the breast cancer tissue microarray were sampled from September 2012 to December 2013, and all patients were sampled before treatment. We defined patients with NRF2 and GPX4 expression below the median as the high ferroptosis group in the TMA. As criteria for selecting genes with differential expression, we applied *P* value < 0.05 and |log2 fold change (FC)| > 0.4 using the “DESeq2” R package. We conducted Kyoto Encyclopedia of Genes and Genomes (KEGG), Reactome and WikiPathways analysis using “clusterProfiler” package. Patient sensitivity to ferroptosis inducers (FINs) was predicted by the OncoPredict R package [4].

#### Cell culture

Human breast cancer cell lines (MDA-MB-231 and MCF-7) and human embryonic renal epithelial cell lines (293T) were purchased from Zhongqiao Xinzhou Biotechnology Limited company. Human breast cancer cell lines (MDA-MB-231 and MCF-7), and HEK293T were cultured in Dulbecco’s modified Eagle’s medium (Meilune, China) containing 10% fetal bovine serum (Gibco, USA) at 37 °C in a humidified incubator containing 5% CO_2_.

#### Transfection

The CLDN6, NRF2, and PBK overexpression plasmids and silencing CLDN6 plasmids were generated by PPL Genebio Technology (Genebio Technology, China). We followed the procedure described previously for transfection [5].

#### Cell counting kit-8 (CCK-8)

Measurement of cell viability was conducted with CCK-8 reagents (Meilune, China). Incubation took place for 24 h after seeding 1000 cells per well in 96-well plates. Each well was incubated at 37°C in 5% CO_2_ for 2 h with a 1:19 diluted CCK-8 solution in DMEM. Microplate readers (Thermo, Germany) were used to measure the absorbance at 450 nm.

#### Plate clone formation assay

Plating of 600 cells per well was done in triplicate in 6-well plates. A fresh medium was replaced every 2-3 d until colonies were visible. After washing, fixing, staining, and counting the colonies, the colonies were counted.

#### In vivo tumor xenograft model

A total of 30 female BALB/cA-nu mice (4 w old, 16-20 g, specific pathogen-free standard) were purchased from Beijing Huafukang Company and all animals were kept in laboratory animal centers, in accordance with the regulations of the Medical Ethics Committee. This experiment was carried out using BALB/c nude mice that passed quality control tests. Laboratory animals were cared for and used in accordance with applicable institutional and national guidelines and regulations. Each nude mouse was randomly grouped and inoculated with 5 × 10^6^ cells in 100 µL PBS subcutaneously. When the tumor volume of all nude mice in each experiment was about 200 mm^3^, MDA-MB-231/Vector+Sorafenib and MDA-MB-231/CLDN6+Sorafenib groups were treated with 30 mg/kg sorafenib-sodium carboxycellulose suspension 300 μL by gavage once a day for 7 d. MDA-MB-231/Vector and MDA-MB-231/CLDN6 were treated with equal volume of sodium carboxycellulose suspension by gavage once a day for 7 d. Sorafenib was treated orally once a day for 7 d. The length (L), width (W), and high (H) of the tumor volume (V) and body weight of nude mice were measured daily after treatment with sorafenib. The tumor volume was estimated by the formula V = 6 × L × W × H/π. The transplanted tumors were fixed in 10% neutral formalin or glutaraldehyde for IHC, WB, TEM, and IF. The pathological changes of major organs in nude mice were detected using hematoxylin-eosin (H&E) staining.

#### RNA extraction and RT-PCR

As previously described, RNA was extracted and RT-PCR was conducted [6]. Primers were synthesized by Sangon (Sangon, China) and listed in Table.S2.

#### Western blot (WB)

Previously described WB assays were performed in this study using the antibodies mentioned in Table S3 [7].

#### Nuclear/cytosol fractionation

1 × 10^6^ cells were needed. A Nuclear/Cytosol Fractionation Kit (TransGen Biotech, China) was applied to isolate the nucleus and cytosol protein according to the manufacturer’s instructions.

#### Molecular docking

The protein crystal structures of DLG1 were obtained from the RCSB PDB database (https://www.rcsb.org/) (PDB ID: 3rl7, 3rl8 and 1pdr), and the downloaded structures were pretreated with water removal. Firstly, HPEPDOCK was used to perform peptide-protein flexible docking to adjust the initial conformation of the two proteins, and then the stability of the conformation was detected by the PDBePISA method.

#### Medical illustrations

The Figure was partly generated using Servier Medical Art, provided by Servier, licensed under a Creative Commons Attribution 3.0 unported license.

#### Statistics analysis

All statistical analyses in this study were conducted using R software and GraphPad Prism. Quantitative variables were analyzed by the t-test correlation test, and the Pearson χ2 test was used to compare qualitative variables. The protein expression levels and clinical characteristics were compared by the chi-square test. The survminer package was used to determine the optimal cut-off value. Cox regression and Kaplan–Meier analyses were performed via the survival package. The time ROC analysis was used to compare the predictive accuracy. Prognostic factors were determined with univariate and multivariate Cox regression analyses. The C-indices of different variables were compared using the CompareC package. Correlation coefficients were calculated by Spearman correlation analyses. Normal and non-normal variables were compared using the unpaired Student t-test and the Mann-Whitney U test, respectively. *P* < 0.05 was considered statistical significance.

#### Ethics approval and consent to participate

The study was conducted according to the guidelines of the Declaration of Helsinki, and approved by the Experimental Animal Ethical Committee of Jilin University (protocol code 2021.237 and 2022.72) and the Life Sciences Ethics Committee of Changsha Yaxiang Biotechnology Co., LTD.

**Supplementary Figure**


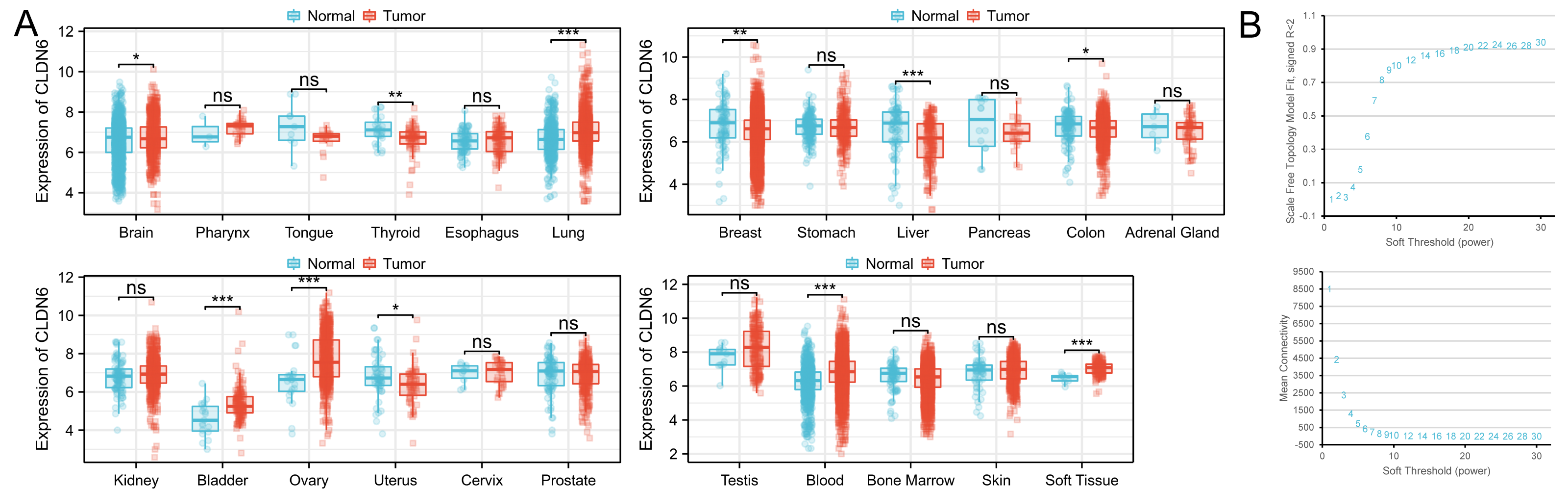


**Fig.S1 CLDN6 is associated with ferroptosis in breast cancer patients**

**(A)** Distribution of CLDN6 in tumor and normal tissue. **(B)** The scale-free fit index and mean connectivity for different selections of soft-thresholding powers (β). The soft threshold power β = 10 (scale free R2 = 0.802) was selected to construct a scale-free network. ns no significance, * *P* < 0.05, ** *P* < 0.01, *** *P* < 0.001.


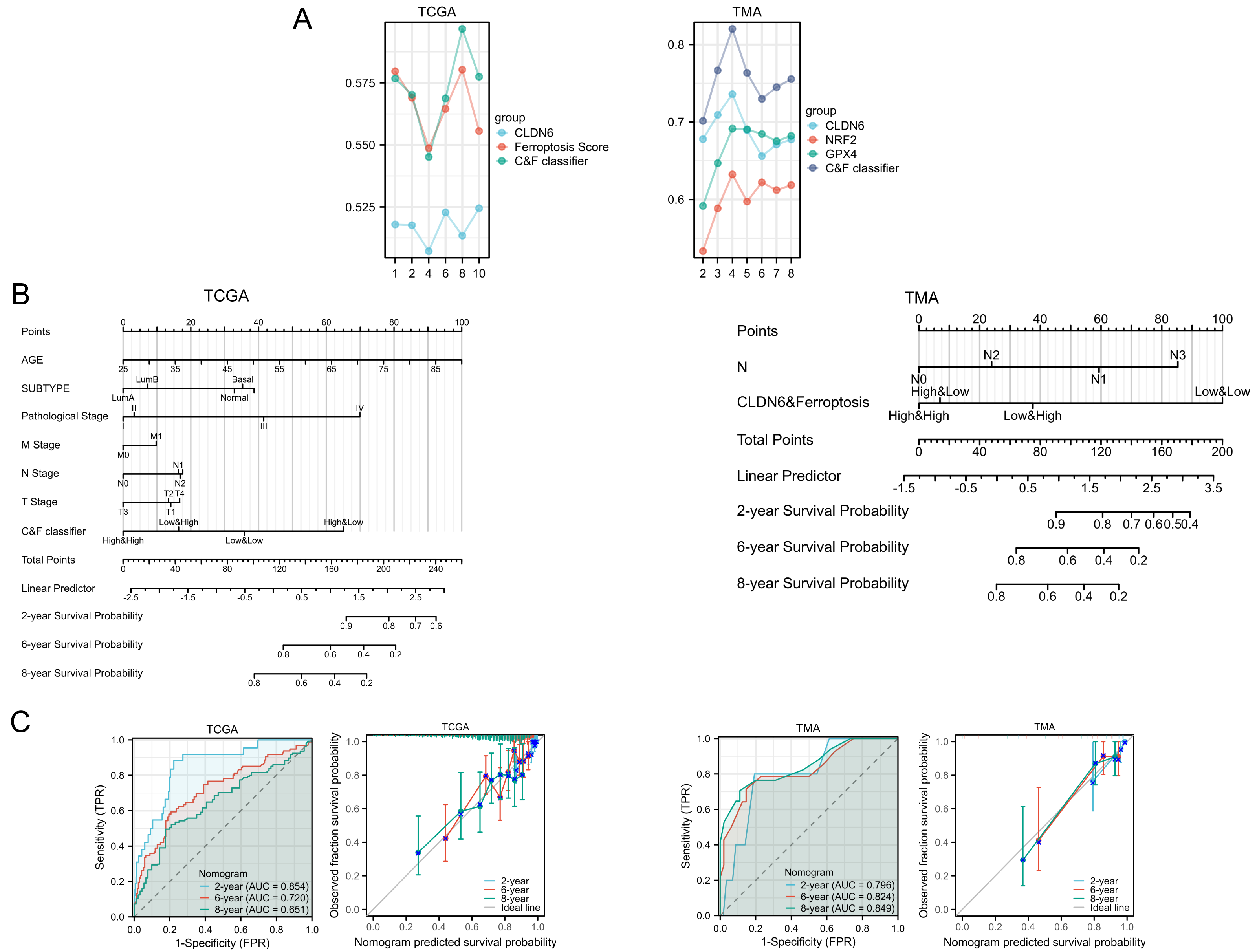


**Fig.S2 Prognostic significance of integrating CLDN6 with ferroptosis**

**(A)** The change of c-index of each variable with time in TCGA and TMA. **(B)** The nomogram was used to estimate the survival probabilities. **(C)** Time-dependent ROC curves and calibration curve of the nomogram.

**
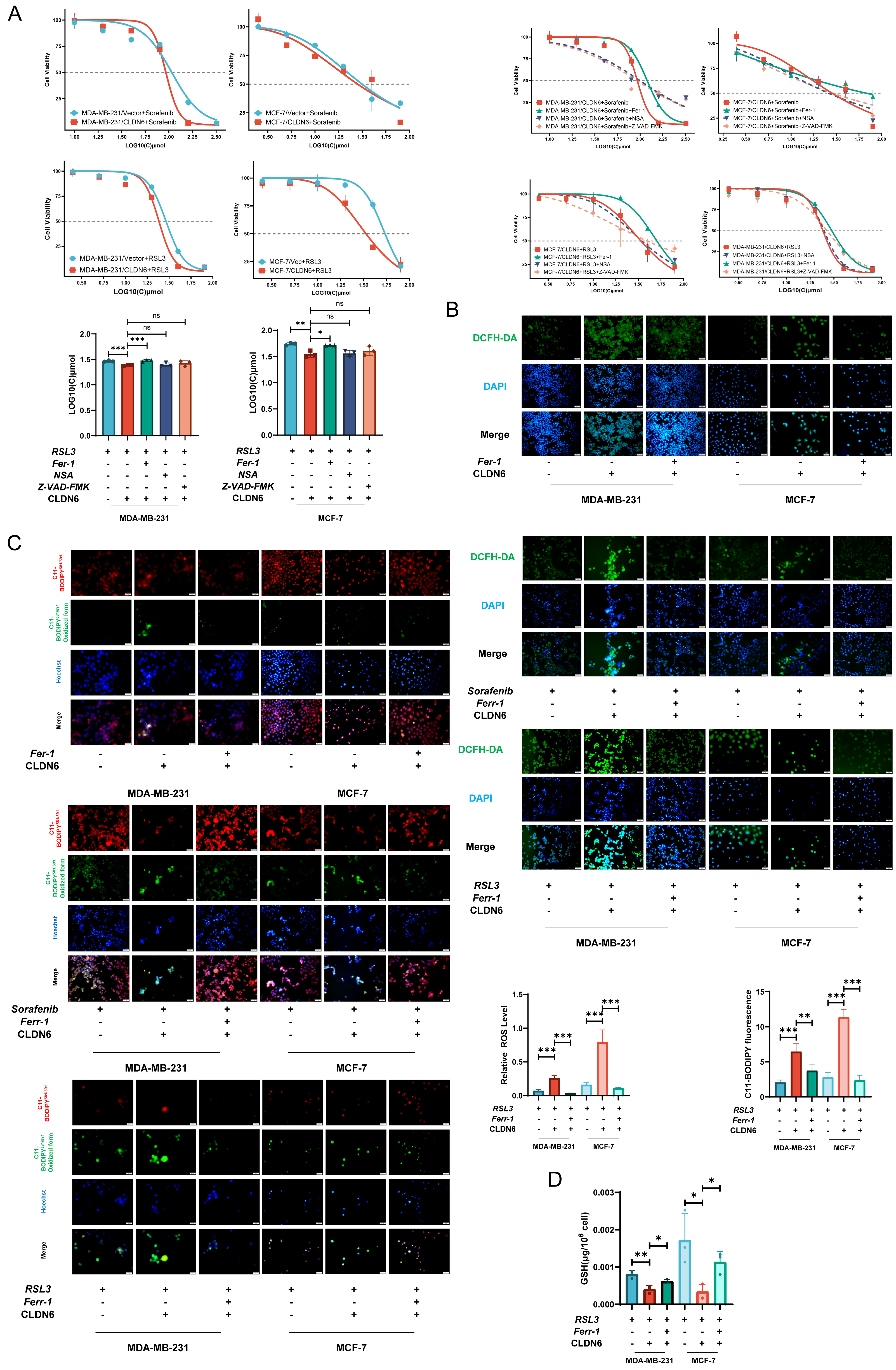
**

**Fig.S3 CLDN6 triggers ferroptosis in breast cancer cells**

**(A)** Cell viability was measured and IC50 was calculated by treating the indicated cells with an increased dose of sorafenib and RSL3 for 24 h. Meanwhile, Fer-1 (5 μM), NSA (10 μM), and Z-VAD-FMK (50 μM) were added to the indicated cells and treated for 24 h. **(B)** ROS was measured by DCFH-DA staining, Bar = 50μm. **(C)** Lipid peroxidation was measured by BODIPY-C11 staining, Blue: Hoechst, Green: BODIPY-C11 oxidized form, Red: BODIPY-C11, Bar = 50μm. **(D)** GSH levels were measured in the indicated cells. ns, no significance, * *P* < 0.05, ** *P* < 0.01, *** *P* < 0.001.


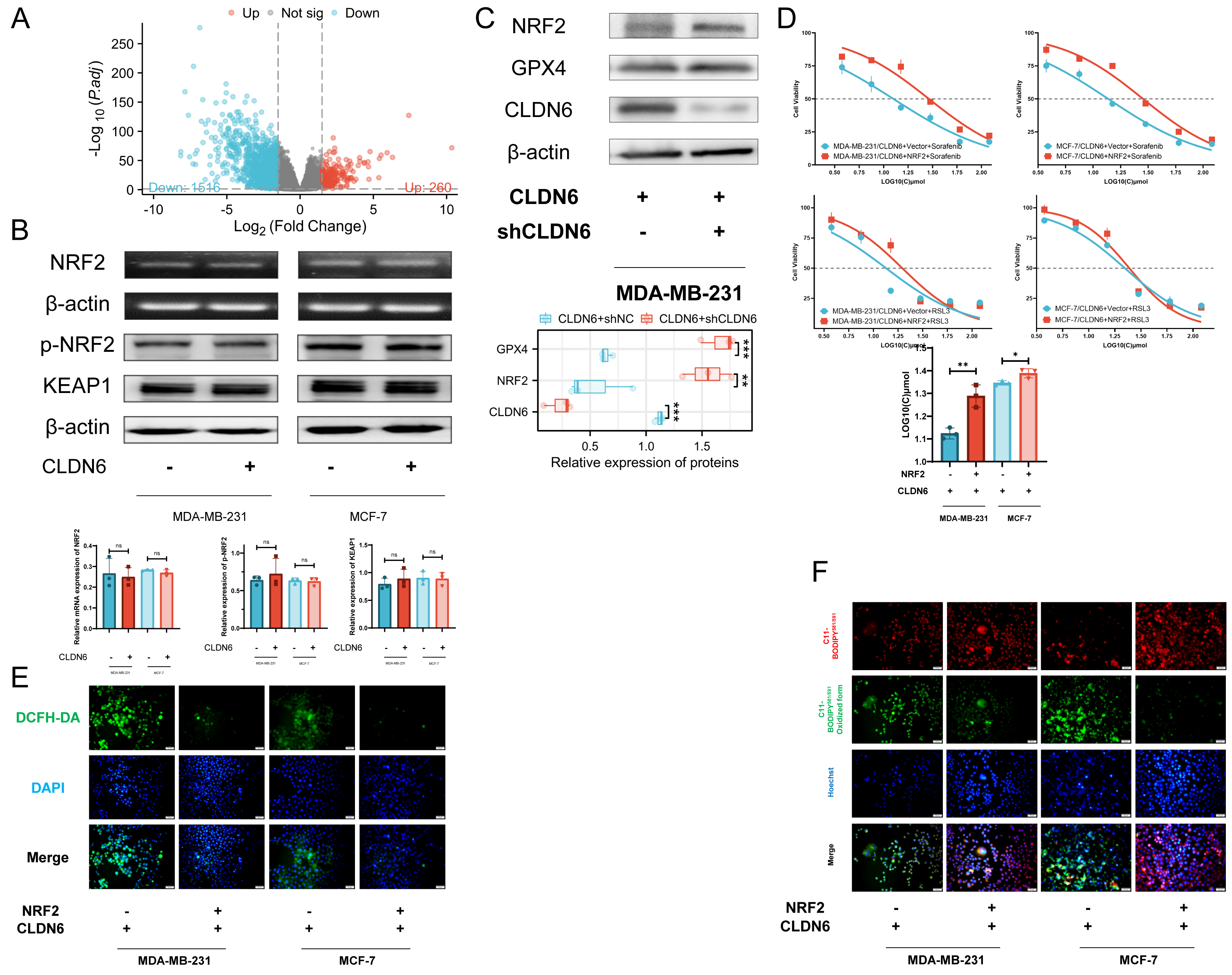


**Fig.S4 CLDN6 promotes the NRF2 nuclear export to induce ferroptosis by the AKT/GSK3β/FYN axis**

**(A)** Volcanic diagram showed differentially expressed genes, with 260 upregulated and 1516 downregulated. **(B)** NRF2 mRNA levels from the indicated cells were assessed using RT-PCR. NRF2, p-NRF2, and KEAP1 expression levels in the whole cell lysate from the indicated cells were assessed using WB. **(C)** NRF2, GPX4 and CLDN6 expression levels in the whole cell lysate from the indicated cells were assessed using WB. **(D)** Cell death was measured and IC50 was calculated by treating the indicated cells with an increased dose of sorafenib and RSL3 for 24 h. **(E)** ROS was measured by DCFH-DA staining, Bar = 50 μm. **(F)** Lipid peroxidation was measured by BODIPY-C11 staining, Blue: Hoechst, Green: BODIPY-C11 oxidized form, Red: BODIPY-C11, Bar = 50 μm. ns no significance, ** *P* < 0.01, *** *P* < 0.001.

­
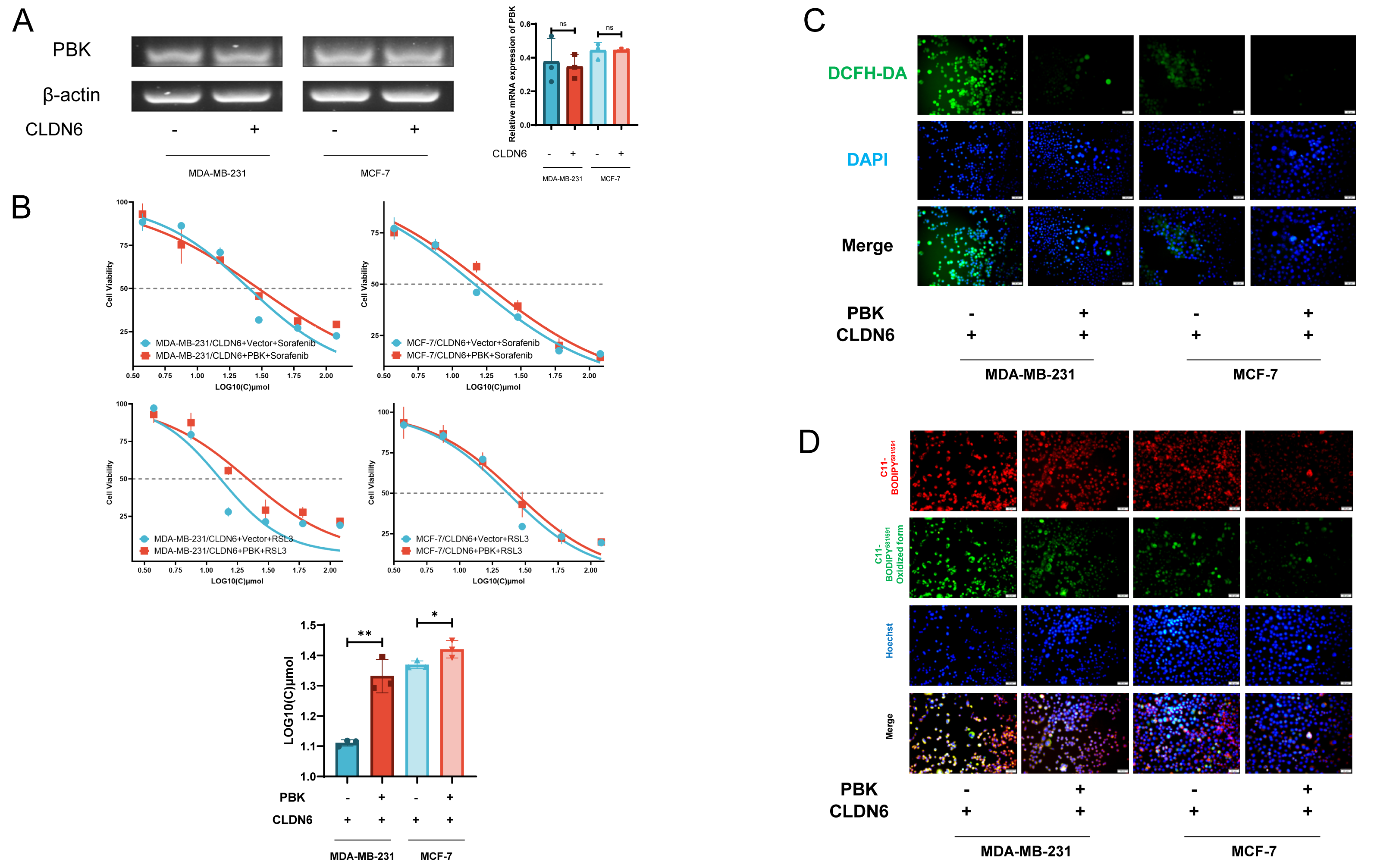


**Fig.S5 CLDN6 regulates the axis AKT/GSK3β/FYN axis through PBK**

**(A)** PBK mRNA levels from the indicated cells were assessed using RT-PCR. **(B)** Cell death was measured and IC50 was calculated by treating the indicated cells with an increased dose of sorafenib and RSL3 for 24 h. **(C)** ROS was measured by DCFH-DA staining, Bar = 50 μm. **(D)** Lipid peroxidation was measured by BODIPY-C11 staining, Blue: Hoechst, Green: BODIPY-C11 oxidized form, Red: BODIPY-C11, Bar = 50 μm. ns, no significance.


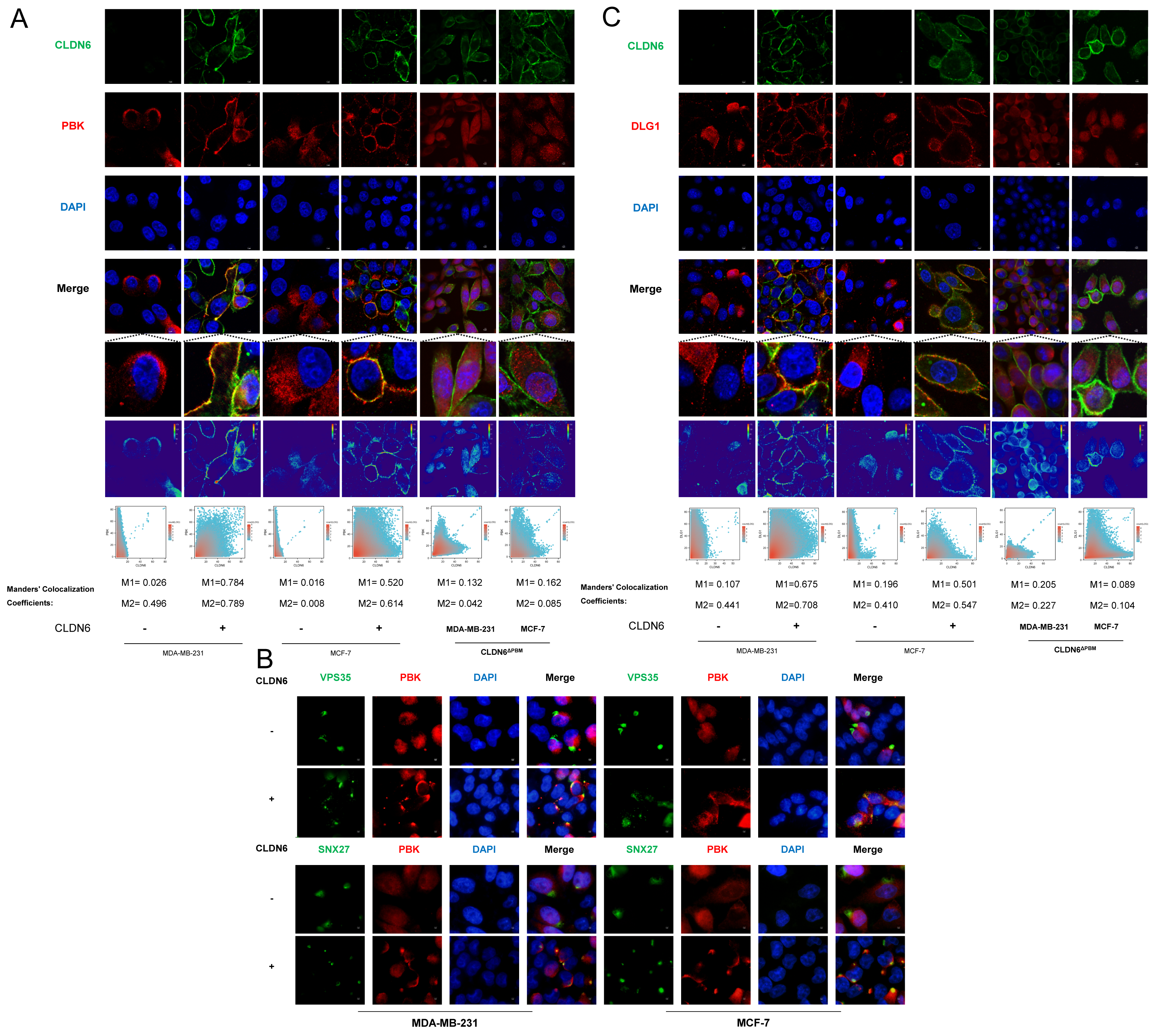


**Fig.S6 The interaction between CLDN6 and the DLG1/PBK complex necessitates the endosomal pathways**

**(A)** and **(C)** The location of PBK, DLG1, and CLDN6 in the indicated cells was observed using fluorescent microscopy. Green: CLDN6; Red: PBK or DLG1; Blue: DAPI. Scale bar: 3 μm. **(B)** The location of VPS35, SNX27, and PBK in the indicated cells was observed using fluorescent microscopy. Green: VPS35 or SNX27; Red: PBK; Blue: DAPI. Scale bar: 3 μm.


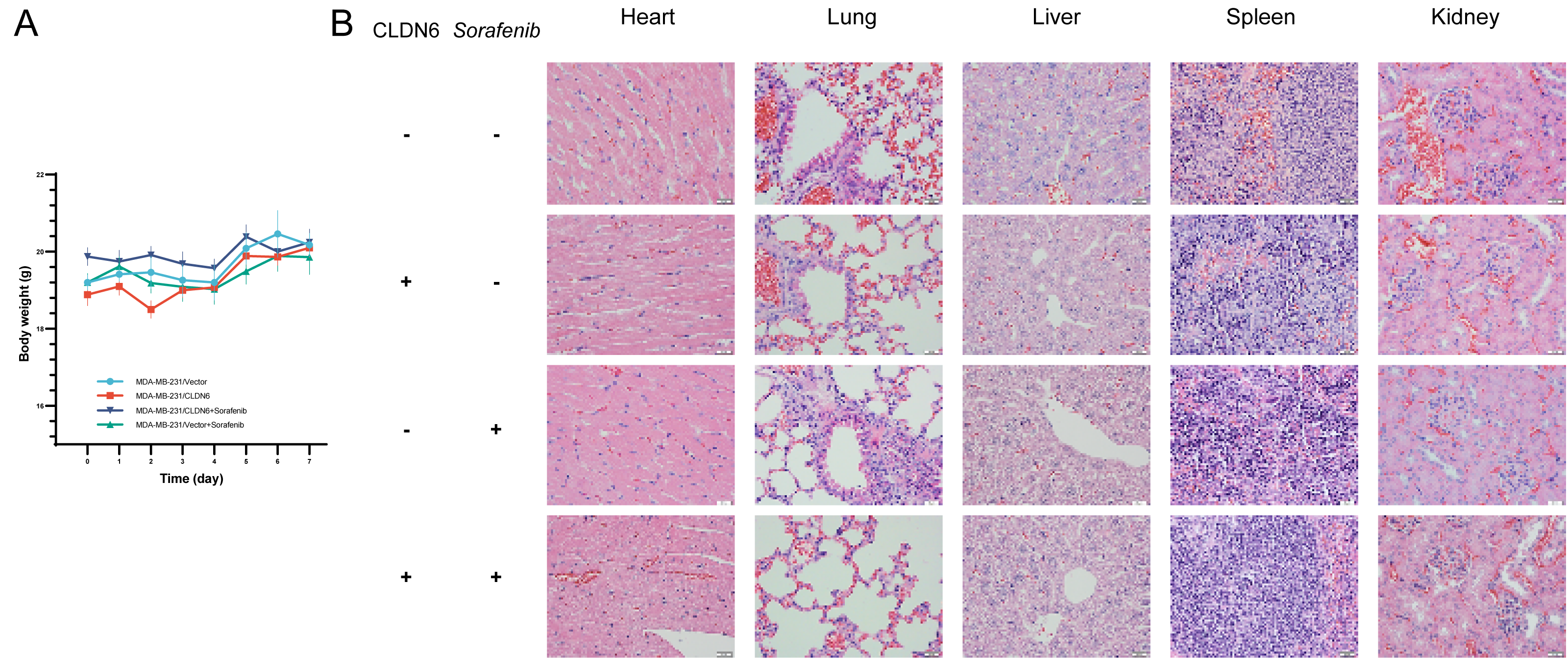


**Fig.S7 CLDN6 triggers breast cancer to undergo ferroptosis in vivo**

**(A)** Changes in body weight of nude mice. **(B)** H&E staining of major organs in nude mice.

**Supplementary Table**

**Table.S1** The first antibodies used in IHC

| Name | Catalog Number | Manufacturer | Dilution |
| --- | --- | --- | --- |
| CLDN6 | sc-393671 | Santa Cruz, USA | 1:100 |
| NRF2 | 16396-1-AP | Proteintech, CN | 1:50 |
| GPX4 | 381958 | Zenbio, CN | 1:100 |

**Table.S2 The primers used in RT-PCR**

| Genes | Primer Sequence |
| --- | --- |
| β-actin | 5′-TCATGAAGTGTGACGTGGACATC-3′ |
|  | 5′-CAGGAGGAGCAATGATCTTGATCT-3′ |
| NRF2 | 5′-TTCAGCCAG CCCAGCACATC-3′ |
|  | 5′-CGTAGCCGAAGAAACCTCATTGTC-3′ |
| PBK | 5′-GAAGAGGACTGAGAGTGGCT-3′ |
|  | 5′-CTTCTGCATAAACGGAGAGGC-3′ |

**Table.S3** The first antibodies used in WB

| Name | Catalog Number | Manufacturer | Dilution |
| --- | --- | --- | --- |
| β-actin | 66009-1-Ig | Proteintech, CN | 1:2000 |
| CLDN6 | sc-393671 | Santa Cruz, USA | 1:500 |
| NRF2 | 16396-1-AP | Proteintech, CN | 1:1000 |
| G6PD | 66373-1-Ig | Proteintech, CN | 1:1000 |
| GPX4 | 381958 | Zenbio, CN | 1:500 |
| H3 | 17168-1-AP | Proteintech, CN | 1:2000 |
| β-tubulin | M20005 | Abmart, CN | 1: 5000 |
| p-AKT | T40067 | Abmart, CN | 1:500 |
| AKT | T55561 | Abmart, CN | 1:500 |
| p-GSK3β | T56568 | Abmart, CN | 1:500 |
| GSK3β | 22104-1-AP | Proteintech, CN | 1:1000 |
| FYN | 66606-1-Ig | Proteintech, CN | 1:5000 |
| PBK | A19947 | ABclonal, CN | 1:500 |
| p-NRF2 | A5203 | Selleck, CN | 1:1000 |
| KEAP1 | 10503-2-AP | Proteintech, CN | 1:2000 |
| DLG1 | T58618 | Abmart, CN | 1:500 |

**Table.S4** The first antibodies used in IF

| Name | Catalog Number | Manufacturer | Dilution |
| --- | --- | --- | --- |
| CLDN6 | sc-393671 | Santa Cruz, USA | 1:100 |
| PBK | A19947 | ABclonal, CN | 1:50 |
| DLG1 | T58618 | Abmart, CN | 1:50 |
| VPS35 | sc-374372 | Santa Cruz, USA | 1:100 |
| SNX27 | sc-515707 | Santa Cruz, USA | 1:100 |

**Table.S5 The Meta-cohort of breast** cancer and normal tissue

| **Tissue** | **Dataset ID** |
| --- | --- |
| **Breast** | GSE11121 |
|  | GSE12237 |
|  | GSE1456 |
|  | GSE15852 |
|  | GSE16873 |
|  | GSE2034 |
|  | GSE22093 |
|  | GSE2361 |
|  | GSE23988 |
|  | GSE24185 |
|  | GSE24509 |
|  | GSE25066 |
|  | GSE2603 |
|  | GSE31519 |
|  | GSE32072 |
|  | GSE3494 |
|  | GSE36774 |
|  | GSE45255 |
|  | GSE4611 |
|  | GSE46184 |
|  | GSE48984 |
|  | GSE5327 |
|  | GSE5847 |
|  | GSE6532 |
|  | GSE6772 |
|  | GSE68892 |
|  | GSE83232 |
|  | GSE92697 |
|  | GSE9574 |
|  | GSE9662 |
|  | GSE4922 |
|  | GSE5364 |
|  | GSE7390 |
|  | GSE5462 |
|  | GSE12093 |
|  | GSE3726 |
|  | GSE1561 |
|  | GSE6883 |
|  | GSE11965 |
|  | GSE12630 |

**Table.S6 The Meta-cohort of tumor** and normal tissue

| **Tissue** | **Dataset ID** |
| --- | --- |
| **Adrenal Gland** | GSE75415 |
|  | GSE68606 |
|  | GSE76021 |
| **Bladder** | GSE2361 |
|  | GSE3167 |
|  | GSE12630 |
| **Blood** | GSE11907 |
|  | GSE6477 |
|  | GSE1133 |
|  | GSE11582 |
|  | GSE8650 |
|  | GSE9006 |
|  | GSE6269 |
|  | GSE7638 |
|  | GSE3365 |
|  | GSE6613 |
|  | GSE1466 |
|  | GSE6236 |
|  | GSE9476 |
|  | GSE9874 |
|  | GSE10631 |
|  | GSE5580 |
|  | GSE6740 |
|  | GSE1751 |
|  | GSE2779 |
|  | GSE1010 |
|  | GSE7893 |
|  | GSE5967 |
|  | GSE7429 |
|  | GSE1124 |
|  | GSE1140 |
|  | GSE5808 |
|  | GSE7148 |
|  | GSE12845 |
|  | GSE14577 |
|  | E-AFMX-5 |
|  | GSE13591 |
|  | GSE15777 |
|  | GSE14317 |
|  | GSE6401 |
|  | GSE4475 |
|  | GSE635 |
|  | GSE1427 |
|  | GSE12995 |
|  | GSE10255 |
|  | GSE2351 |
|  | GSE3912 |
|  | GSE4119 |
|  | GSE6365 |
|  | GSE11038 |
|  | GSE2113 |
|  | GSE4698 |
|  | GSE5122 |
|  | GSE6691 |
|  | GSE13280 |
|  | GSE8970 |
|  | GSE5820 |
|  | GSE5788 |
|  | GSE14286 |
|  | GSE13996 |
|  | E-MEXP-120 |
|  | E-MEXP-313 |
|  | E-TABM-117 |
|  | E-TABM-125 |
| **Bone Marrow** | GSE16102 |
|  | GSE16334 |
|  | GSE18026 |
|  | GSE19147 |
|  | GSE22529 |
|  | GSE28497 |
|  | GSE43176 |
|  | GSE68954 |
|  | GSE8835 |
|  | GSE10139 |
|  | GSE10172 |
|  | GSE1159 |
|  | GSE12417 |
|  | GSE13425 |
|  | GSE14618 |
|  | GSE15347 |
|  | GSE1577 |
|  | GSE16131 |
|  | GSE16746 |
|  | GSE17195 |
|  | GSE1729 |
|  | GSE34171 |
|  | GSE34860 |
|  | GSE37088 |
|  | GSE37642 |
|  | GSE44164 |
|  | GSE48184 |
|  | GSE51082 |
|  | GSE647 |
|  | GSE649 |
|  | GSE660 |
|  | GSE67684 |
|  | GSE78132 |
|  | GSE83449 |
|  | GSE8510 |
|  | GSE8879 |
|  | GSE9429 |
| **Brain** | GSE12907 |
|  | GSE13471 |
|  | GSE20186 |
|  | GSE20295 |
|  | GSE2175 |
|  | GSE24250 |
|  | GSE5390 |
|  | GSE5392 |
|  | GSE62600 |
|  | GSE6306 |
|  | GSE3790 |
|  | GSE12649 |
|  | GSE9963 |
|  | GSE2719 |
|  | GSE1147 |
|  | GSE9335 |
|  | GSE12685 |
|  | E-MEXP-114 |
|  | E-MEXP-1690 |
|  | GSE24072 |
|  | GSE2485 |
|  | GSE3185 |
|  | GSE4271 |
|  | GSE83294 |
|  | GSE8692 |
|  | GSE13041 |
|  | GSE4412 |
|  | GSE2841 |
|  | GSE1993 |
|  | GSE4780 |
| **Breast** | GSE15852 |
|  | GSE16873 |
|  | GSE48984 |
|  | GSE6772 |
|  | GSE9574 |
|  | GSE5364 |
|  | GSE6883 |
|  | GSE11121 |
|  | GSE12237 |
|  | GSE1456 |
|  | GSE2034 |
|  | GSE22093 |
|  | GSE23988 |
|  | GSE24185 |
|  | GSE24509 |
|  | GSE25066 |
|  | GSE2603 |
|  | GSE31519 |
|  | GSE32072 |
|  | GSE3494 |
|  | GSE36774 |
|  | GSE45255 |
|  | GSE4611 |
|  | GSE46184 |
|  | GSE5327 |
|  | GSE5847 |
|  | GSE6532 |
|  | GSE68892 |
|  | GSE83232 |
|  | GSE92697 |
|  | GSE9662 |
|  | GSE4922 |
|  | GSE7390 |
|  | GSE5462 |
|  | GSE12093 |
|  | GSE3726 |
|  | GSE1561 |
|  | GSE11965 |
| **Cervix** | GSE7803 |
| **Colon** | GSE24514 |
|  | GSE41258 |
|  | GSE62322 |
|  | GSE77955 |
|  | GSE6272 |
|  | GSE1152 |
|  | E-MTAB-57 |
|  | GSE12945 |
|  | GSE26682 |
|  | GSE4045 |
|  | GSE68468 |
|  | GSE7208 |
|  | GSE2138 |
|  | E-MEXP-383 |
|  | E-MEXP-833 |
| **Esophagus** | GSE1420 |
|  | GSE23400 |
|  | GSE13083 |
|  | GSE37203 |
| **Kidney** | GSE15641 |
|  | GSE2004 |
|  | GSE27556 |
|  | GSE6280 |
|  | GSE6344 |
|  | GSE65162 |
|  | GSE781 |
|  | GSE3297 |
|  | E-TABM-53 |
|  | GSE10320 |
|  | GSE11482 |
|  | GSE14767 |
|  | GSE30946 |
|  | GSE31403 |
|  | GSE2712 |
|  | GSE11904 |
| **Liver** | GSE19281 |
|  | GSE60502 |
|  | GSE7473 |
|  | GSE14323 |
|  | E-TABM-36 |
|  | E-TABM-292 |
| **Lung** | GSE10072 |
|  | GSE19027 |
|  | GSE31908 |
|  | GSE39262 |
|  | GSE40839 |
|  | GSE68465 |
|  | GSE75324 |
|  | GSE7670 |
|  | GSE5060 |
|  | GSE7895 |
|  | GSE994 |
|  | GSE2549 |
|  | GSE2395 |
|  | GSE1650 |
|  | E-MEXP-231 |
|  | E-TABM-15 |
|  | GSE17475 |
|  | GSE6253 |
|  | GSE9971 |
|  | GSE3593 |
|  | GSE4573 |
| **Ovary** | GSE26712 |
|  | GSE34405 |
|  | GSE6008 |
|  | GSE14764 |
|  | GSE23603 |
|  | GSE28015 |
|  | GSE3149 |
| **Pancreas** | GSE43288 |
| **Pharynx** | GSE13597 |
| **Prostate** | GSE12348 |
|  | GSE8218 |
|  | E-MEXP-1327 |
|  | E-TABM-26 |
|  | GSE2443 |
|  | GSE25136 |
| **Skin** | GSE1317 |
|  | GSE46517 |
|  | GSE9118 |
|  | GSE4845 |
|  | GSE3189 |
|  | GSE8440 |
|  | GSE5667 |
|  | GSE6012 |
|  | GSE2503 |
|  | GSE8401 |
|  | GSE9782 |
|  | GSE12627 |
| **Soft Tissue** | GSE21124 |
| **Stomach** | GSE29272 |
|  | GSE37023 |
|  | GSE15460 |
| **Testis** | GSE3218 |
|  | GSE10783 |
|  | GSE10615 |
| **Thyroid** | GSE27155 |
|  | GSE5054 |
| **Tongue** | GSE31853 |
|  | GSE3524 |
| **Uterus** | GSE36389 |
|  | GSE9750 |
|  | GSE2152 |
|  | GSE11855 |

**Table.S7 Ferroptosis-related gene** set for GSVA analysis

| **Set** | **Gene** |
| --- | --- |
| Ferroptosis promoting gene | ACSL1, ACSL4, ACSL6, ALOX15, ATG5, ATG7, FTL, LPCAT3, MAP1LC3A, MAP1LC3B, NCOA4, SAT1, SLC39A14, TF, TFRC, IFNG, KEAP1, PHKG2, SOCS1, EGFR, WWTR1, CDKN2A, ALOX15B, ALOX12, ATF3, ALOX5, BAP1, SIRT3, KLF2, CDO1, STING1, IL6, FADS1, TRIM21, PTGS2, BRD4, TMEM164 |
| Ferroptosis suppressing gene | ACSL3, GCLC, GCLM, GPX4, GSS, SLC3A2, SLC40A1, SLC7A11, PROM2, CDKN1A, LAMP2, G6PD, NFE2L2, MTOR, CDH1, SQSTM1, PRDX6, NUPR1, NFS1, GOT1, RB1, NFE2L1, NEDD4, CPT1A, PIK3CA, CLOCK, HSPA8, MARCHF6, BRCA1, METTL16, HMGCR, REST, TXNRD1, DECR1, DHODH |

**Table.S8** Univariate and multivariate Cox regression analysis of TCGA

| Characteristics | | Total(N) | Univariate analysis | *P* value | Multivariate analysis | | *P* value |  |
| --- | --- | --- | --- | --- | --- | --- | --- | --- |
|  |  |  | Hazard ratio (95% CI) |  | Hazard ratio (95% CI) | |  |  |
|  | AGE | 1061 | 1.034 (1.021 - 1.047) | **< 0.001** | | 1.036 (1.020 - 1.052) | **< 0.001** | |
|  | SUBTYPE | 974 |  |  | |  |  | |
|  | Normal | 36 | Reference |  | | Reference |  | |
|  | LumA | 493 | 0.579 (0.263 - 1.274) | 0.174 | | 0.472 (0.181 - 1.230) | 0.125 | |
|  | LumB | 196 | 0.994 (0.436 - 2.264) | 0.989 | | 0.555 (0.203 - 1.521) | 0.253 | |
|  | Her2 | 78 | 1.263 (0.514 - 3.100) | 0.611 | | 1.142 (0.388 - 3.361) | 0.809 | |
|  | Basal | 171 | 0.627 (0.267 - 1.474) | 0.285 | | 1.058 (0.379 - 2.952) | 0.915 | |
|  | Pathological Stage | 1042 |  |  | |  |  | |
|  | I | 178 | Reference |  | | Reference |  | |
|  | II | 605 | 1.598 (0.925 - 2.762) | 0.093 | | 1.077 (0.405 - 2.867) | 0.882 | |
|  | III | 242 | 2.990 (1.684 - 5.309) | **< 0.001** | | 2.585 (0.668 - 10.008) | 0.169 | |
|  | IV | 17 | 12.276 (5.883 - 25.617) | **< 0.001** | | 4.952 (0.587 - 41.790) | 0.142 | |
|  | M Stage | 905 |  |  | |  |  | |
|  | M0 | 886 | Reference |  | | Reference |  | |
|  | M1 | 19 | 4.494 (2.602 - 7.761) | **< 0.001** | | 1.251 (0.268 - 5.843) | 0.776 | |
|  | N Stage | 1042 |  |  | |  |  | |
|  | N0 | 506 | Reference |  | | Reference |  | |
|  | N1 | 346 | 1.829 (1.234 - 2.711) | **0.003** | | 1.452 (0.817 - 2.580) | 0.203 | |
|  | N2 | 115 | 2.507 (1.475 - 4.262) | **< 0.001** | | 1.470 (0.531 - 4.066) | 0.458 | |
|  | N3 | 75 | 4.166 (2.303 - 7.537) | **< 0.001** | | 1.496 (0.521 - 4.292) | 0.454 | |
|  | T Stage | 1058 |  |  | |  |  | |
|  | T1 | 273 | Reference |  | | Reference |  | |
|  | T2 | 613 | 1.268 (0.842 - 1.911) | 0.256 | | 0.985 (0.466 - 2.080) | 0.968 | |
|  | T3 | 137 | 1.559 (0.925 - 2.626) | 0.095 | | 0.725 (0.277 - 1.894) | 0.511 | |
|  | T4 | 35 | 3.719 (1.938 - 7.138) | **< 0.001** | | 1.062 (0.352 - 3.207) | 0.915 | |
|  | C&F classifier | 1061 |  |  | |  |  | |
|  | High&High | 218 | Reference |  | | Reference |  | |
|  | High&Low | 52 | 2.388 (1.086 - 5.250) | **0.030** | | 4.434 (1.732 - 11.353) | **0.002** | |
|  | Low&High | 532 | 1.318 (0.802 - 2.167) | 0.275 | | 1.456 (0.754 - 2.811) | 0.263 | |
|  | Low&Low | 259 | 2.140 (1.267 - 3.614) | **0.004** | | 2.268 (1.087 - 4.730) | **0.029** | |

**Table.S9** Univariate and multivariate Cox regression analysis of TMA

| Characteristics | Total(N) | | Univariate analysis | *P* value | Multivariate analysis | | *P* value | |
| --- | --- | --- | --- | --- | --- | --- | --- | --- |
|  |  |  | HR(95% CI) |  | HR(95% CI) | |  |  |
| Pathological grade | 68 |  | |  |  |  | |  |
| 1~2 | 7 | Reference | |  |  |  | |  |
| 2~3 | 61 | 0.542 (0.157 - 1.872) | | 0.332 |  |  | |  |
| Stage | 56 |  | |  |  |  | |  |
| StageⅠ | 13 | Reference | |  |  |  | |  |
| StageⅡ | 24 | 1.215 (0.304 - 4.860) | | 0.783 |  |  | |  |
| StageⅢ | 19 | 2.123 (0.562 - 8.011) | | 0.267 |  |  | |  |
| Stage Ⅳ | 0 |  | |  |  |  | |  |
| T | 61 |  | |  |  |  | |  |
| T1 | 22 | Reference | |  |  |  | |  |
| T2 | 35 | 1.527 (0.531 - 4.398) | | 0.432 |  |  | |  |
| T3~4 | 4 | 1.163 (0.136 - 9.958) | | 0.890 |  |  | |  |
| N | 62 |  | |  |  |  | |  |
| N0 | 29 | Reference | |  | Reference |  | |  |
| N1 | 14 | 3.451 (0.926 - 12.871) | | 0.065 | 4.285 (1.091 - 16.827) | **0.037** | |  |
| N2 | 14 | 2.257 (0.564 - 9.029) | | 0.250 | 1.800 (0.442 - 7.320) | 0.412 | |  |
| N3 | 5 | 10.267 (2.503 - 42.125) | | **0.001** | 8.101 (1.849 - 35.488) | **0.006** | |  |
| ER | 71 |  | |  |  |  | |  |
| - | 19 | Reference | |  |  |  | |  |
| + | 52 | 0.943 (0.336 - 2.646) | | 0.912 |  |  | |  |
| PR | 69 |  | |  |  |  | |  |
| - | 30 | Reference | |  |  |  | |  |
| + | 39 | 0.581 (0.229 - 1.472) | | 0.252 |  |  | |  |
| HER2 | 73 |  | |  |  |  | |  |
| - | 37 | Reference | |  |  |  | |  |
| + | 36 | 0.573 (0.226 - 1.456) | | 0.242 |  |  | |  |
| KI67 | 69 |  | |  |  |  | |  |
| - | 15 | Reference | |  |  |  | |  |
| + | 54 | 0.478 (0.179 - 1.274) | | 0.140 |  |  | |  |
| KI67% | 72 |  | |  |  |  | |  |
| <20% | 25 | Reference | |  |  |  | |  |
| 20~30% | 12 | 0.470 (0.100 - 2.212) | | 0.339 |  |  | |  |
| 30~40% | 16 | 0.980 (0.321 - 2.998) | | 0.972 |  |  | |  |
| 40~50% | 7 | 0.435 (0.054 - 3.483) | | 0.433 |  |  | |  |
| 50~60% | 6 | 0.489 (0.061 - 3.912) | | 0.500 |  |  | |  |
| >60% | 6 | 0.985 (0.209 - 4.640) | | 0.984 |  |  | |  |
| CLDN6&Ferroptosis | 75 |  | |  |  |  | |  |
| High&High | 24 | Reference | |  | Reference |  | |  |
| High&Low | 17 | 2.279 (0.381 - 13.640) | | 0.367 | 1.185 (0.193 - 7.297) | 0.855 | |  |
| Low&High | 23 | 2.937 (0.570 - 15.142) | | 0.198 | 2.511 (0.480 - 13.145) | 0.276 | |  |
| Low&Low | 11 | 20.896 (4.507 - 96.890) | | **< 0.001** | 11.620 (2.287 - 59.043) | **0.003** | |  |

**References**

1. Park SJ, et al. GENT2: an updated gene expression database for normal and tumor tissues. BMC medical genomics. 2019;12(Suppl 5):101.

2. Hu C, et al. CellMarker 2.0: an updated database of manually curated cell markers in human/mouse and web tools based on scRNA-seq data. Nucleic acids research. 2023;51(D1):D870-d6.

3. Langfelder P, Horvath S. WGCNA: an R package for weighted correlation network analysis. BMC Bioinformatics. 2008;9:559.

4. Maeser D, et al. oncoPredict: an R package for predicting in vivo or cancer patient drug response and biomarkers from cell line screening data. Brief Bioinform. 2021;22(6).

5. Yang M, et al. CLDN6 promotes chemoresistance through GSTP1 in human breast cancer. Journal of experimental & clinical cancer research : CR. 2017;36(1):157.

6. Qu H, et al. CLDN6 Suppresses c-MYC-Mediated Aerobic Glycolysis to Inhibit Proliferation by TAZ in Breast Cancer. International journal of molecular sciences. 2021;23(1).

7. Jia YY, et al. A SUMOylation-dependent HIF-1 alpha/CLDN6 negative feedback mitigates hypoxia-induced breast cancer metastasis. J Exp Clin Cancer Res. 2020;39(1):15.
