# Supplementary material for: CLDN6 triggers NRF2-mediated ferroptosis through recruiting DLG1/PBK complex in breast cancer
Source: Cell Death Dis. 2025 Feb 21;16(1):122. doi: 10.1038/s41419-025-07448-9 (PMC11845765; doi:10.1038/s41419-025-07448-9)
Supplement: Supplementary file 2 — Original western blot data [file 41419_2025_7448_MOESM2_ESM.pdf]

Fig. 3 C

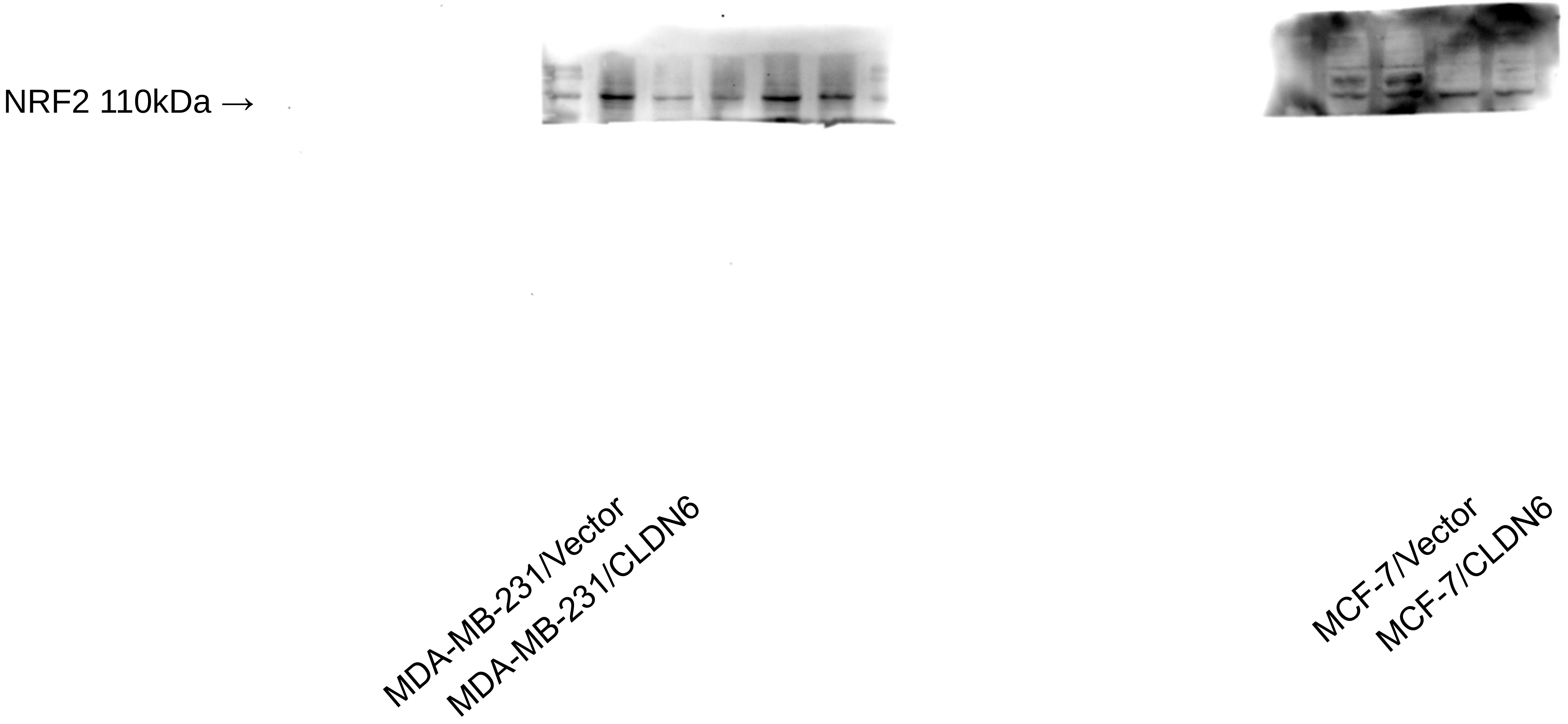
NRF2 110kDa →
MDA-MB-231/Vector
MDA-MB-231/CLDN6
MCF-7/Vector
MCF-7/CLDN6

Fig. 3 C

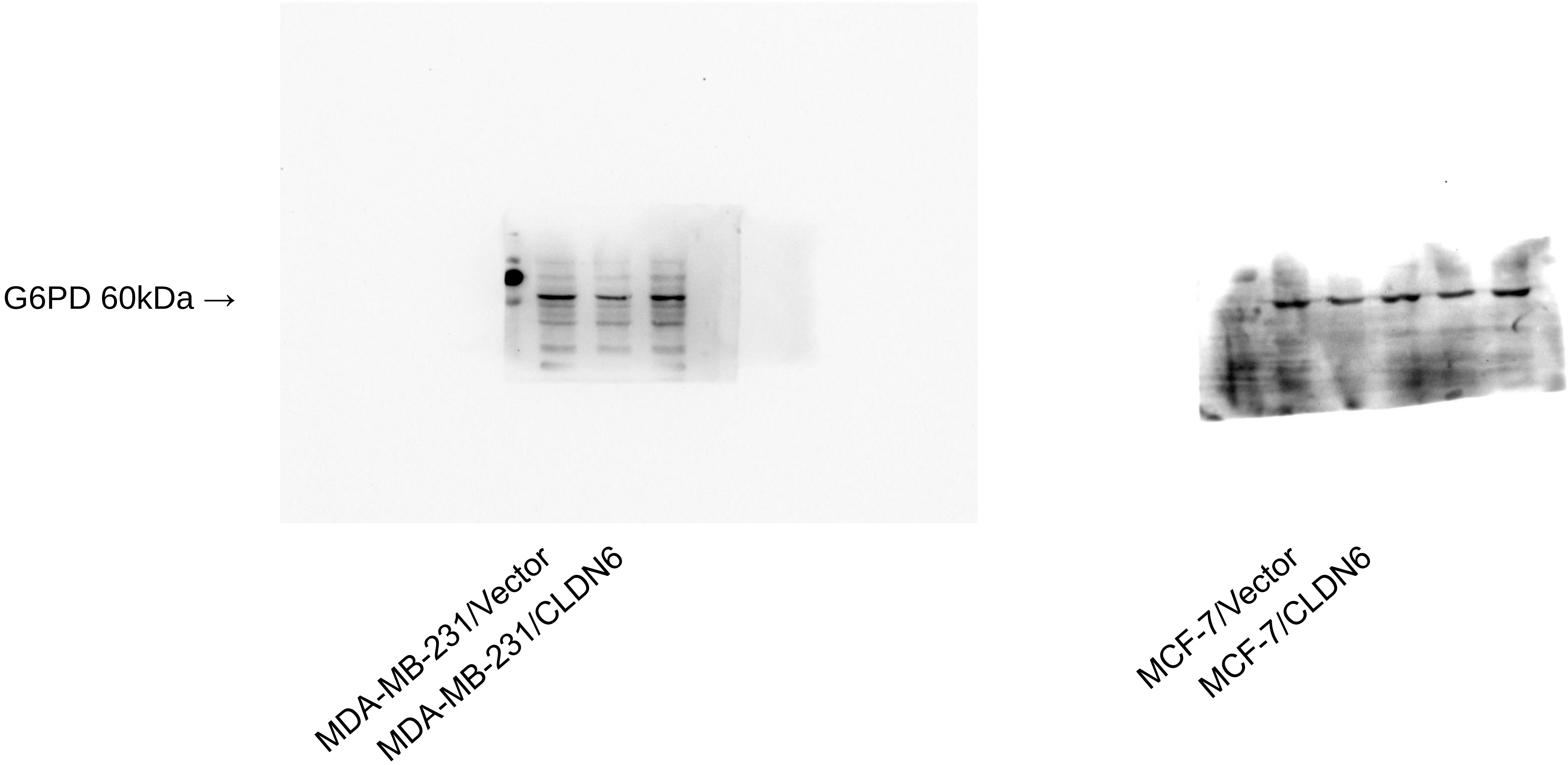
G6PD 60kDa →
MDA-MB-231/Vector
MDA-MB-231/CLDN6
MCF-7/Vector
MCF-7/CLDN6

Fig. 3 C

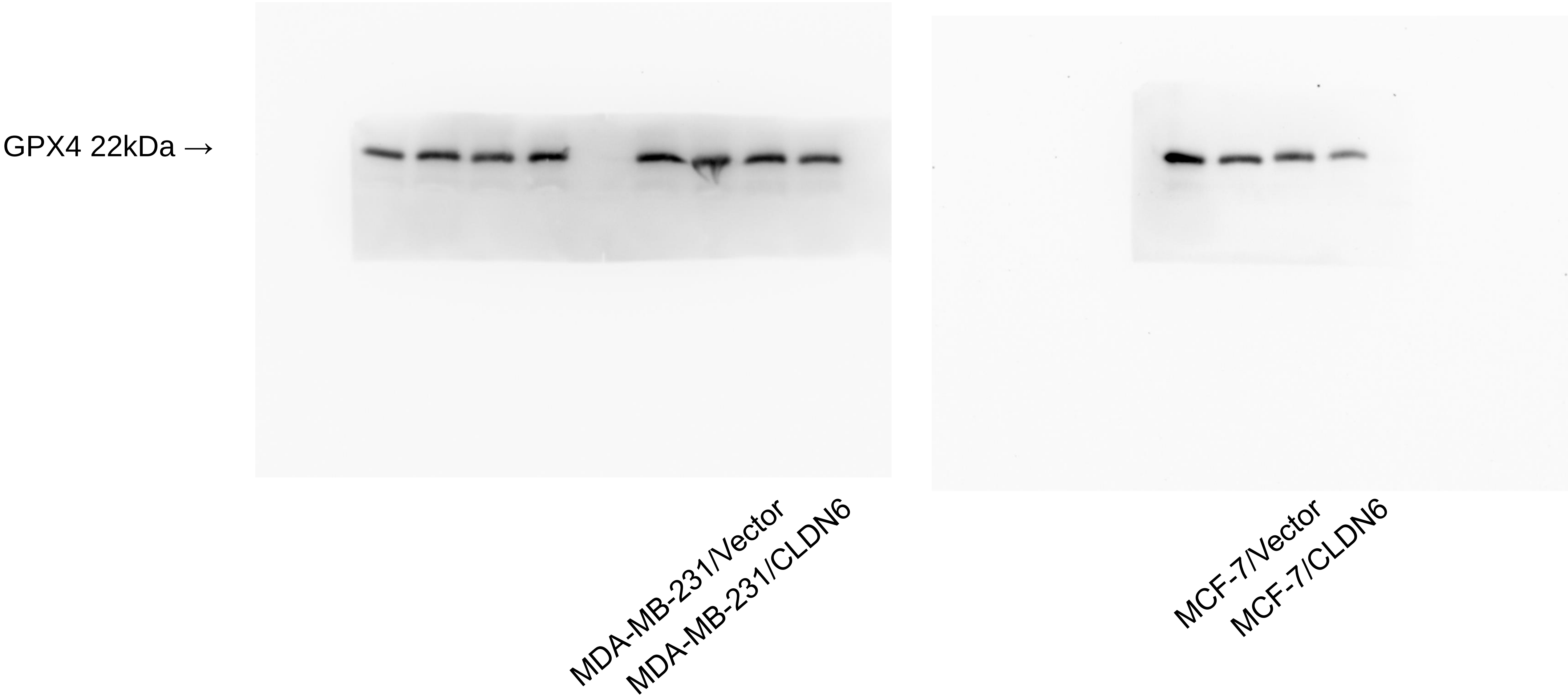
GPX4 22kDa →
MDA-MB-231/Vector
MDA-MB-231/CLDN6
MCF-7/Vector
MCF-7/CLDN6

Fig. 3 C

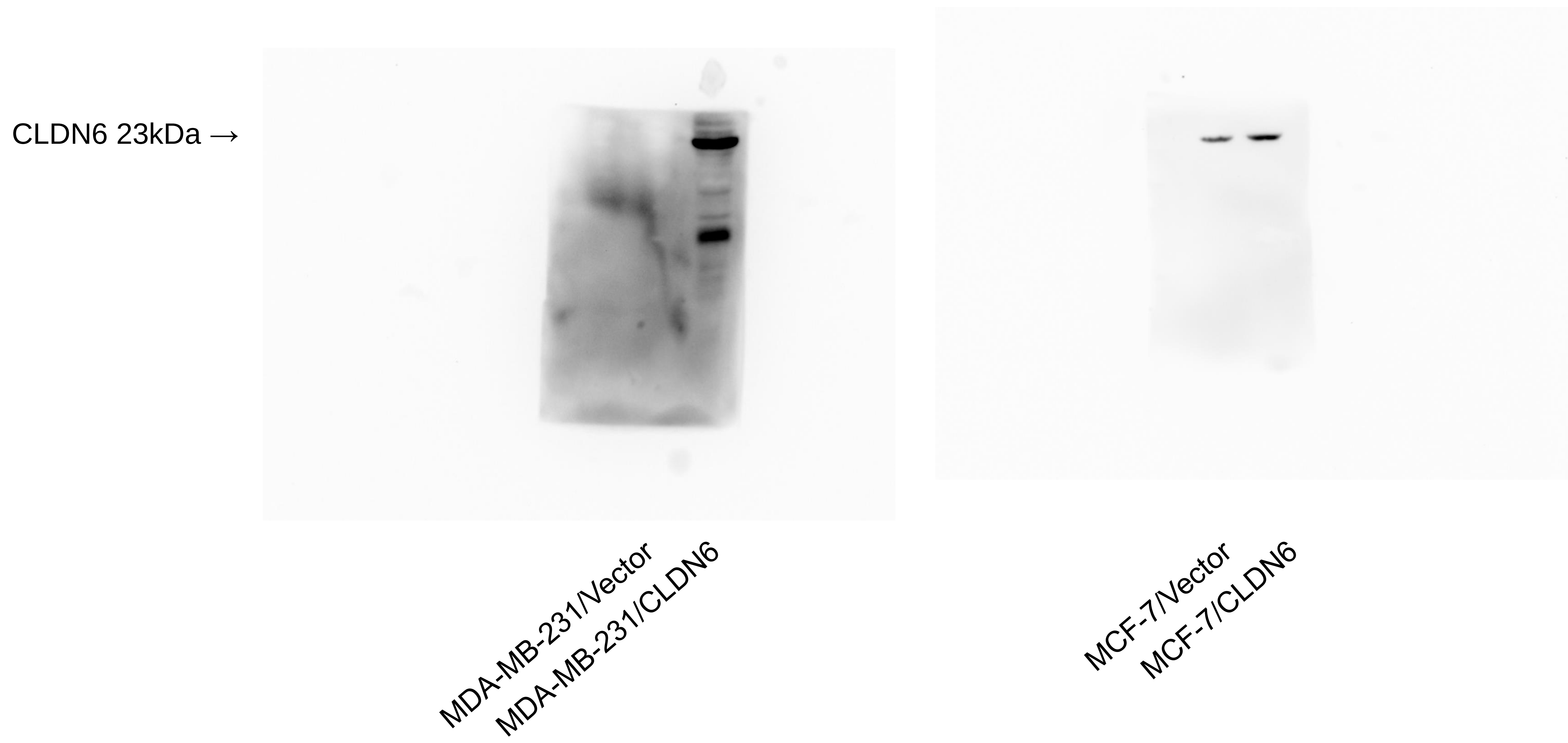

CLDN6 23kDa →
MDA-MB-231/Vector
MDA-MB-231/CLDN6
MCF-7/Vector
MCF-7/CLDN6

Fig. 3 C

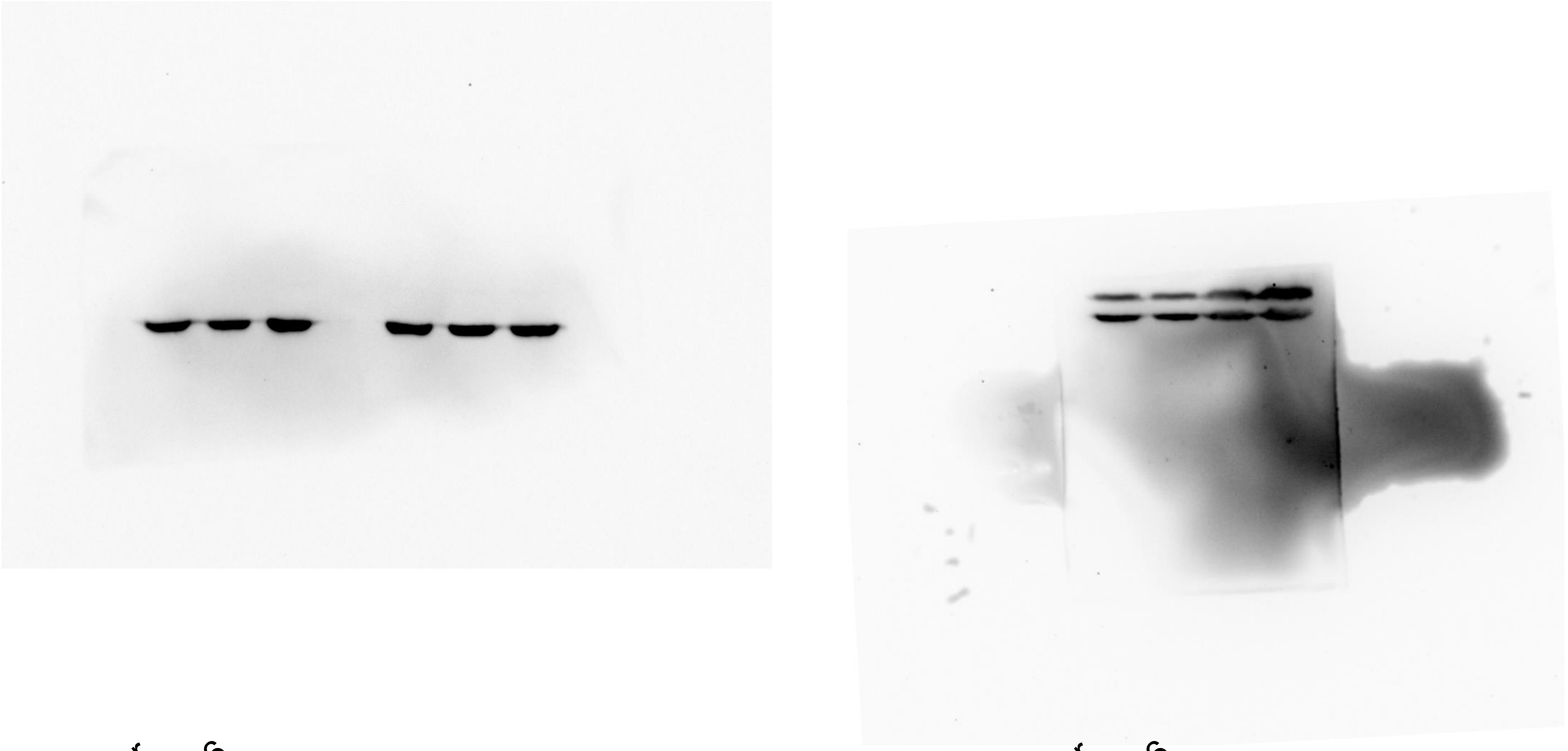
β-actin 42kDa →
MDA-MB-231/Vector
MDA-MB-231/CLDN6
MCF-7/Vector
MCF-7/CLDN6

Fig. 3 D

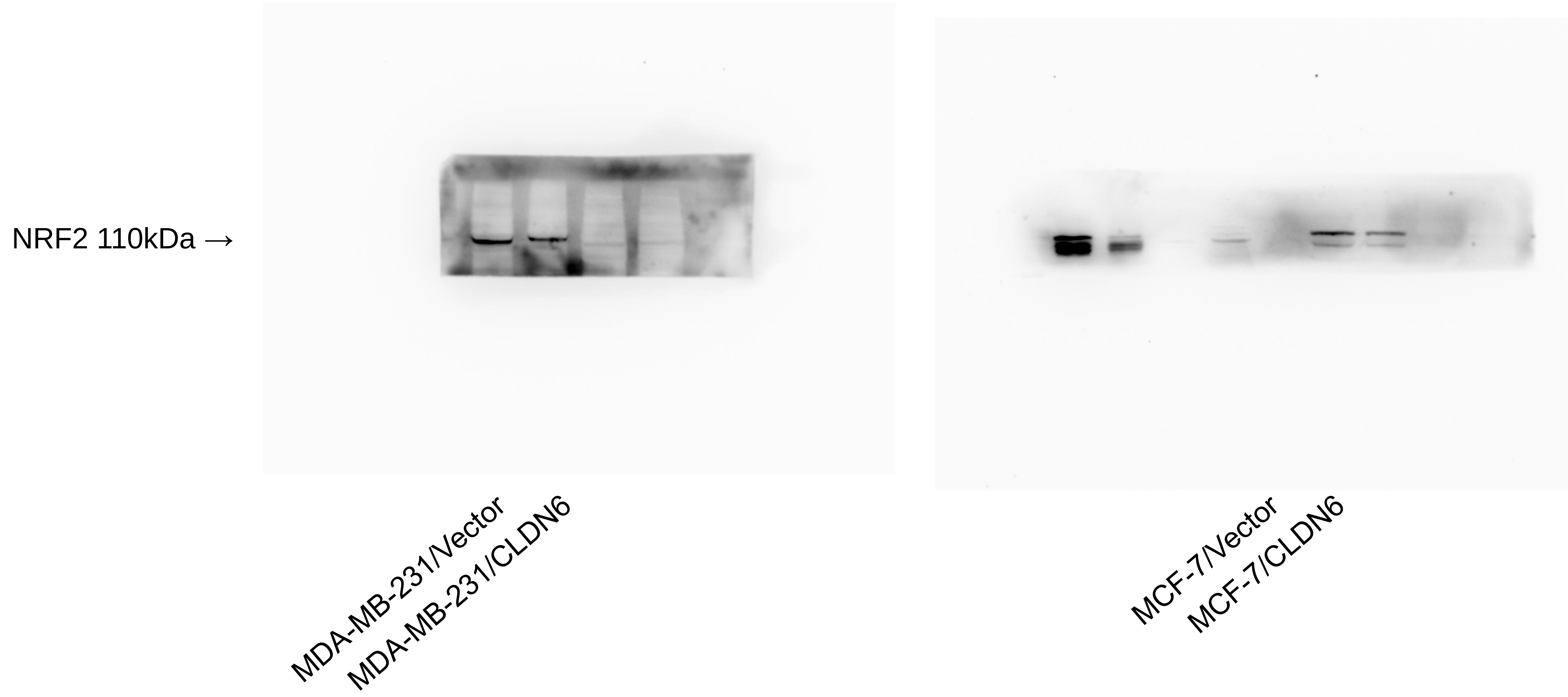
NRF2 110kDa →
MDA-MB-231/Vector
MDA-MB-231/CLDN6
MCF-7/Vector
MCF-7/CLDN6

Fig. 3 D

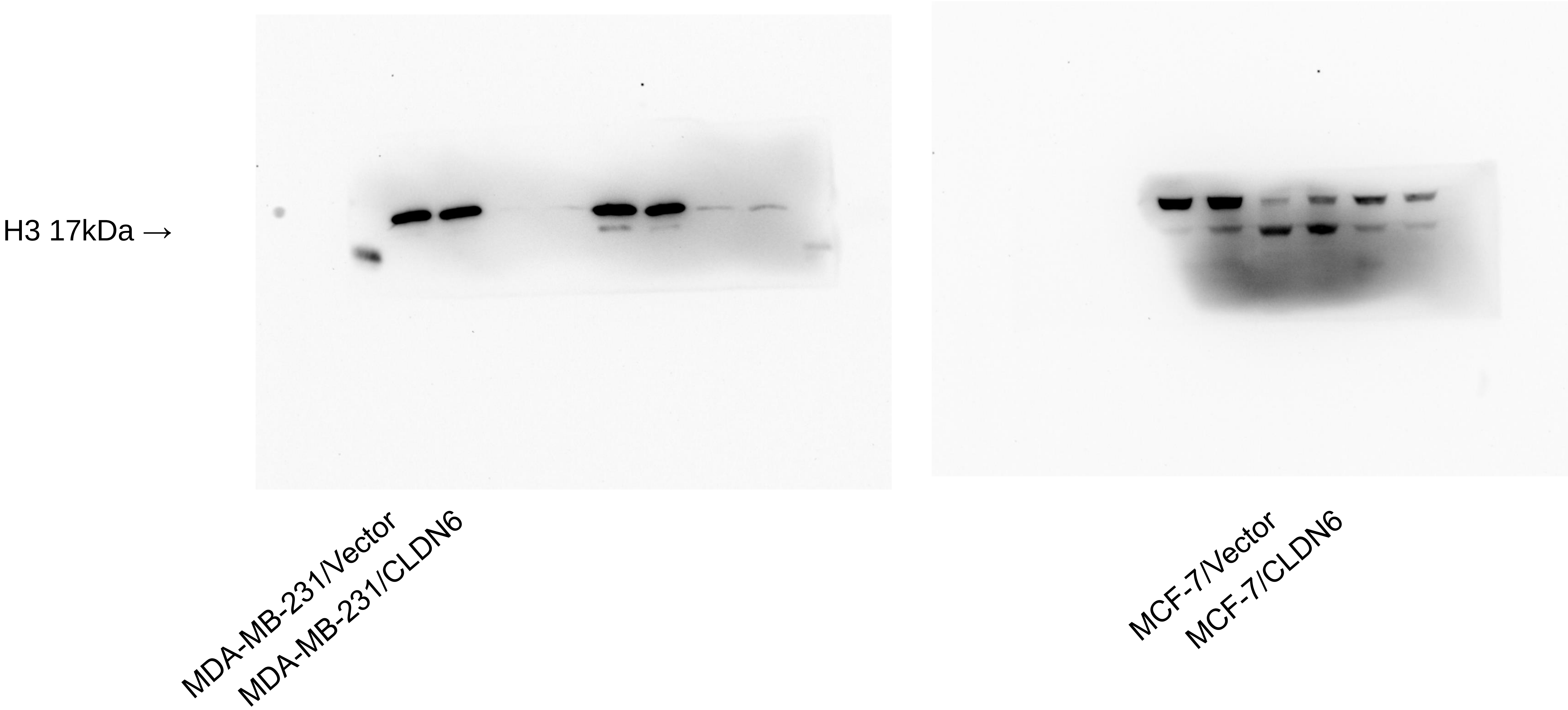
H3 17kDa →
MDA-MB-231/Vector
MDA-MB-231/CLDN6
MCF-7/Vector
MCF-7/CLDN6

Fig. 3 D

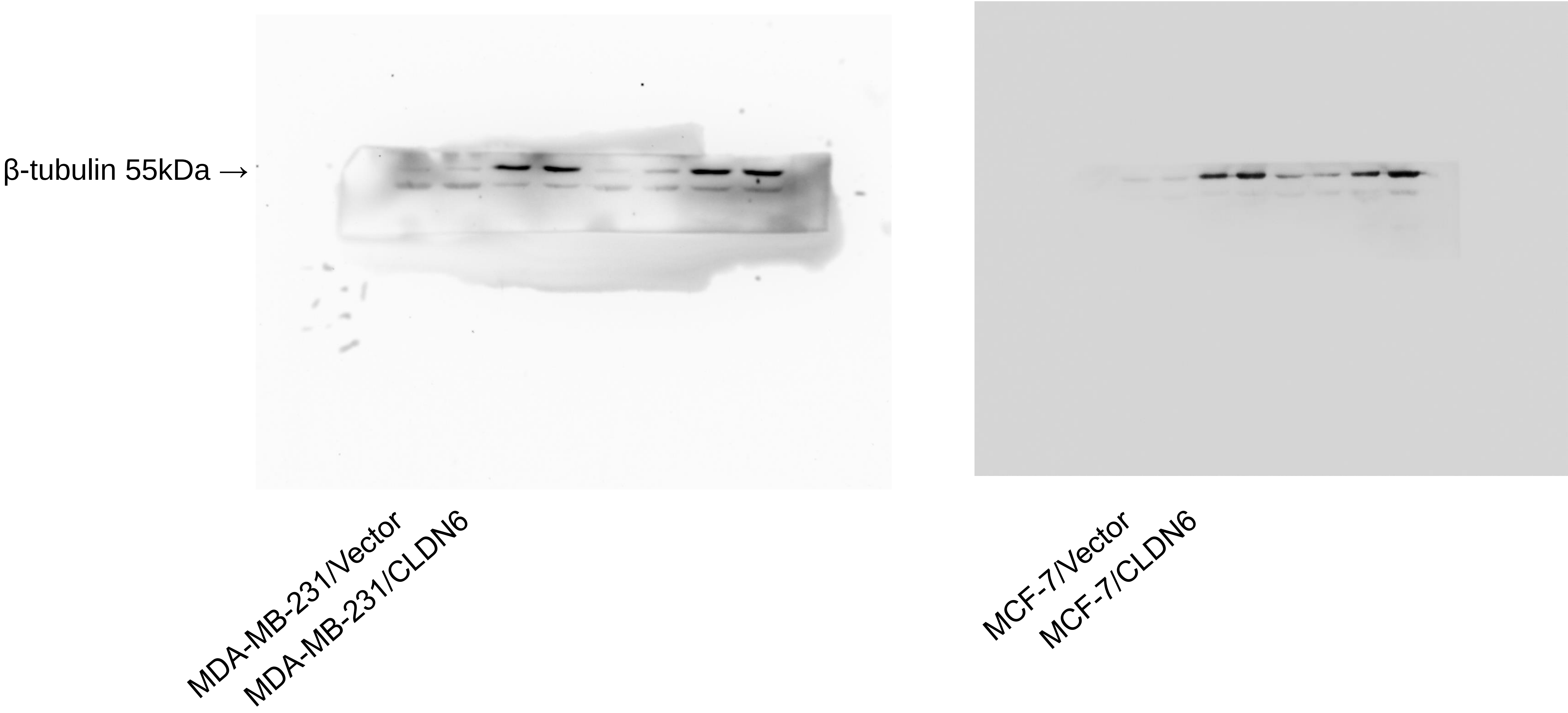
β-tubulin 55kDa →
MDA-MB-231/Vector
MDA-MB-231/CLDN6
MCF-7/Vector
MCF-7/CLDN6

Fig. 3 F

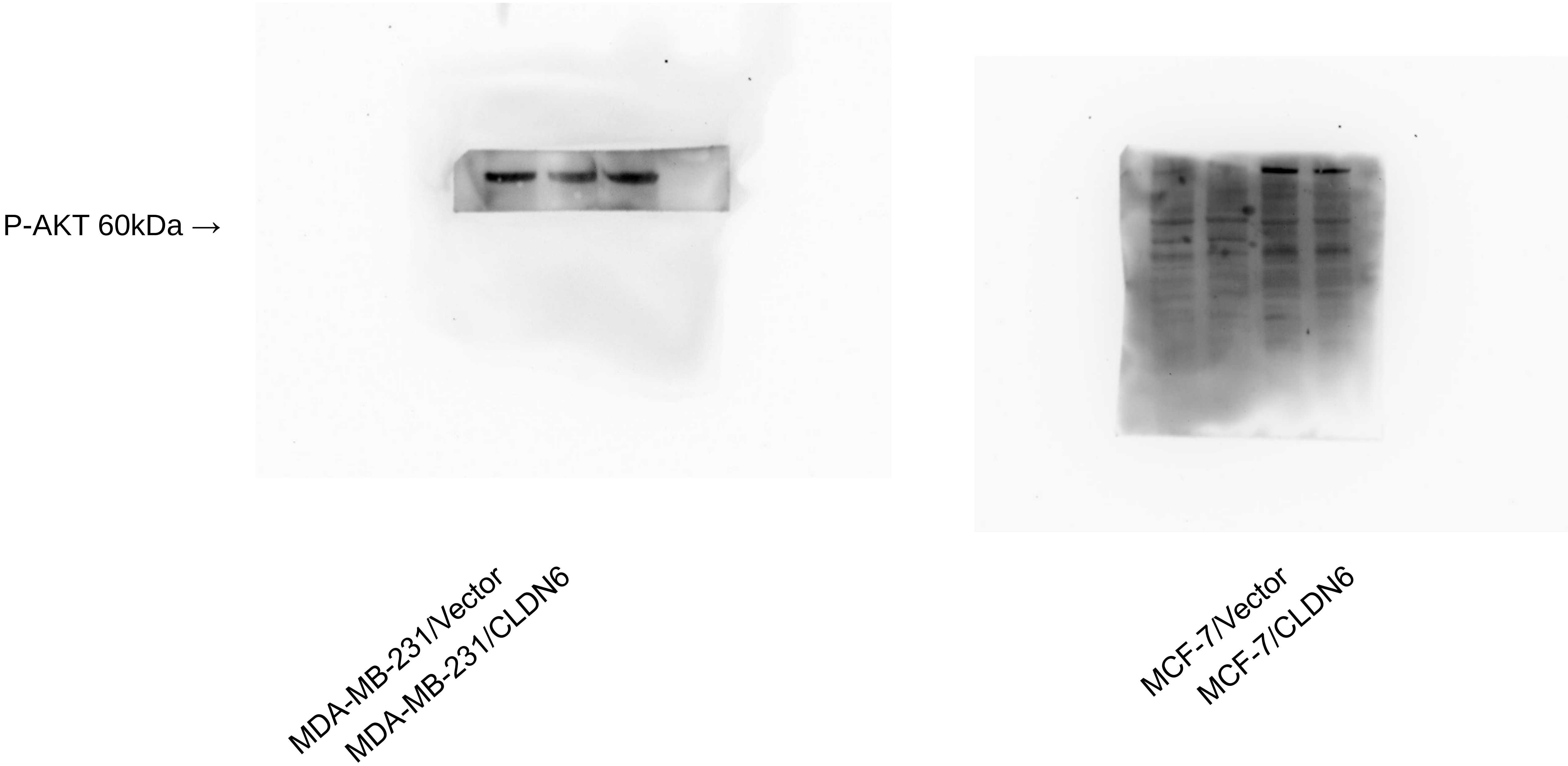

P-AKT 60kDa →
MDA-MB-231/Vector
MDA-MB-231/CLDN6
MCF-7/Vector
MCF-7/CLDN6

Fig. 3 F

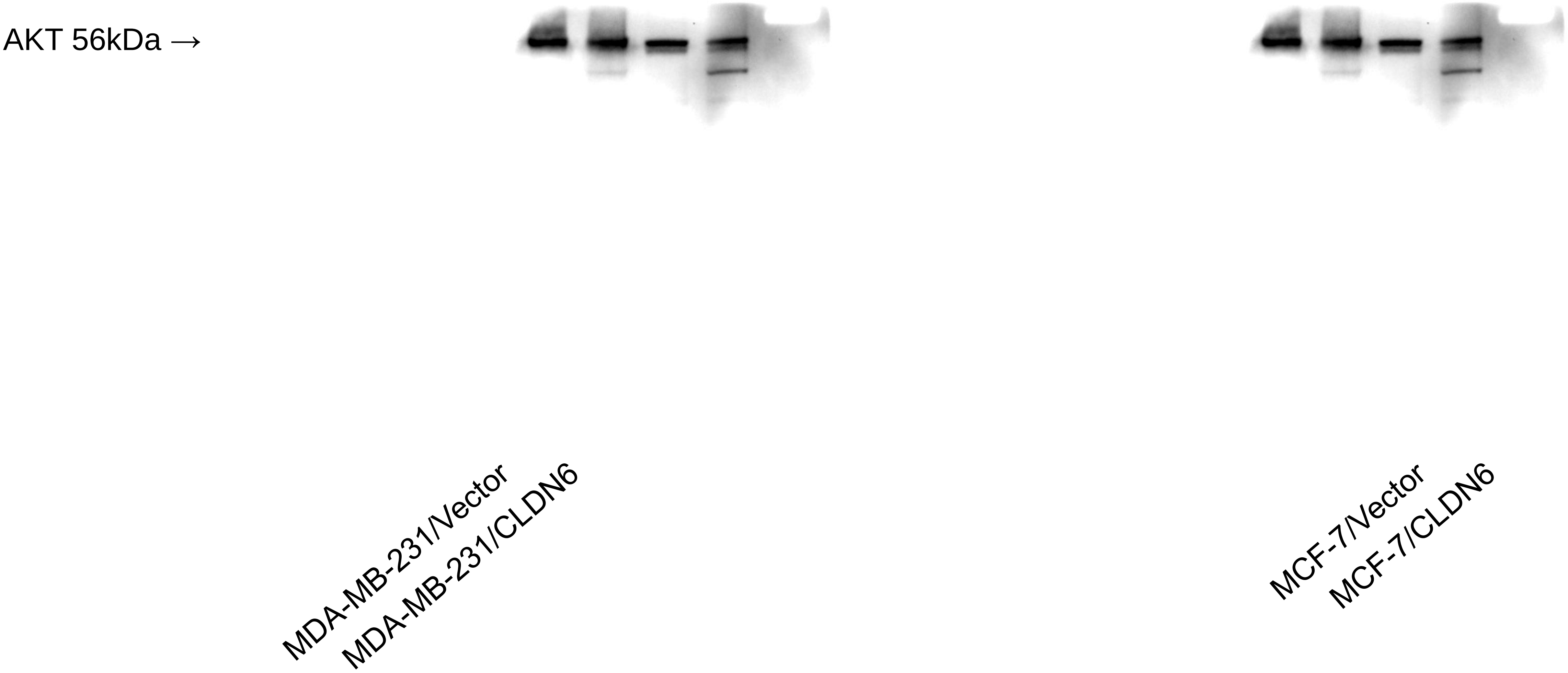
AKT 56kDa →
MDA-MB-231/Vector
MDA-MB-231/CLDN6
MCF-7/Vector
MCF-7/CLDN6

Fig. 3 F

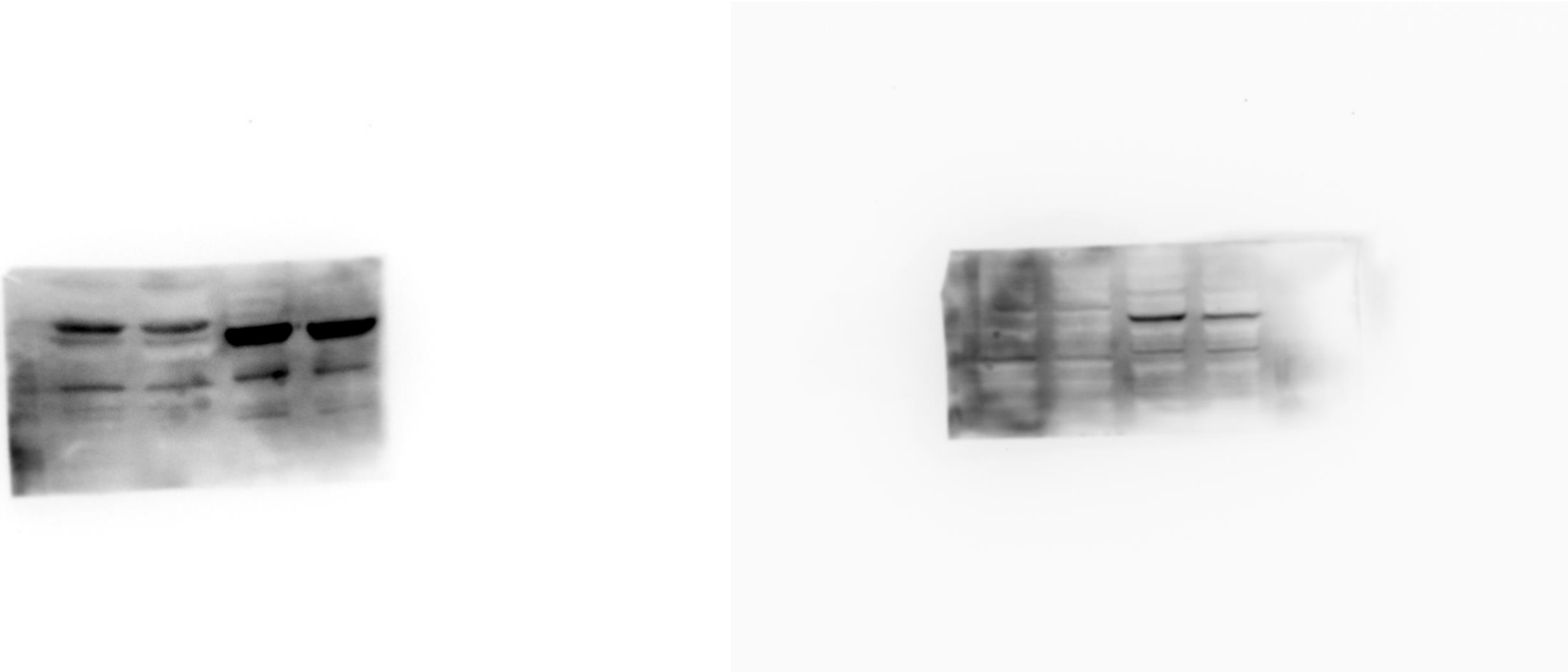
p-GSK3β 46kDa →
MDA-MB-231/Vector
MDA-MB-231/CLDN6
MCF-7/Vector
MCF-7/CLDN6

Fig. 3 F

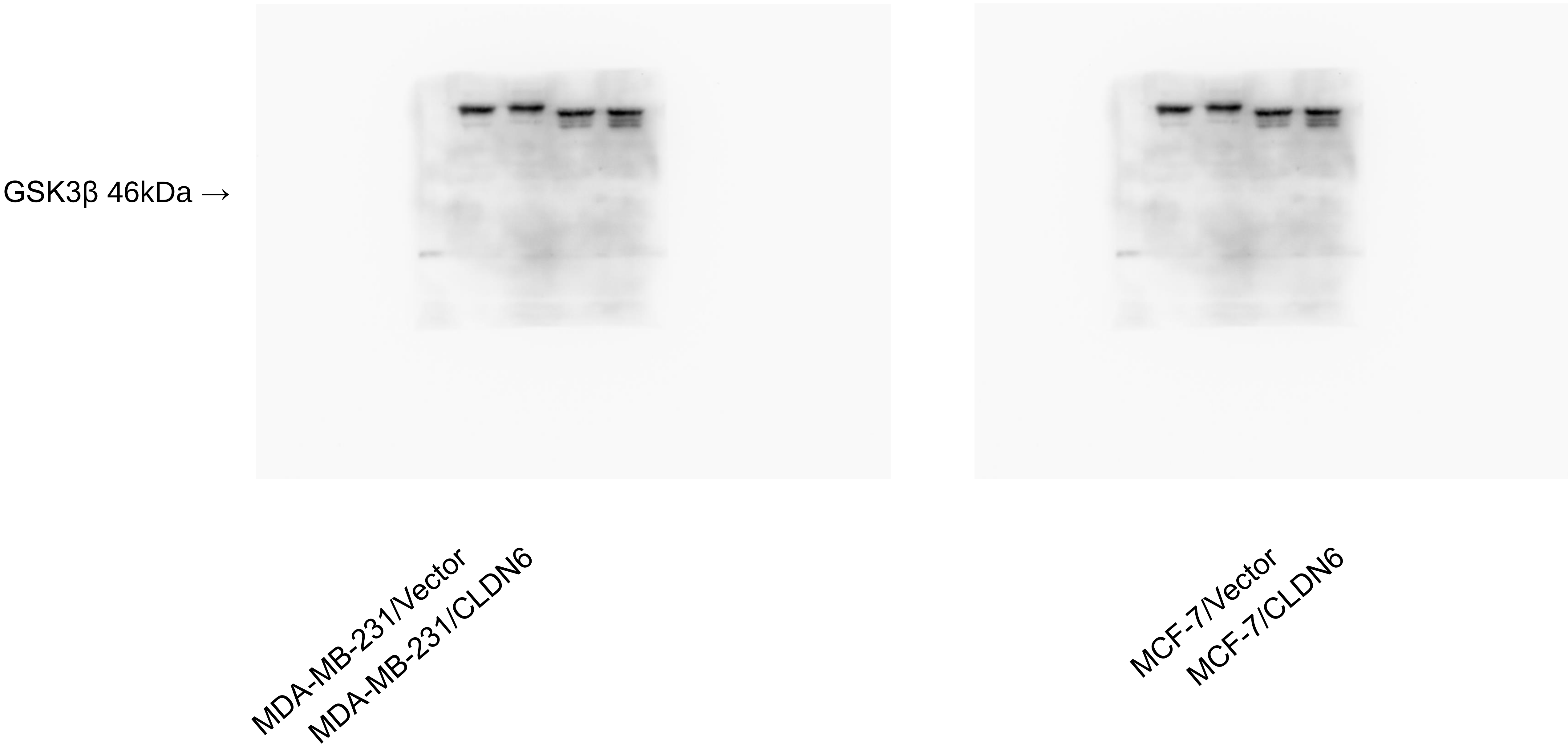
GSK3β 46kDa →
MDA-MB-231/Vector
MDA-MB-231/CLDN6
MCF-7/Vector
MCF-7/CLDN6

Fig. 3 F

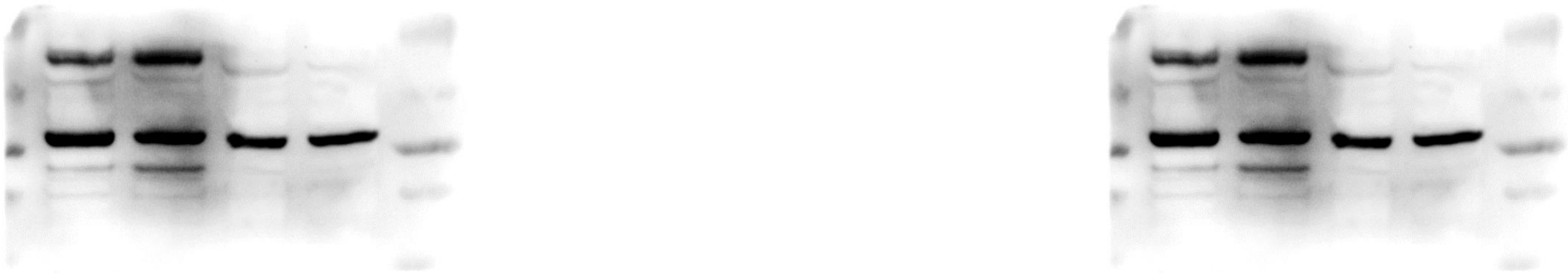
β-actin 42kDa →
MDA-MB-231/Vector
MDA-MB-231/CLDN6
MCF-7/Vector
MCF-7/CLDN6

Fig. 3 G

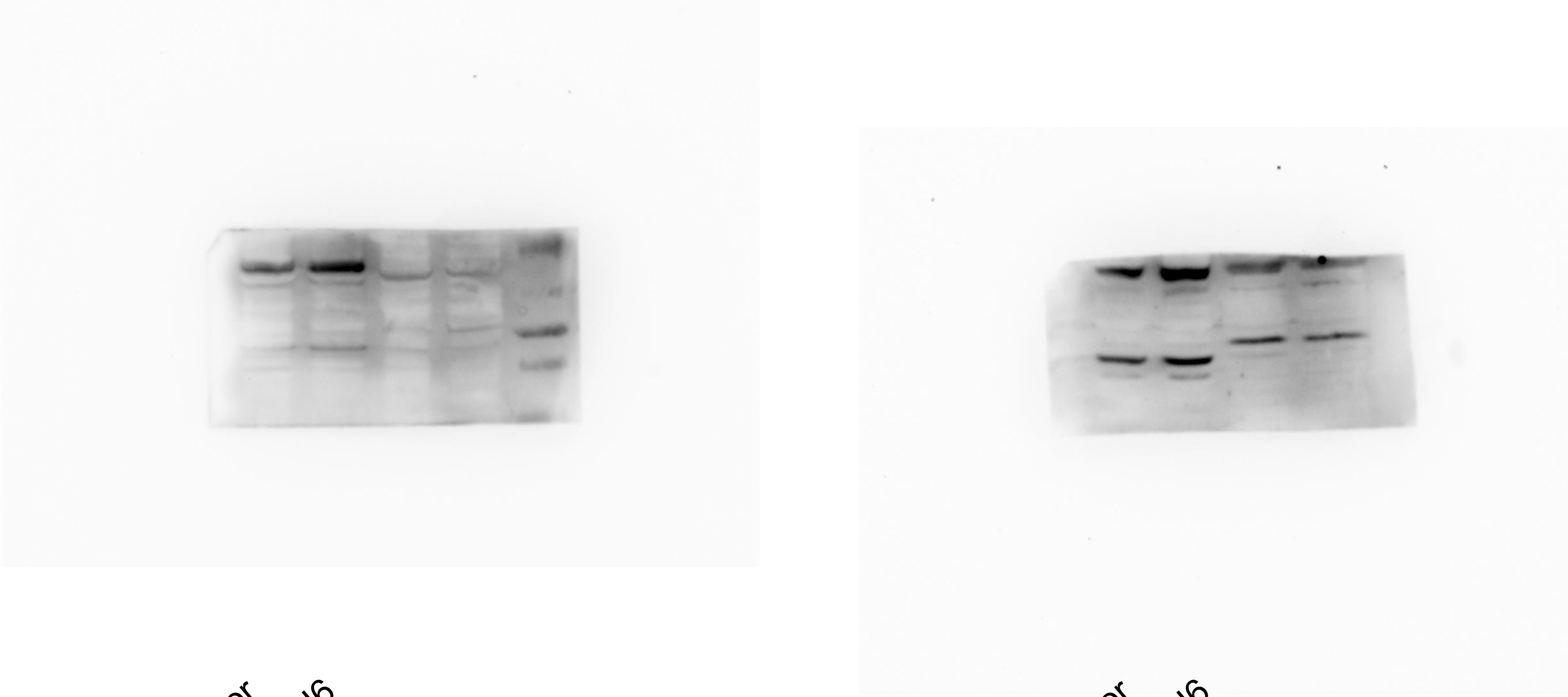

FYN 60kDa →
MDA-MB-231/Vector
MDA-MB-231/CLDN6
MCF-7/Vector
MCF-7/CLDN6

Fig. 3 G

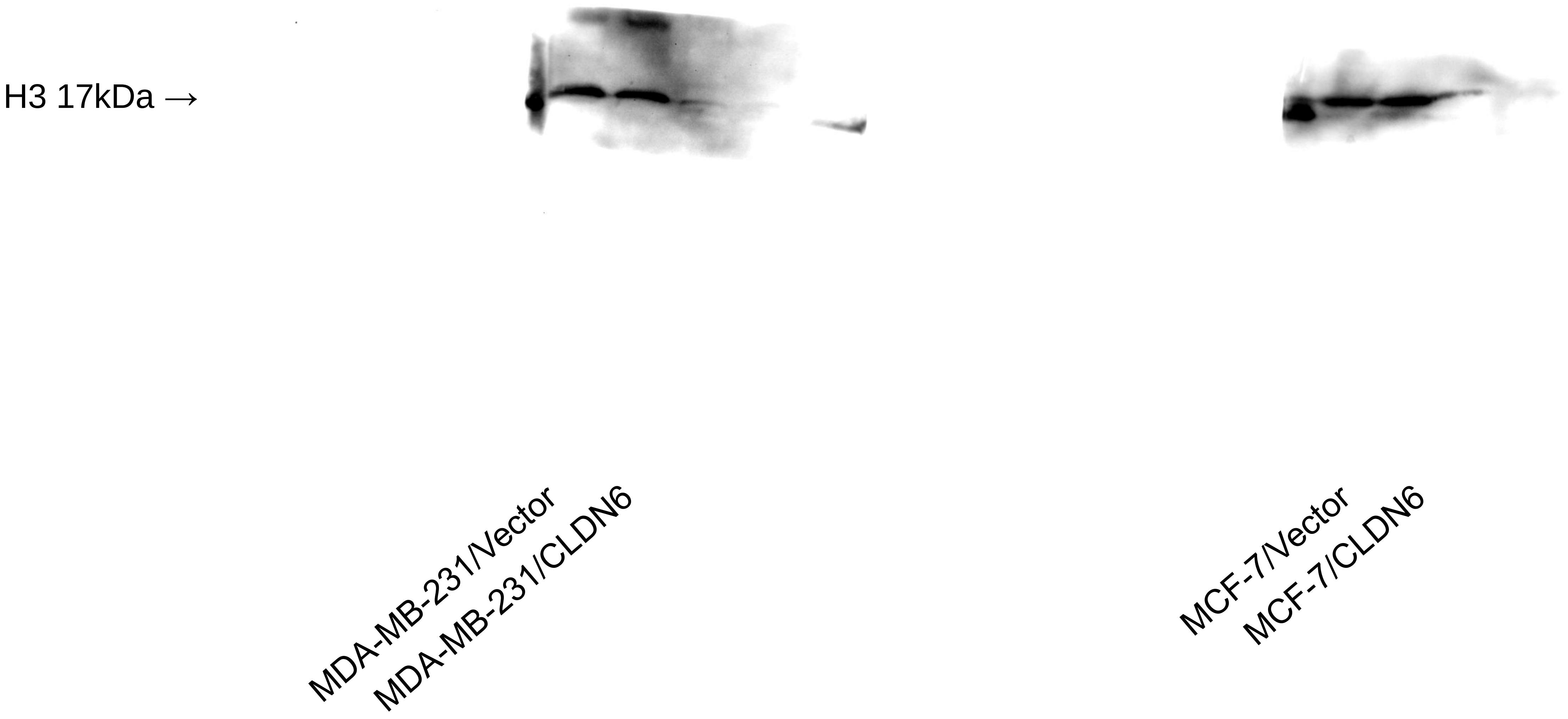
H3 17kDa →
MDA-MB-231/Vector
MDA-MB-231/CLDN6
MCF-7/Vector
MCF-7/CLDN6

Fig. 3 G

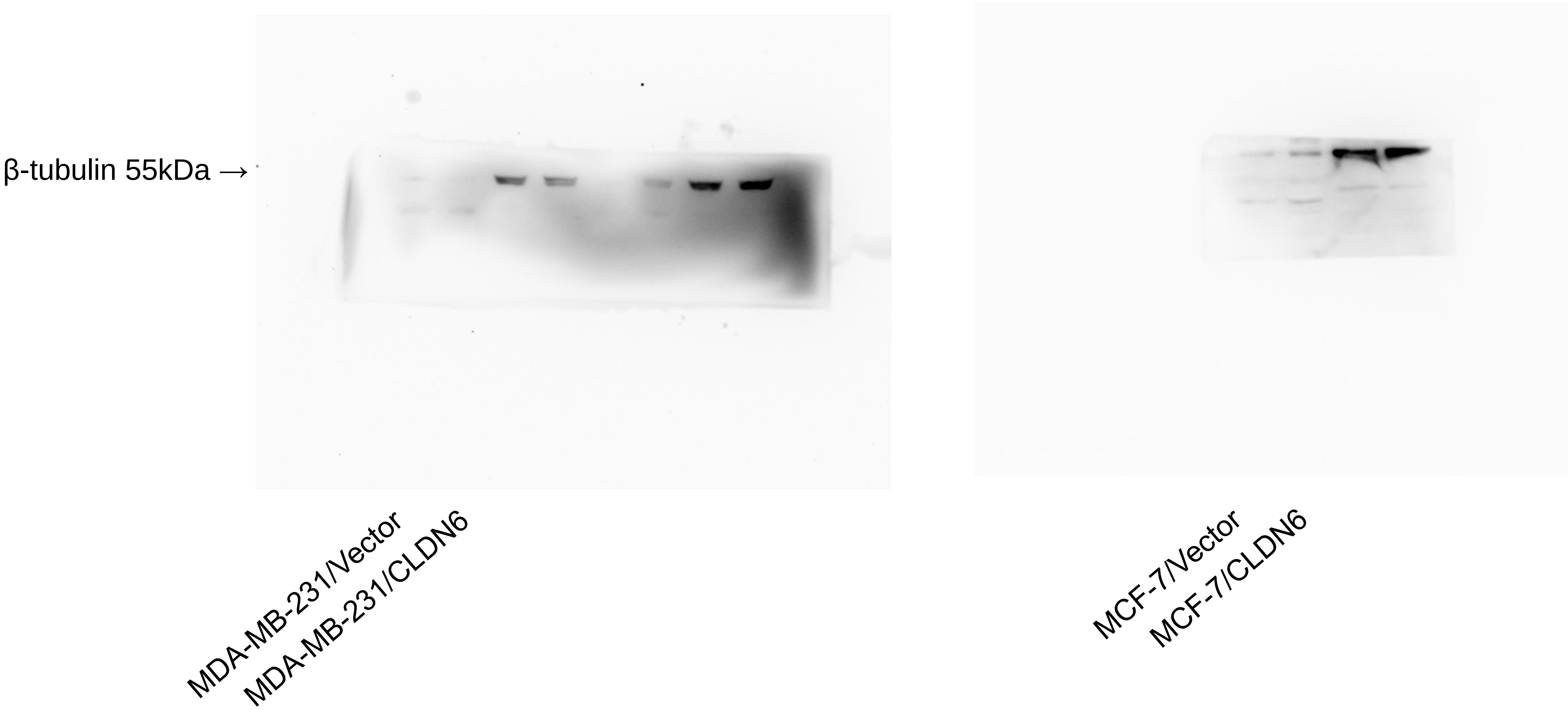
β-tubulin 55kDa →
MDA-MB-231/Vector
MDA-MB-231/CLDN6
MCF-7/Vector
MCF-7/CLDN6

Fig. 3 I

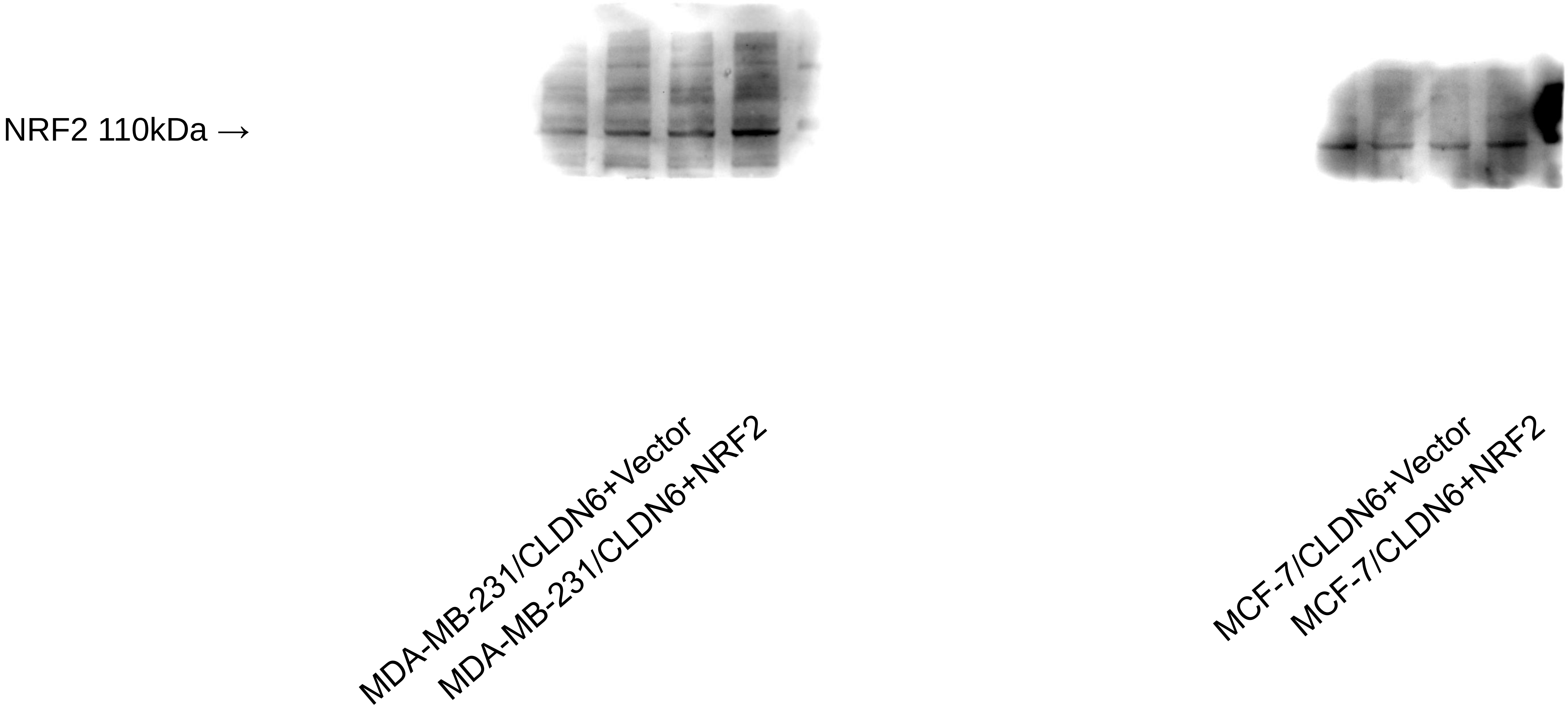

NRF2 110kDa →
MDA-MB-231/CLDN6+Vector
MDA-MB-231/CLDN6+NRF2
MCF-7/CLDN6+Vector
MCF-7/CLDN6+NRF2

Fig. 3 l

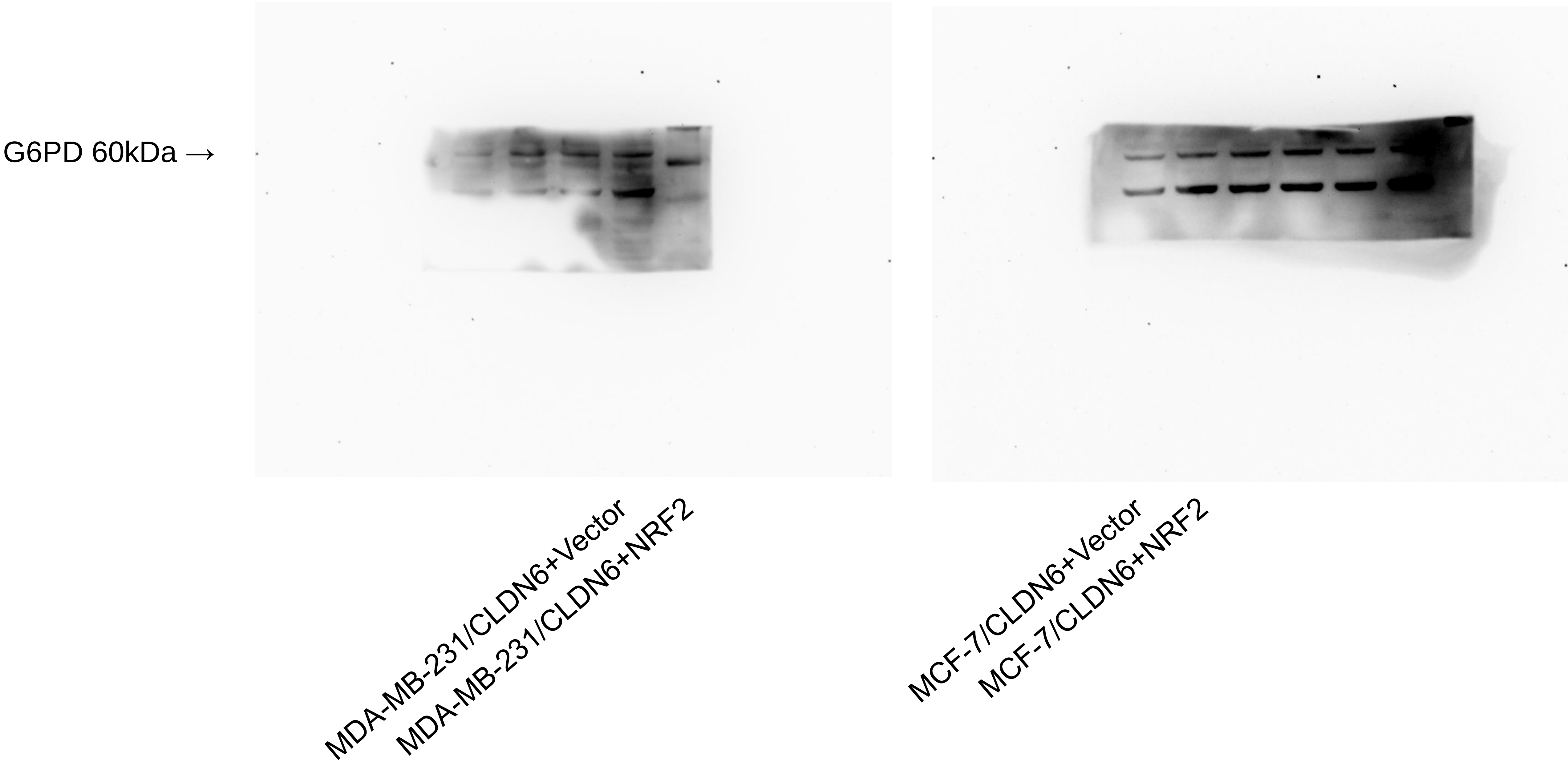
G6PD 60kDa →
MDA-MB-231/CLDN6+Vector
MDA-MB-231/CLDN6+NRF2
MCF-7/CLDN6+Vector
MCF-7/CLDN6+NRF2

Fig. 3 I

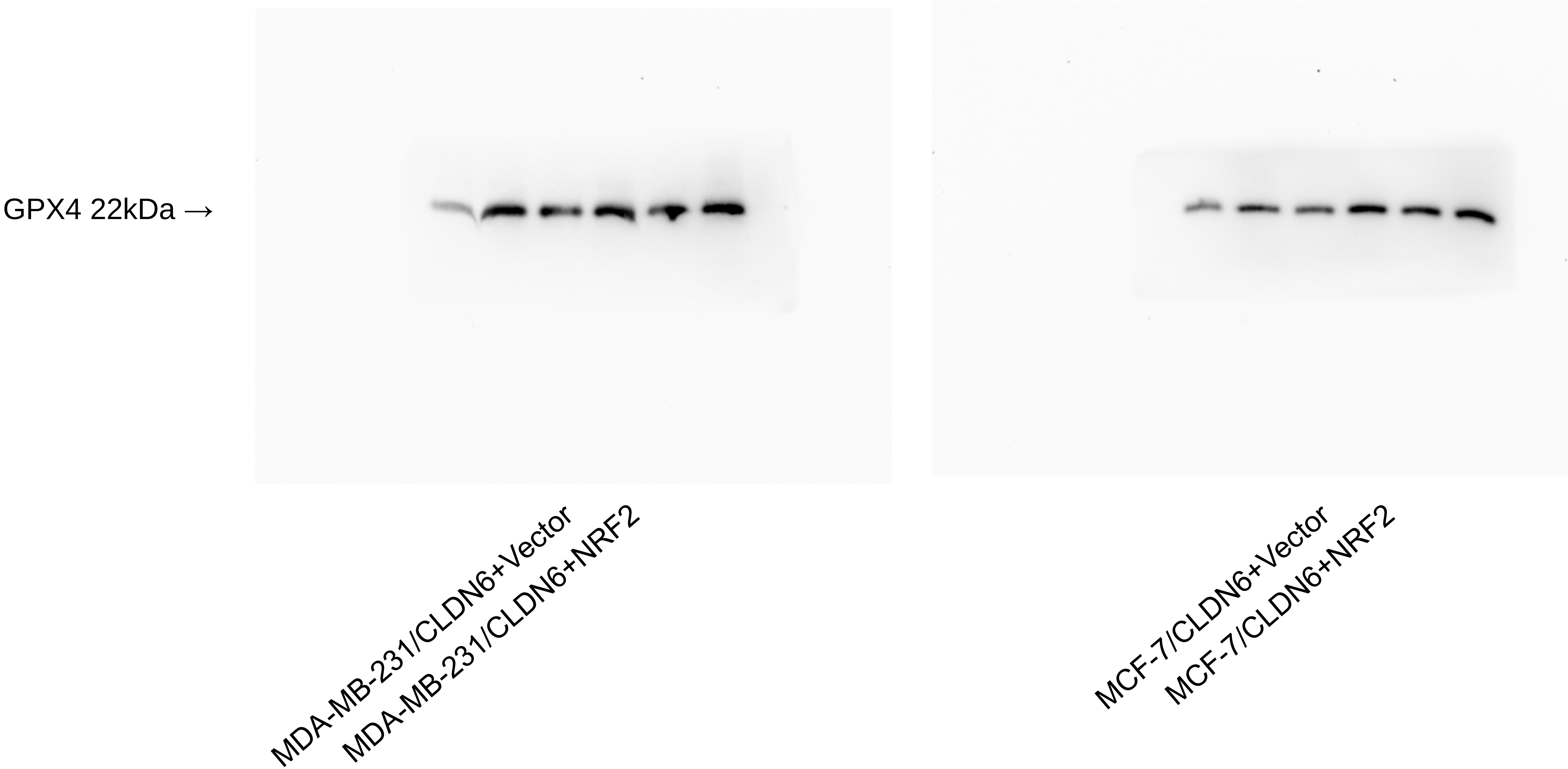
GPX4 22kDa →
MDA-MB-231/CLDN6+Vector
MDA-MB-231/CLDN6+NRF2
MCF-7/CLDN6+Vector
MCF-7/CLDN6+NRF2

Fig. 3 I

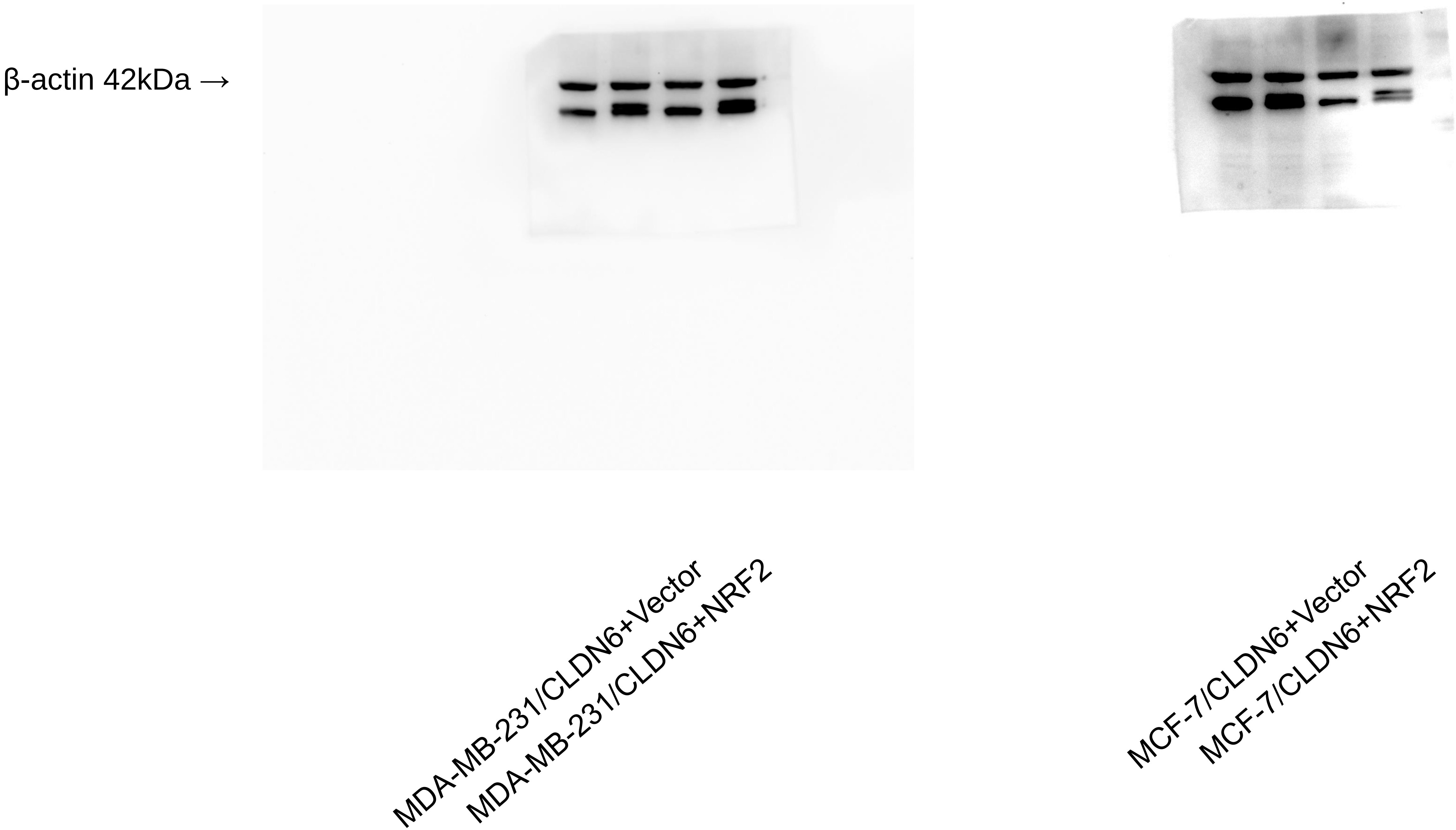

β-actin 42kDa →
MDA-MB-231/CLDN6+Vector
MDA-MB-231/CLDN6+NRF2
MCF-7/CLDN6+Vector
MCF-7/CLDN6+NRF2

Fig. 4 A

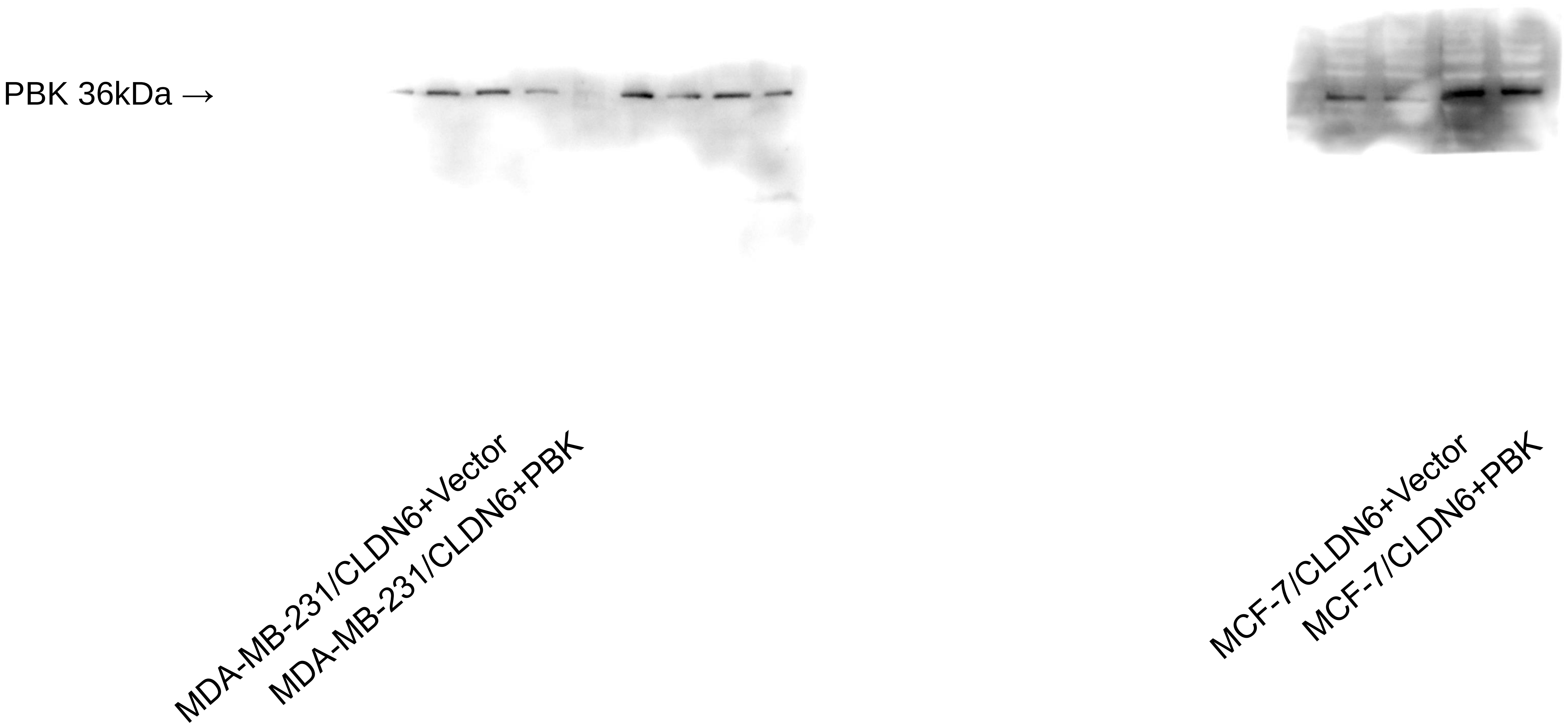
PBK 36kDa →
MDA-MB-231/CLDN6+Vector
MDA-MB-231/CLDN6+PBK
MCF-7/CLDN6+Vector
MCF-7/CLDN6+PBK

Fig. 4 A

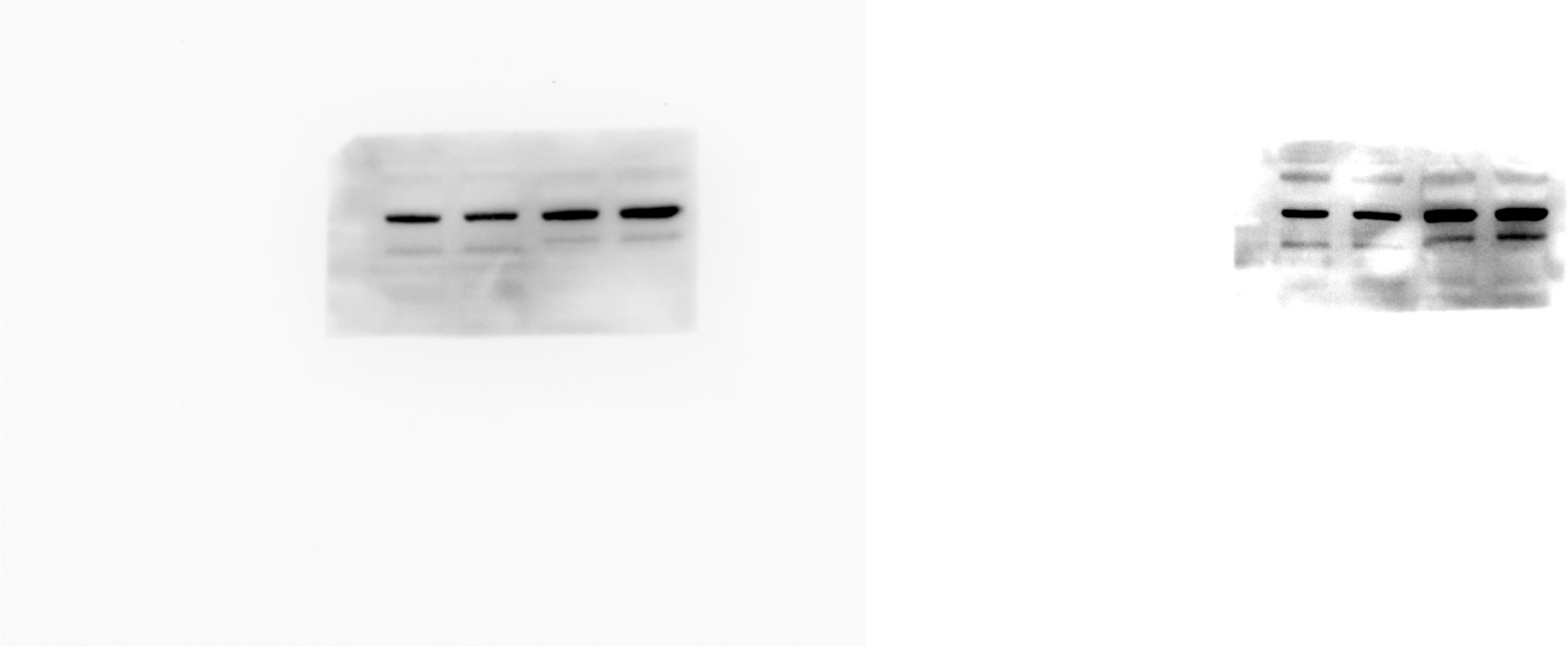
β-actin 42kDa →
MDA-MB-231/CLDN6+Vector
MDA-MB-231/CLDN6+PBK
MCF-7/CLDN6+Vector
MCF-7/CLDN6+PBK

Fig. 4 B

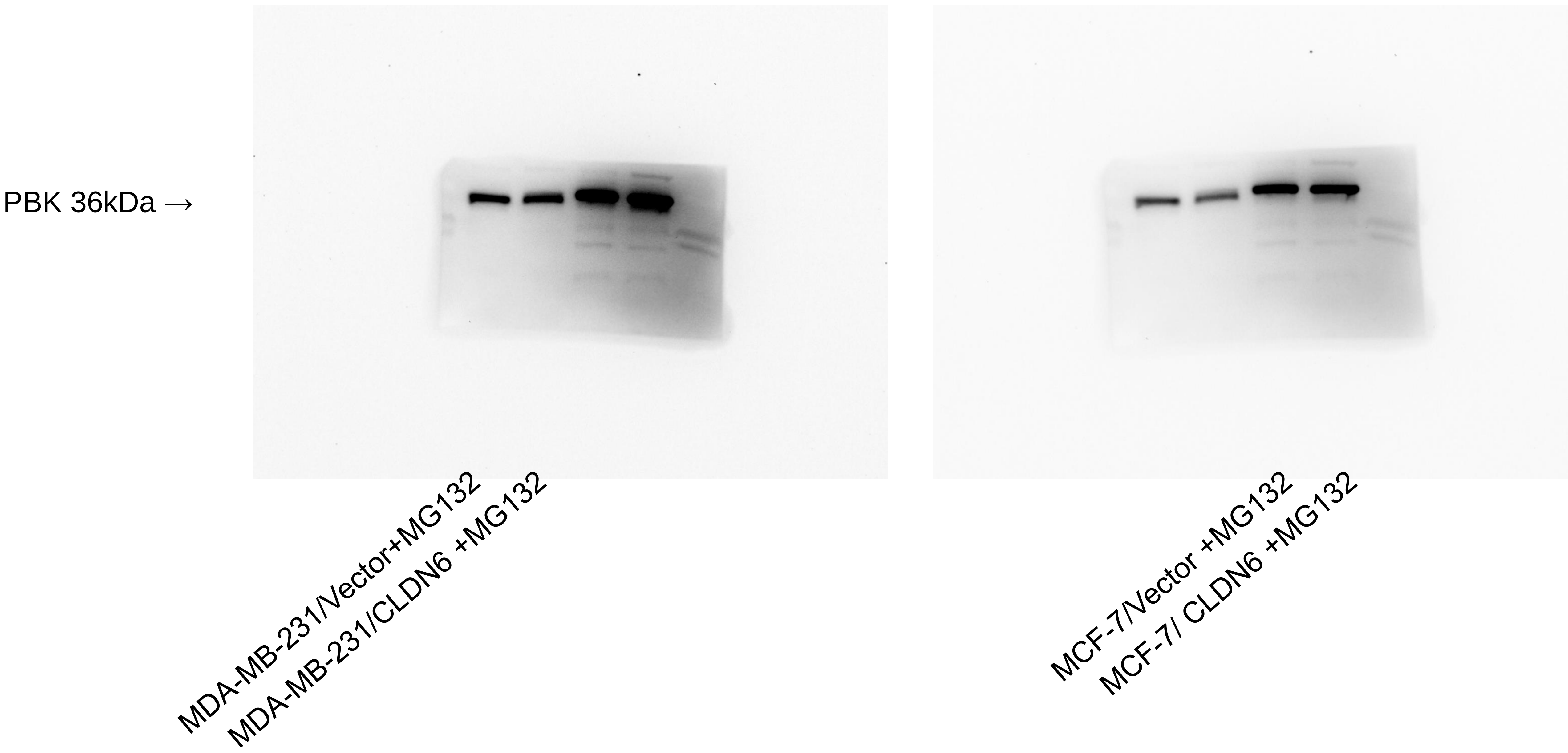
PBK 36kDa →
MDA-MB-231/Vector+MG132
MDA-MB-231/CLDN6 +MG132
MCF-7/Vector +MG132
MCF-7/ CLDN6 +MG132

Fig. 4 B

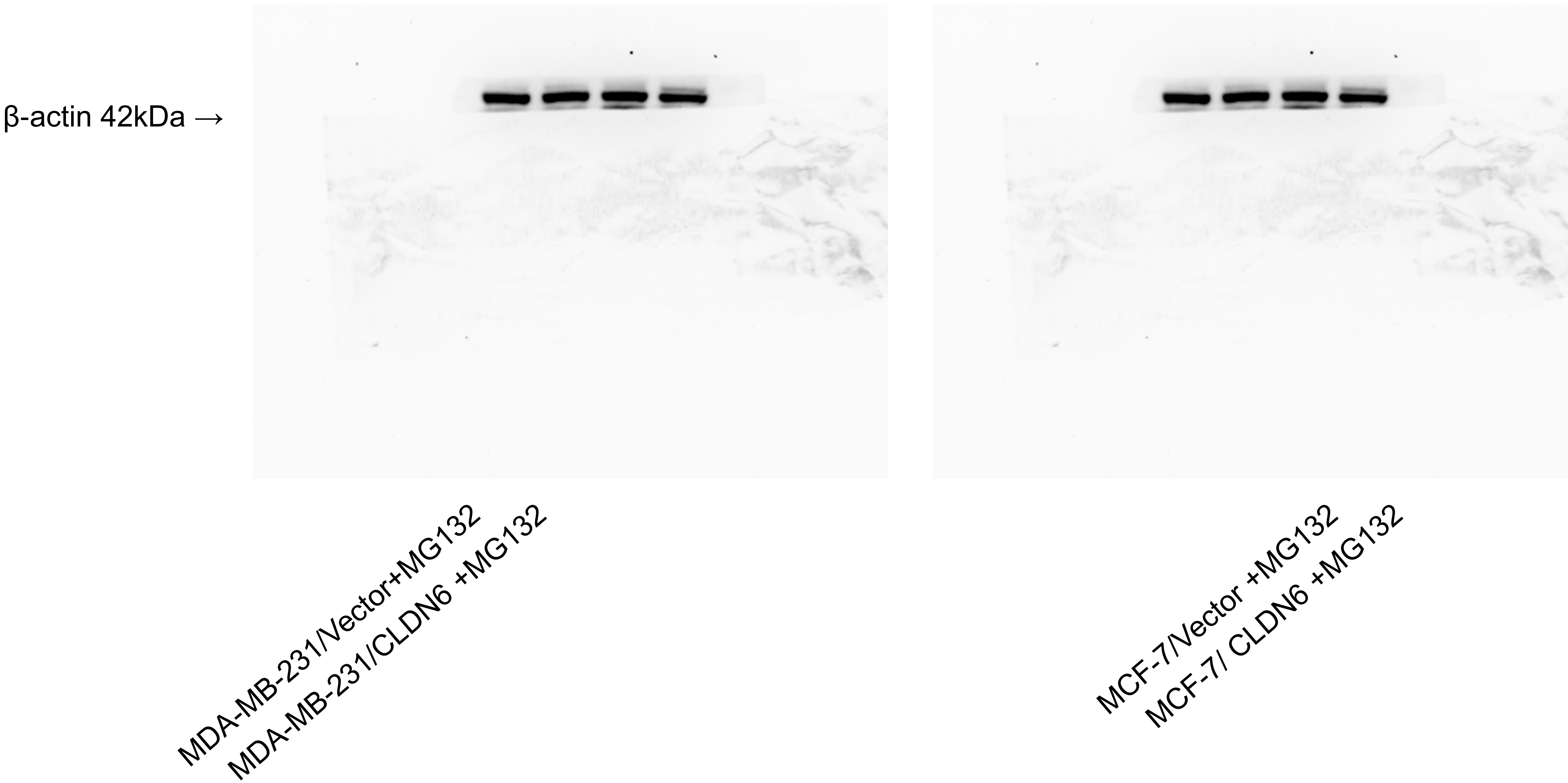
β-actin 42kDa →
MDA-MB-231/Vector+MG132
MDA-MB-231/CLDN6 +MG132
MCF-7/Vector +MG132
MCF-7/ CLDN6 +MG132

Fig. 4 C

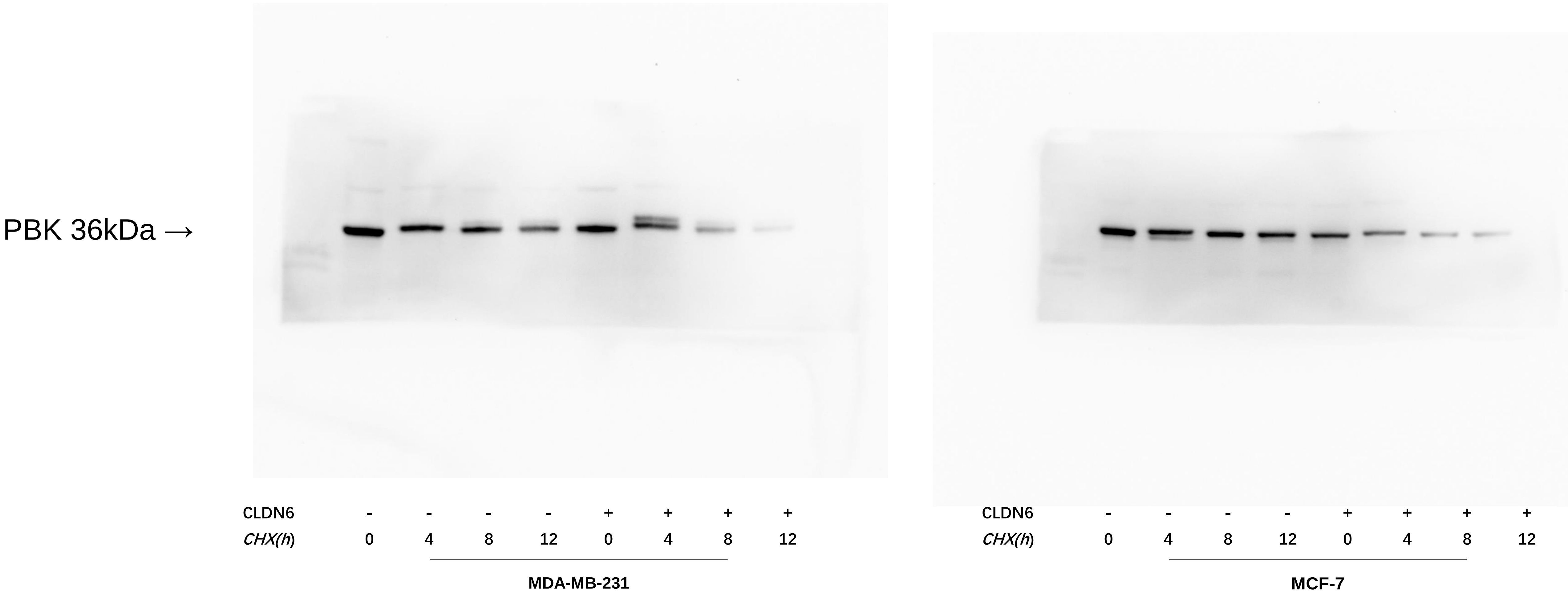
PBK 36kDa →
CLDN6 - - - - + + + +
CHX(h) 0 4 8 12 0 4 8 12
MDA-MB-231
CLDN6 - - - - + + + +
CHX(h) 0 4 8 12 0 4 8 12
MCF-7

Fig. 4 C

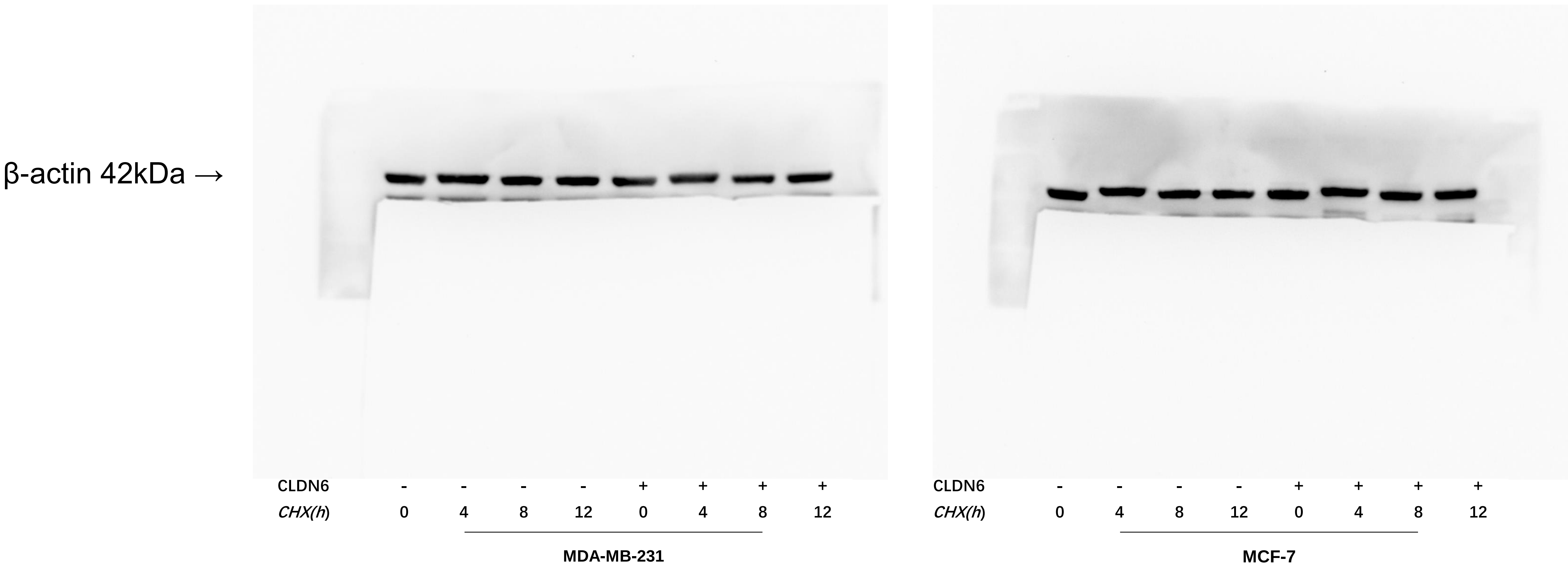
β-actin 42kDa →
CLDN6 - - - - + + + +
CHX(h) 0 4 8 12 0 4 8 12
MDA-MB-231
CLDN6 - - - - + + + +
CHX(h) 0 4 8 12 0 4 8 12
MCF-7

Fig. 4 D

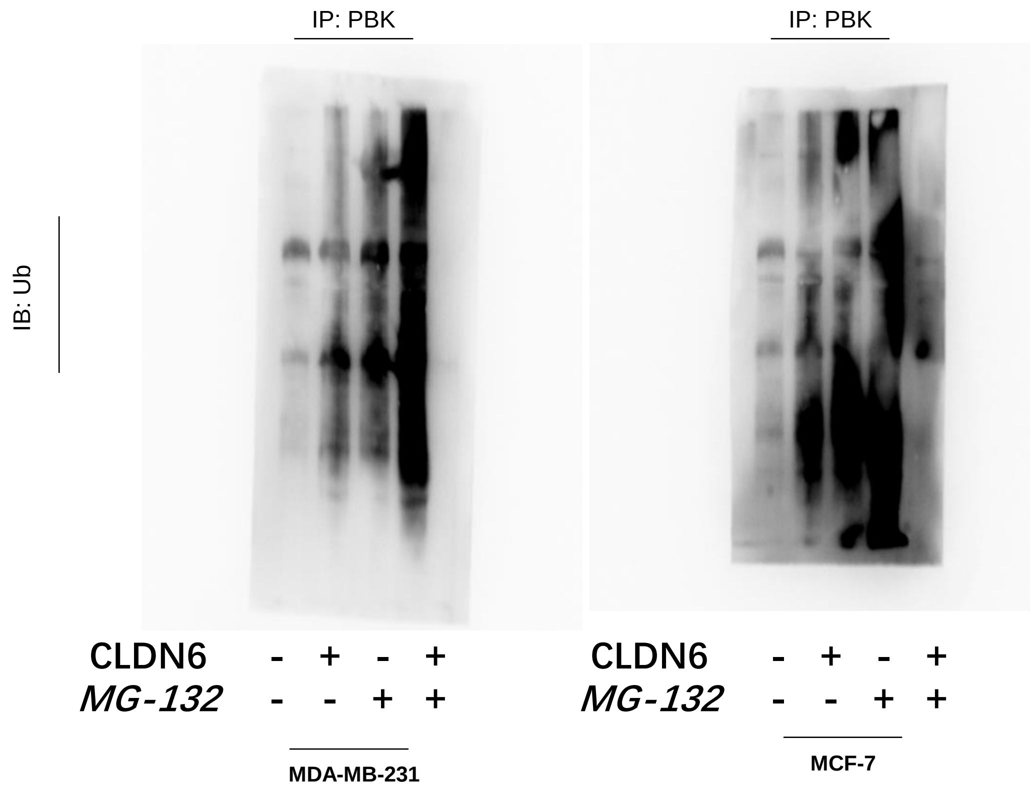

Fig. 4 D

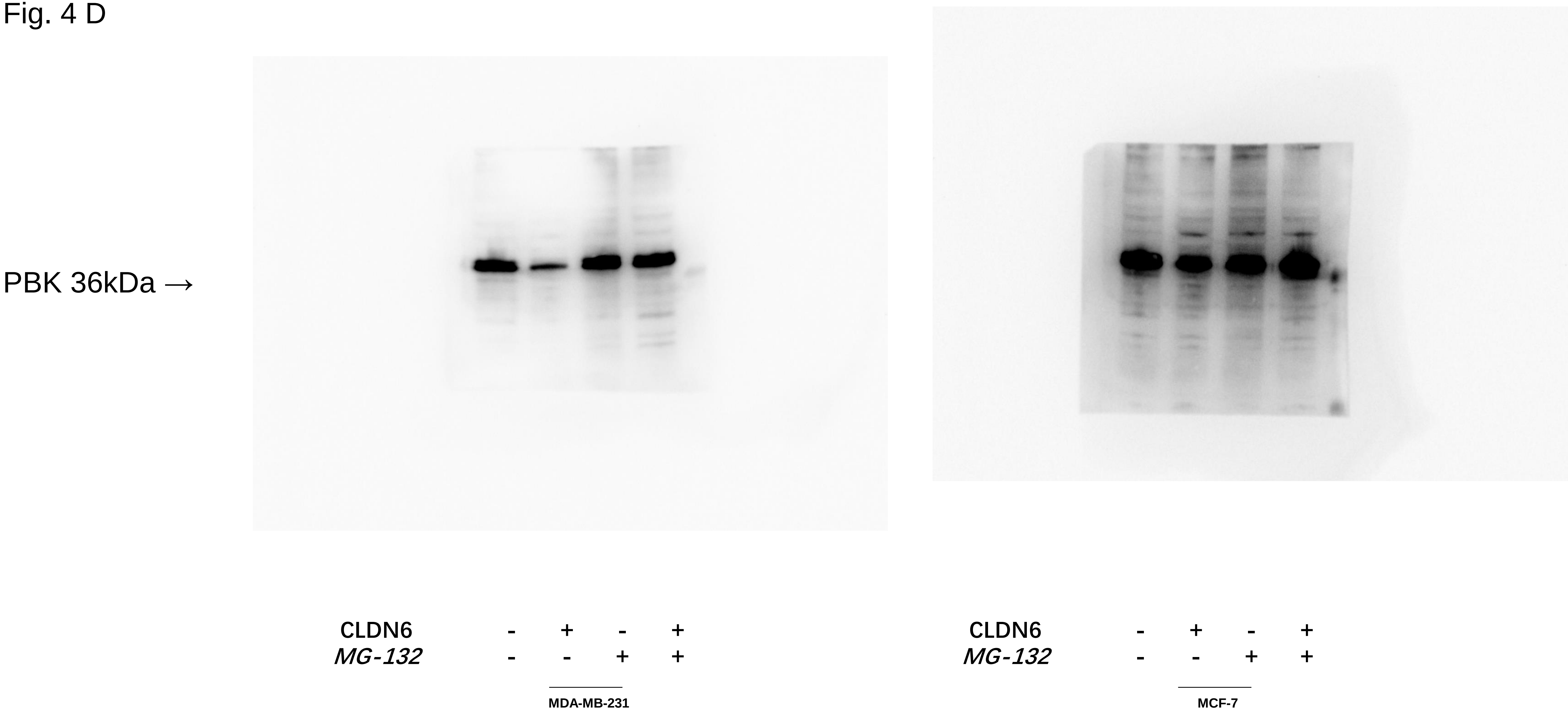

Fig. 4 D

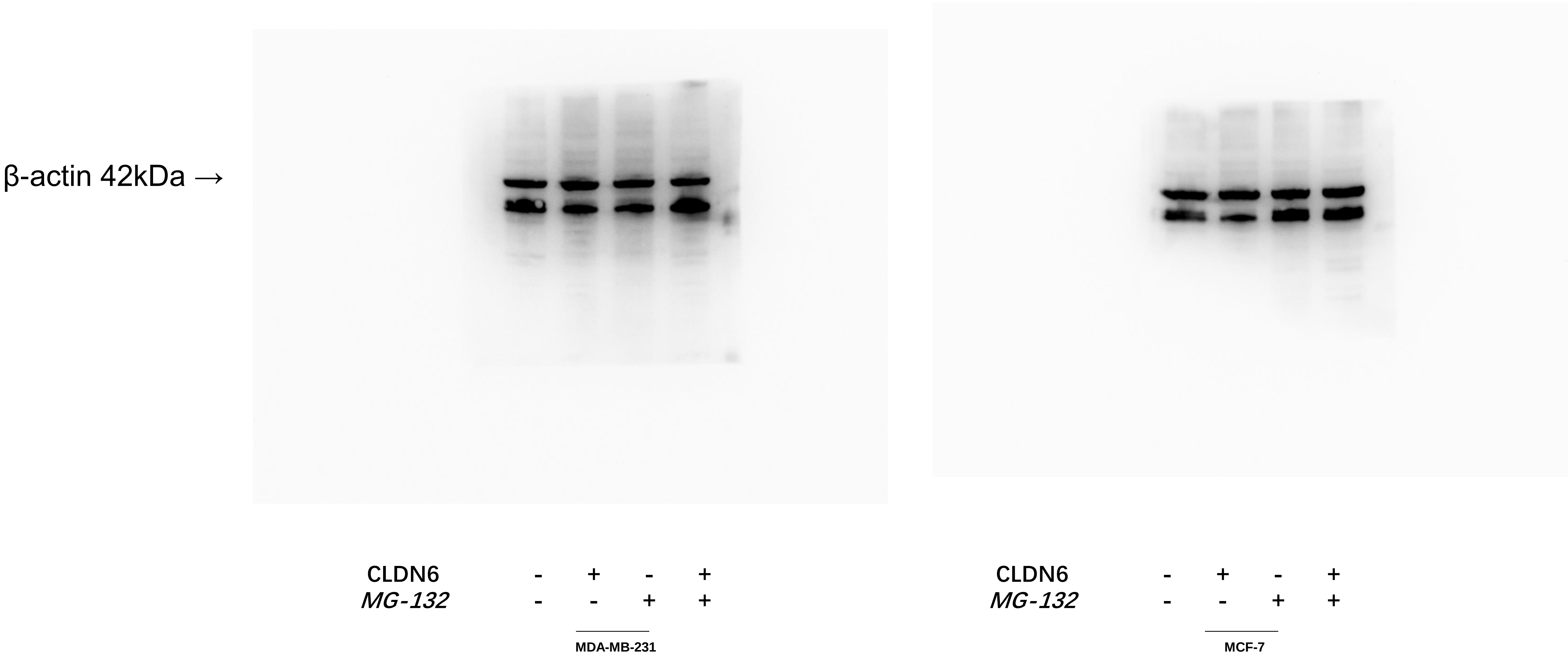

Fig. 4 E

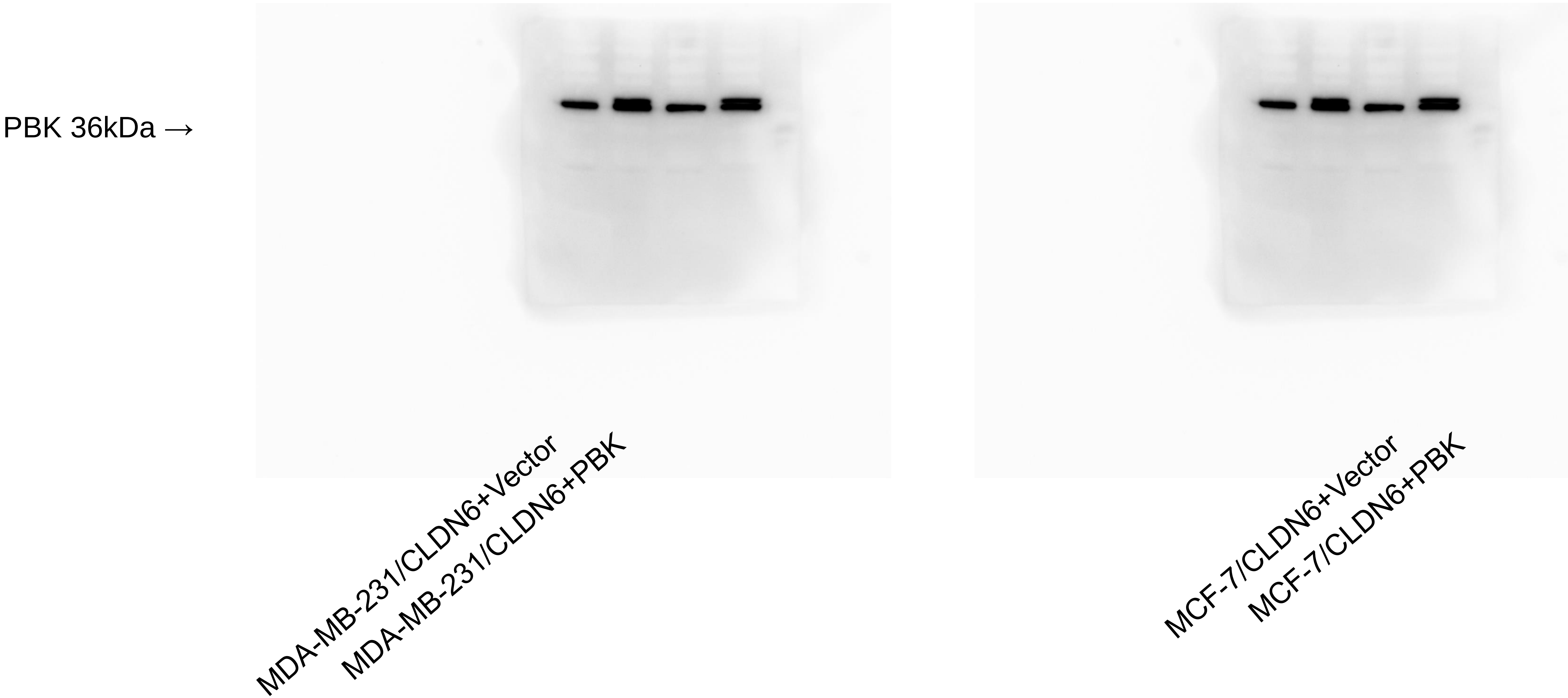
PBK 36kDa →
MDA-MB-231/CLDN6+Vector
MDA-MB-231/CLDN6+PBK
MCF-7/CLDN6+Vector
MCF-7/CLDN6+PBK

Fig. 4 E

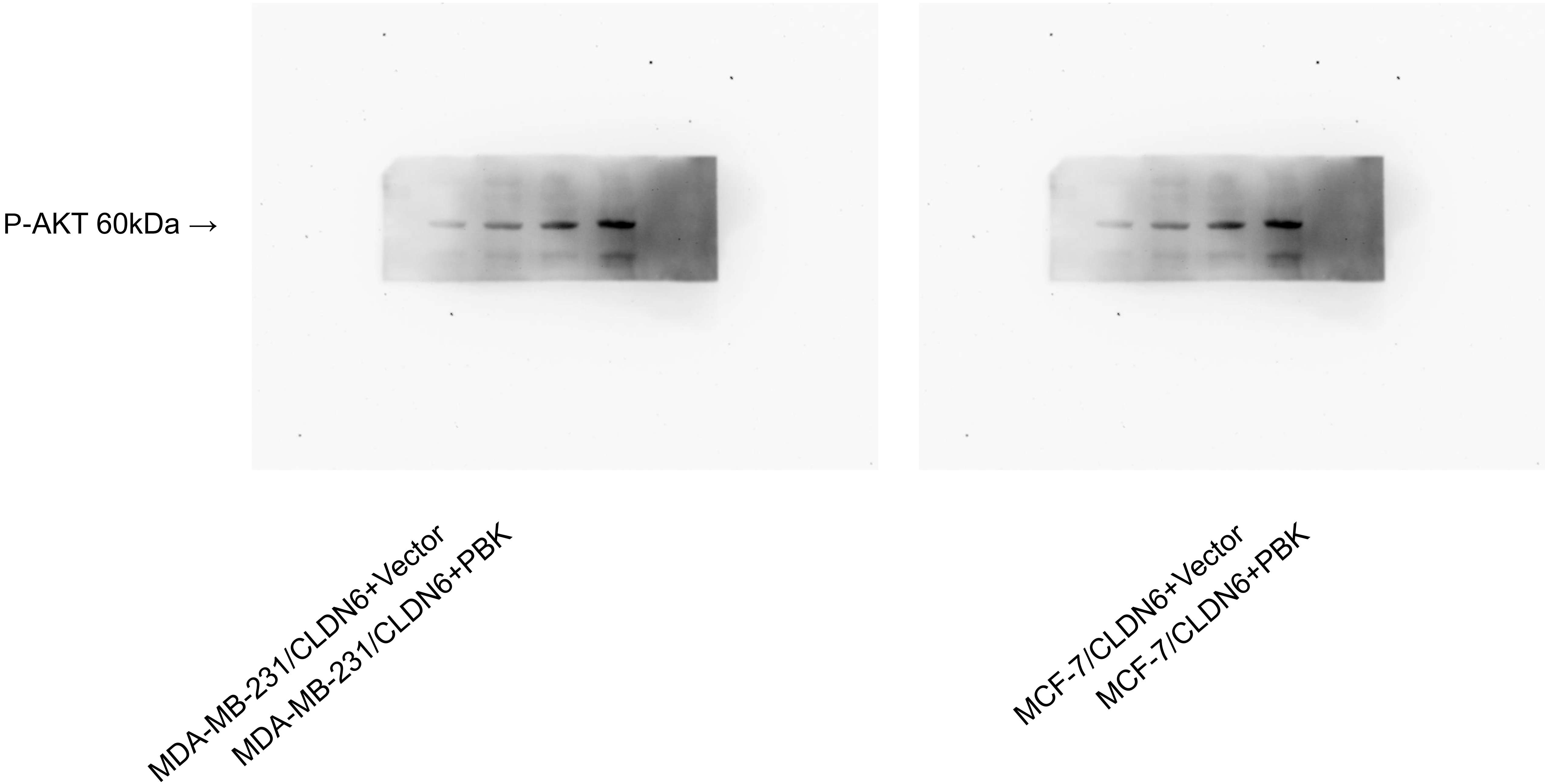
P-AKT 60kDa →
MDA-MB-231/CLDN6+Vector
MDA-MB-231/CLDN6+PBK
MCF-7/CLDN6+Vector
MCF-7/CLDN6+PBK

Fig. 4 E

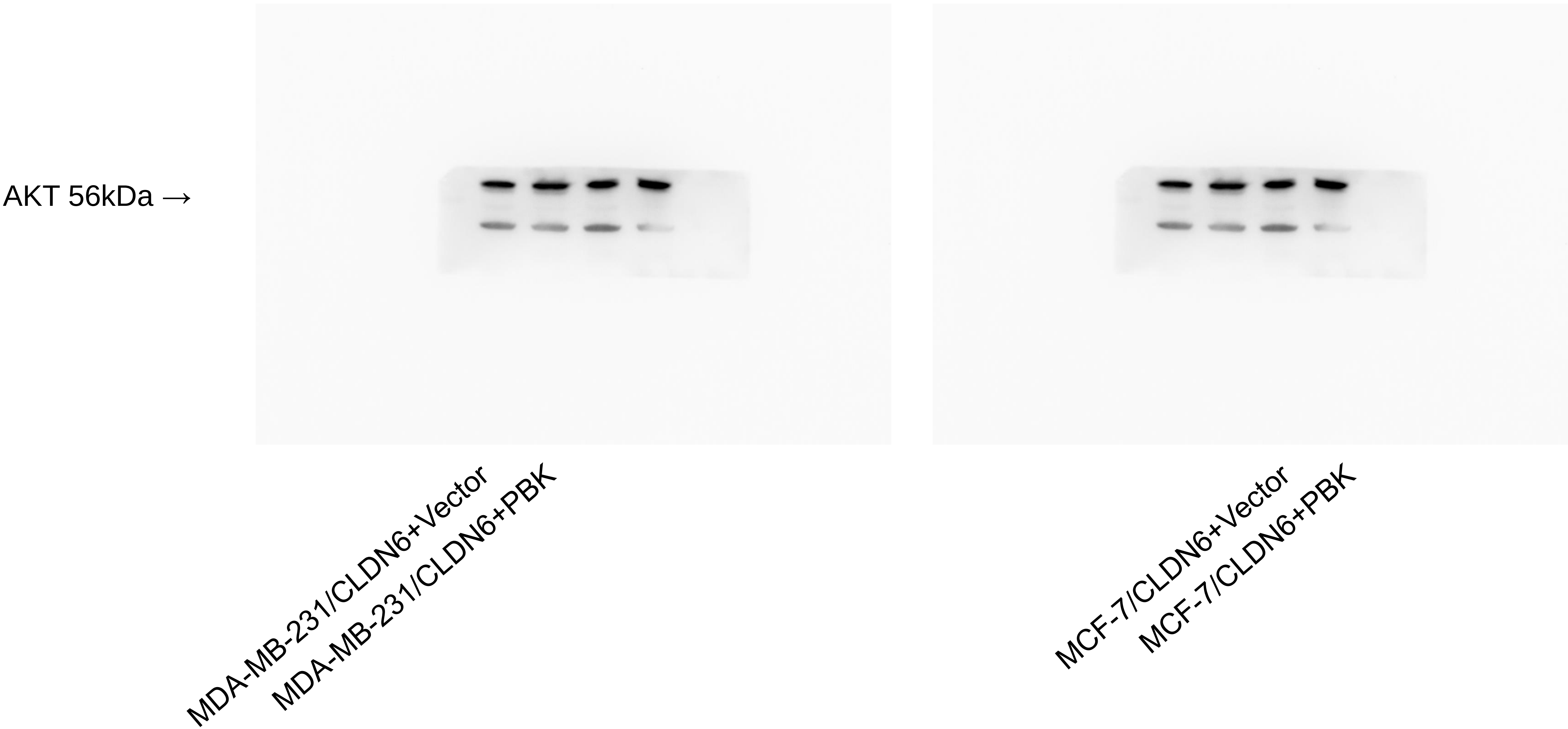
AKT 56kDa →
MDA-MB-231/CLDN6+Vector
MDA-MB-231/CLDN6+PBK
MCF-7/CLDN6+Vector
MCF-7/CLDN6+PBK

Fig. 4 E

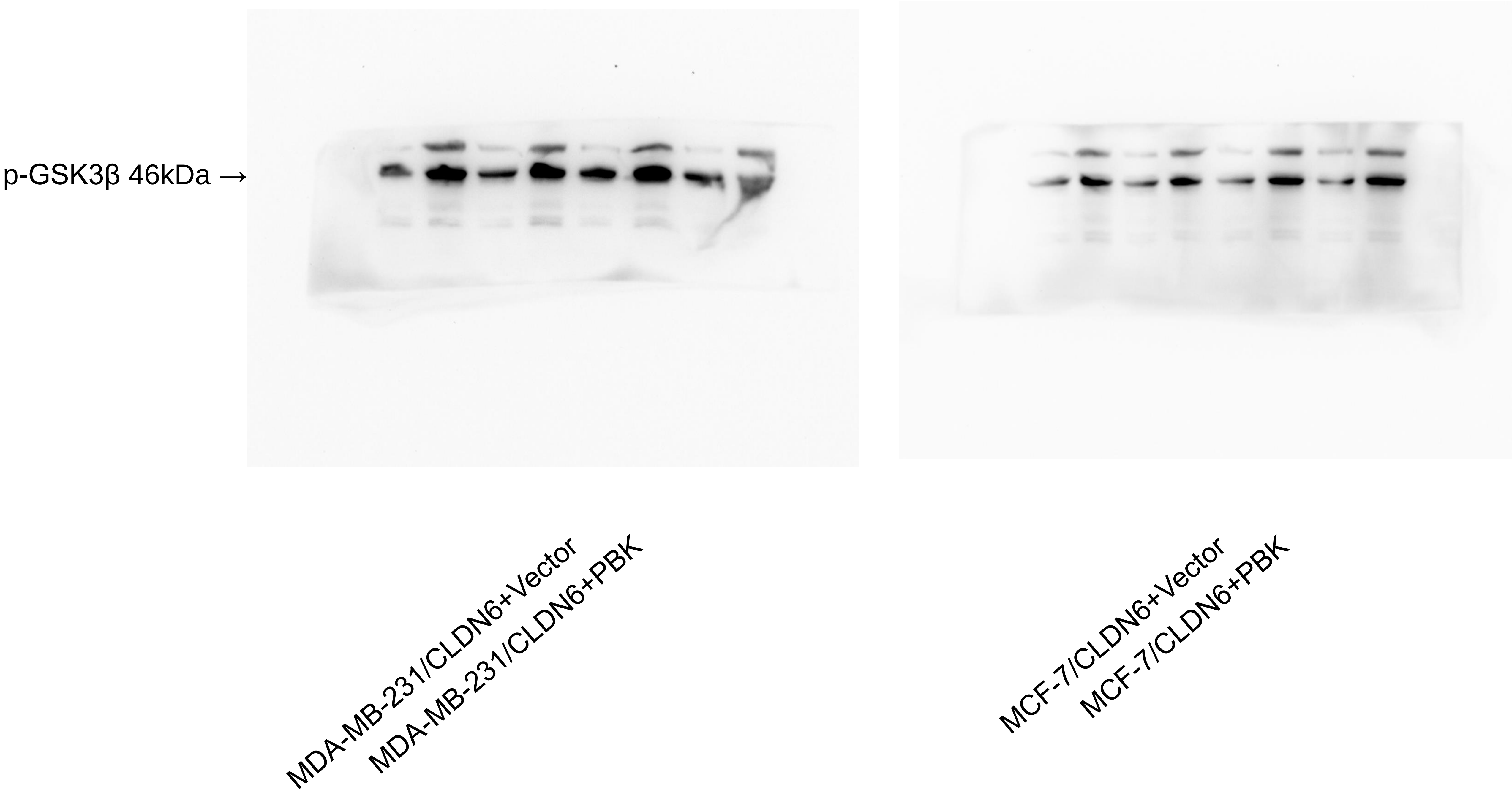
p-GSK3β 46kDa →
MDA-MB-231/CLDN6+Vector
MDA-MB-231/CLDN6+PBK
MCF-7/CLDN6+Vector
MCF-7/CLDN6+PBK

Fig. 4 E

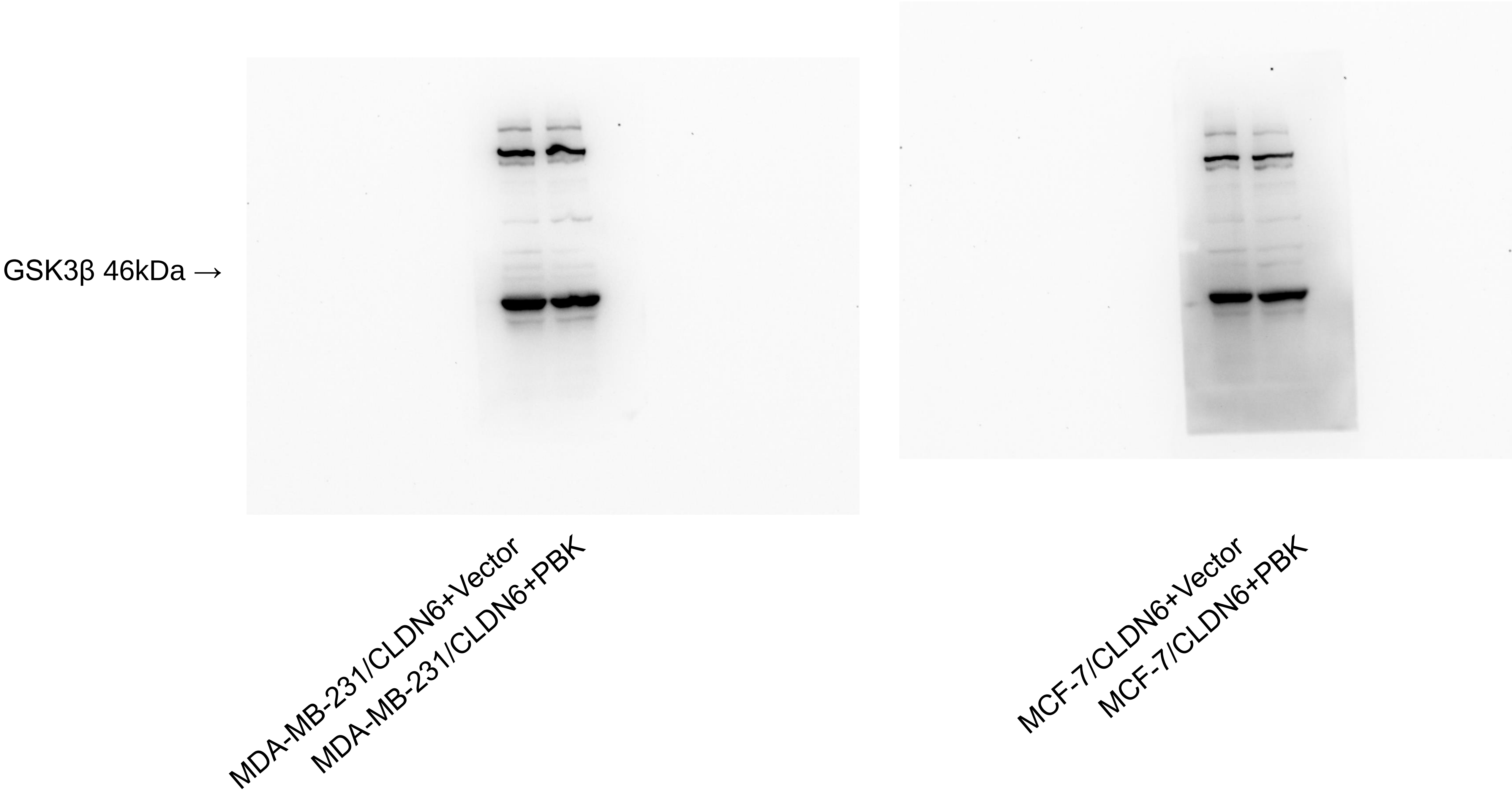
GSK3β 46kDa →
MDA-MB-231/CLDN6+Vector
MDA-MB-231/CLDN6+PBK
MCF-7/CLDN6+Vector
MCF-7/CLDN6+PBK

Fig. 4 E

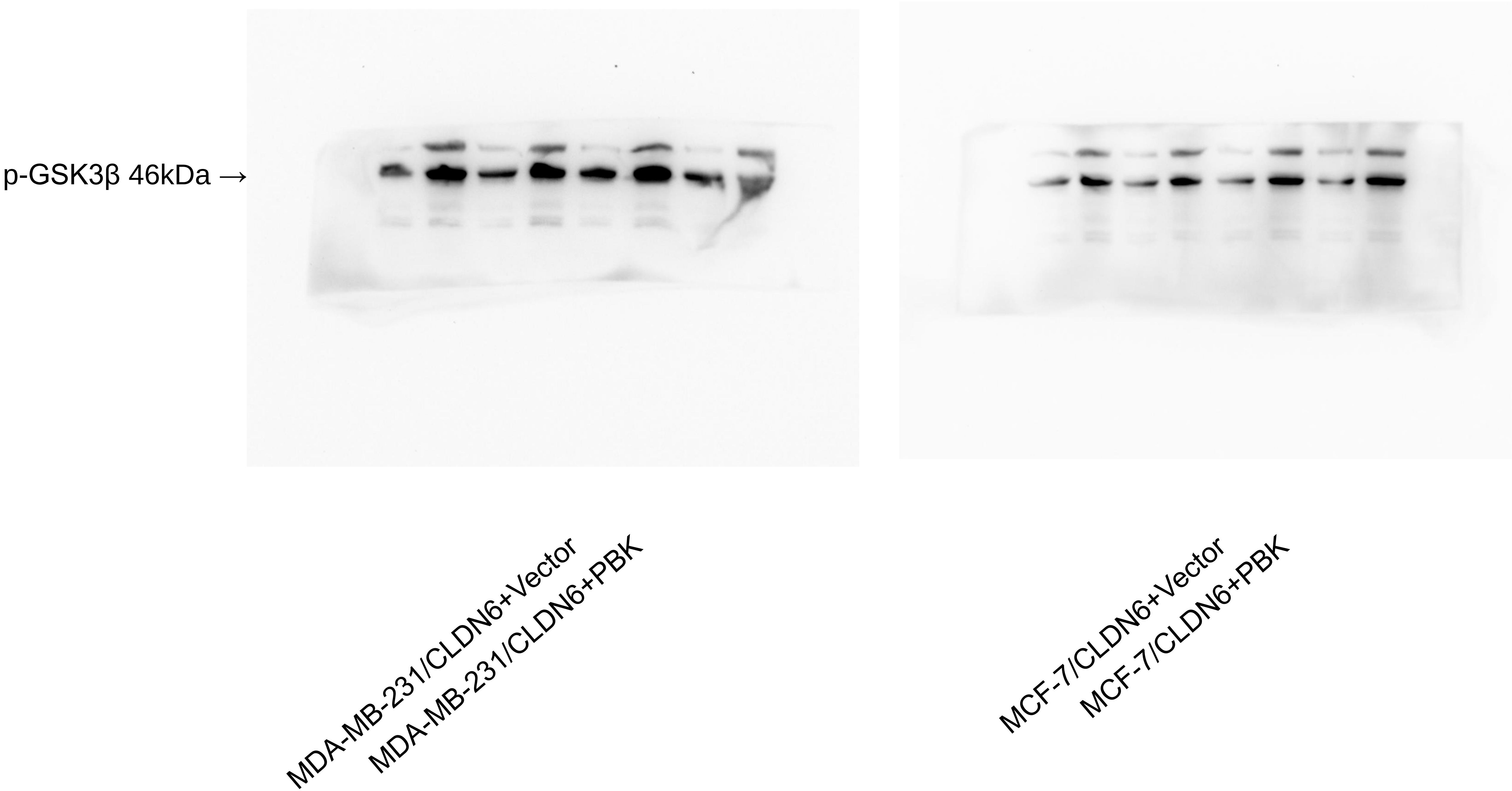
p-GSK3β 46kDa →
MDA-MB-231/CLDN6+Vector
MDA-MB-231/CLDN6+PBK
MCF-7/CLDN6+Vector
MCF-7/CLDN6+PBK

Fig. 4 E

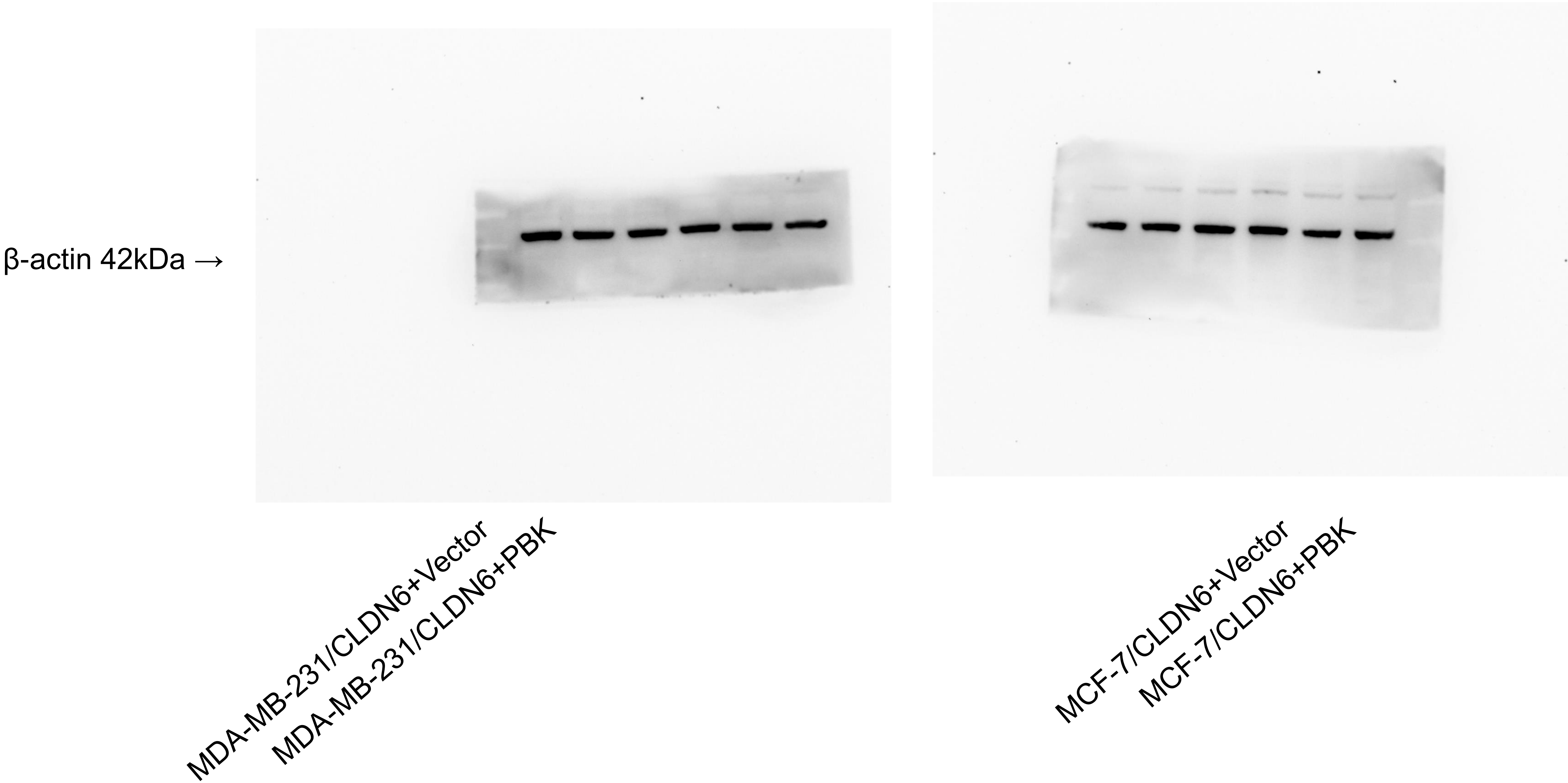

β-actin 42kDa →
MDA-MB-231/CLDN6+Vector
MDA-MB-231/CLDN6+PBK
MCF-7/CLDN6+Vector
MCF-7/CLDN6+PBK

Fig. 4 F

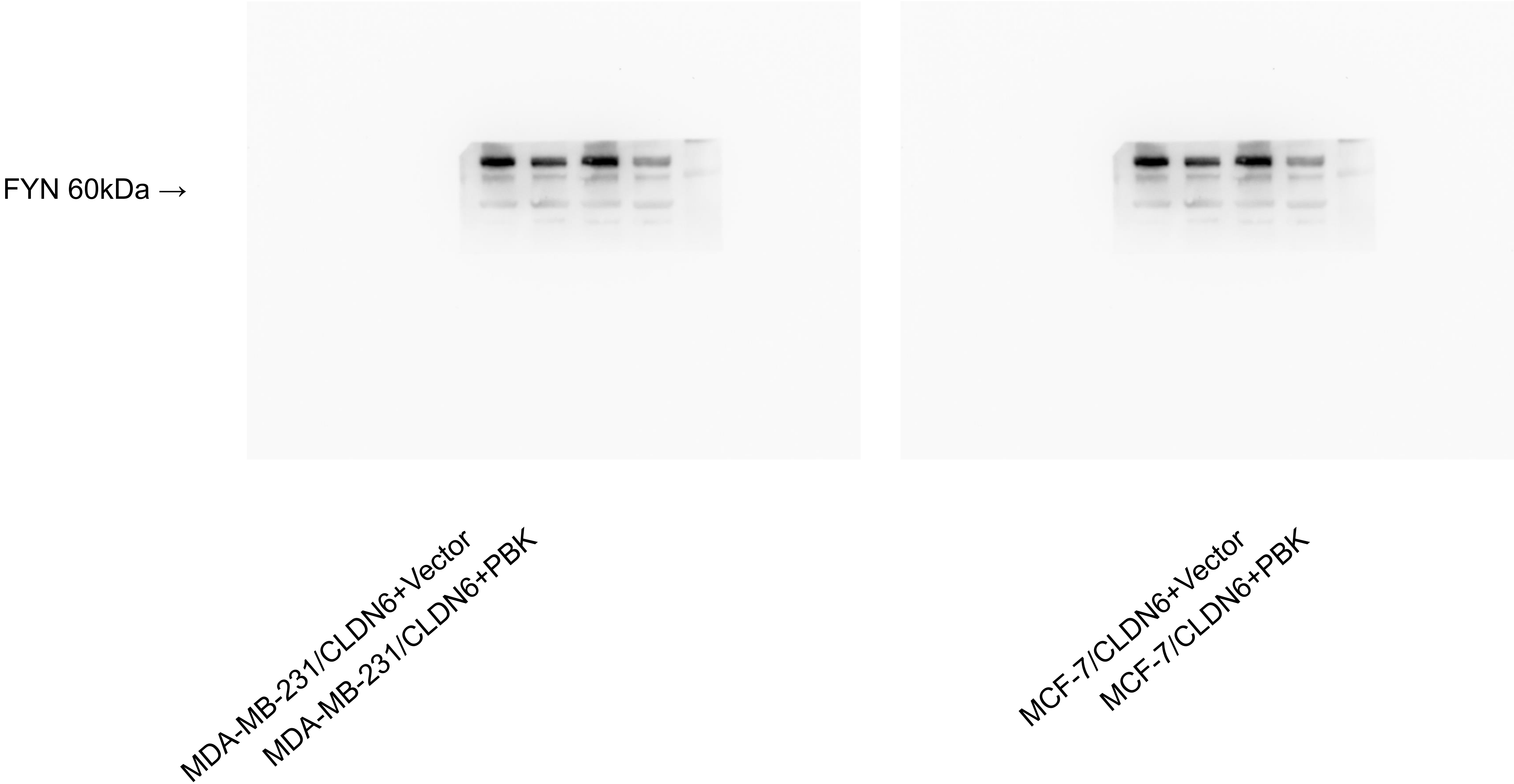
FYN 60kDa →
MDA-MB-231/CLDN6+Vector
MDA-MB-231/CLDN6+PBK
MCF-7/CLDN6+Vector
MCF-7/CLDN6+PBK

Fig. 4 F

H3 17kDa →

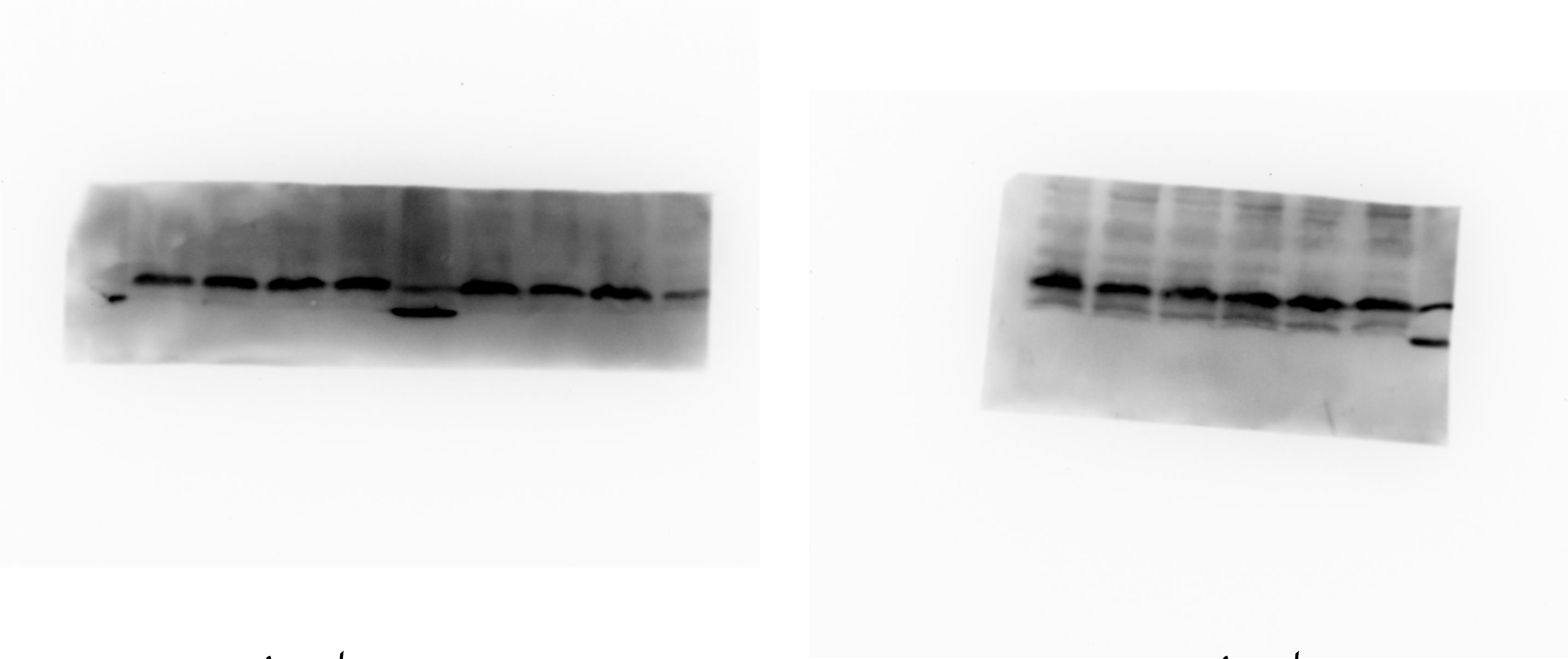

MDA-MB-231/CLDN6+Vector
MDA-MB-231/CLDN6+PBK

MCF-7/CLDN6+Vector
MCF-7/CLDN6+PBK

Fig. 4 F

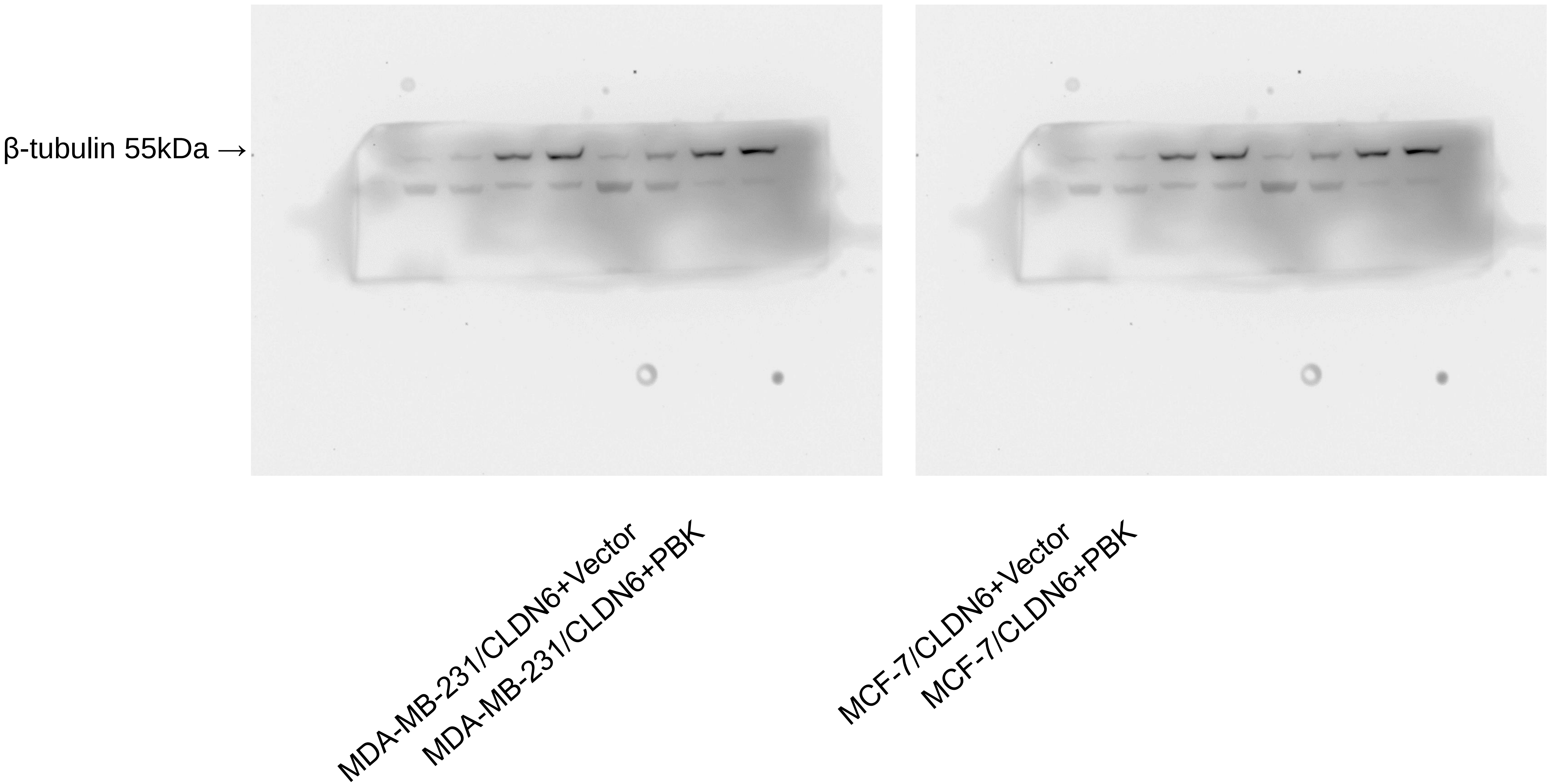
β-tubulin 55kDa →
MDA-MB-231/CLDN6+Vector
MDA-MB-231/CLDN6+PBK
MCF-7/CLDN6+Vector
MCF-7/CLDN6+PBK

Fig. 4 G

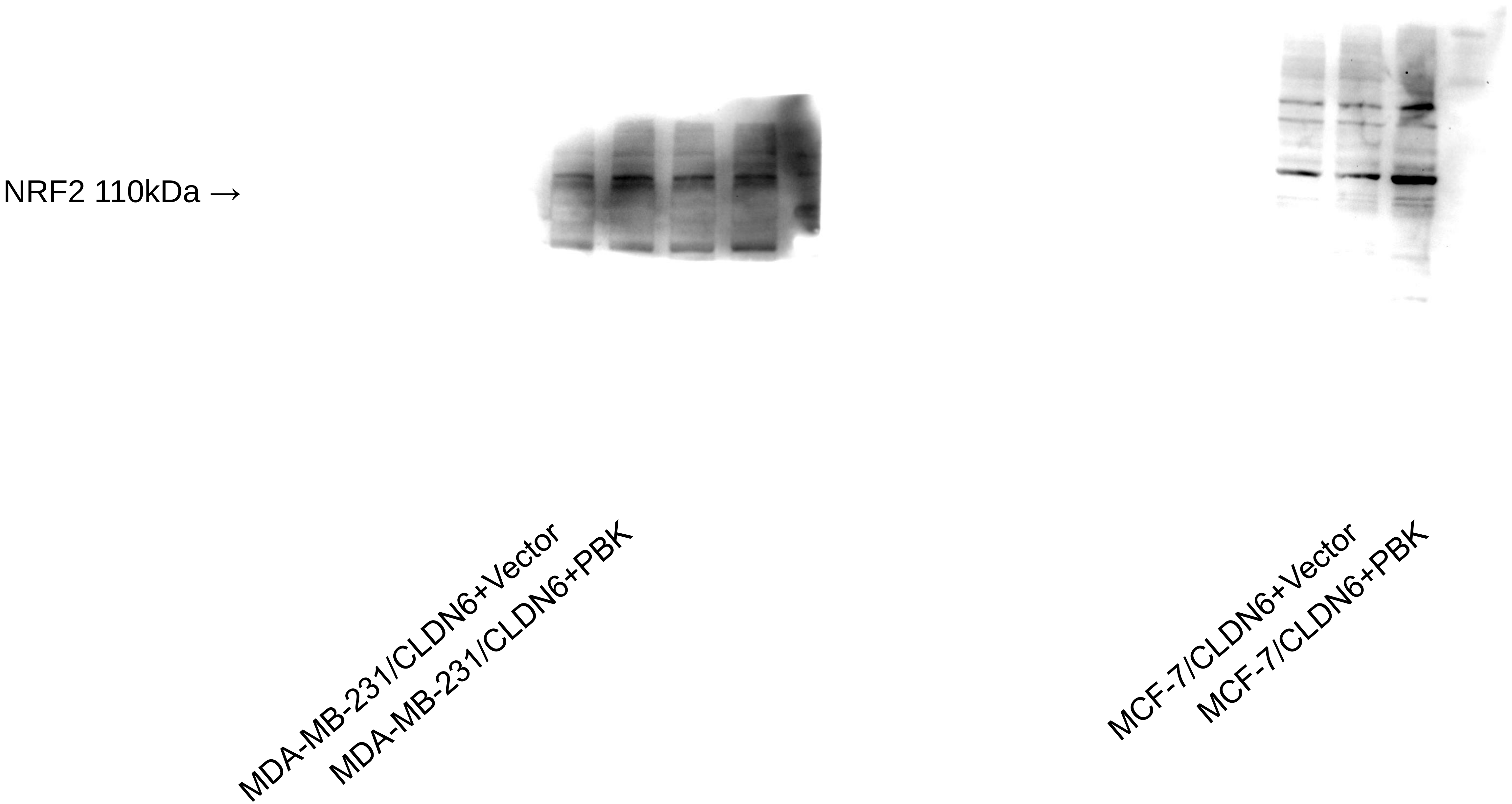
NRF2 110kDa →
MDA-MB-231/CLDN6+Vector
MDA-MB-231/CLDN6+PBK
MCF-7/CLDN6+Vector
MCF-7/CLDN6+PBK

Fig. 4 G

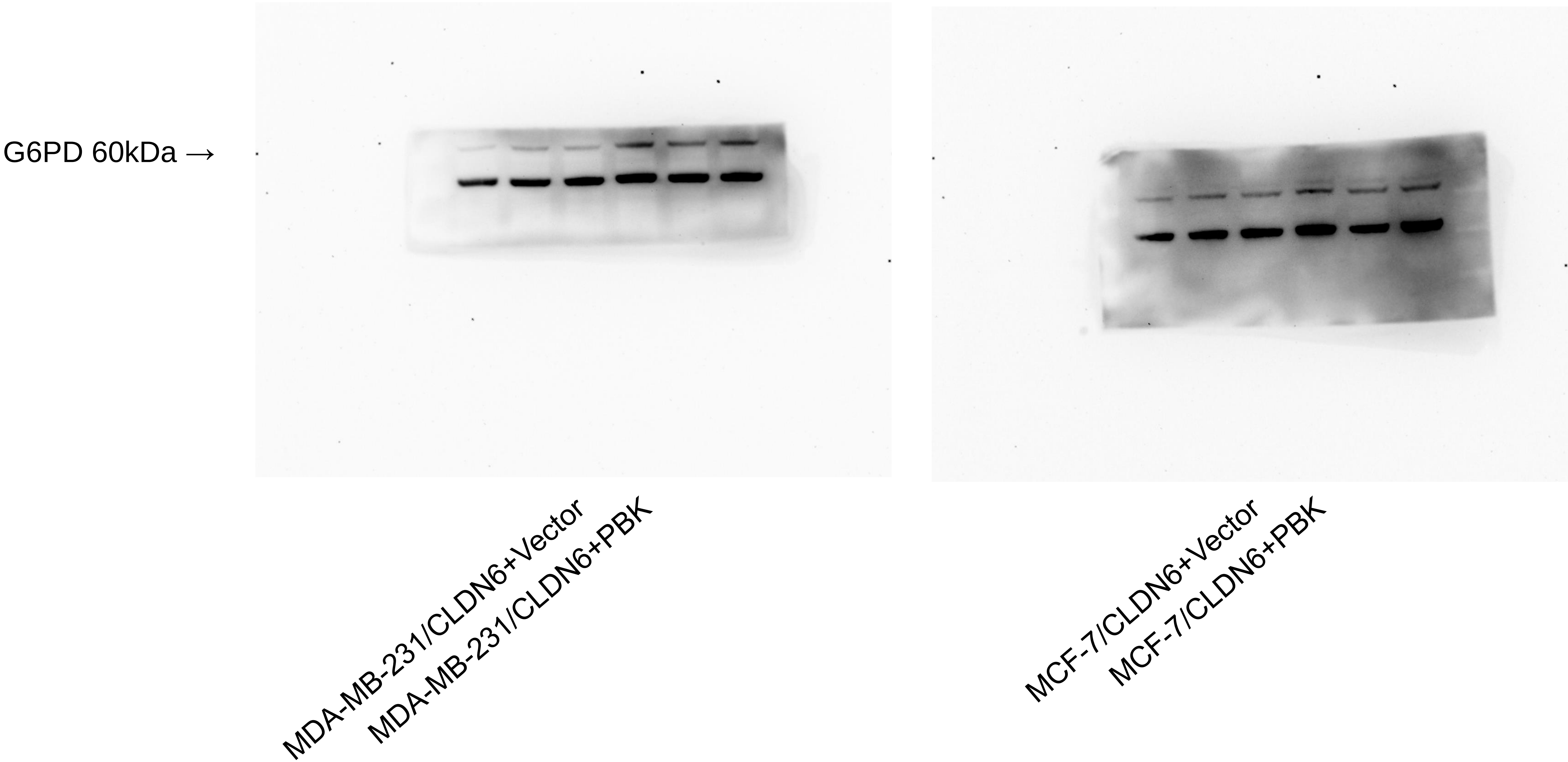

G6PD 60kDa →
MDA-MB-231/CLDN6+Vector
MDA-MB-231/CLDN6+PBK
MCF-7/CLDN6+Vector
MCF-7/CLDN6+PBK

Fig. 4 G

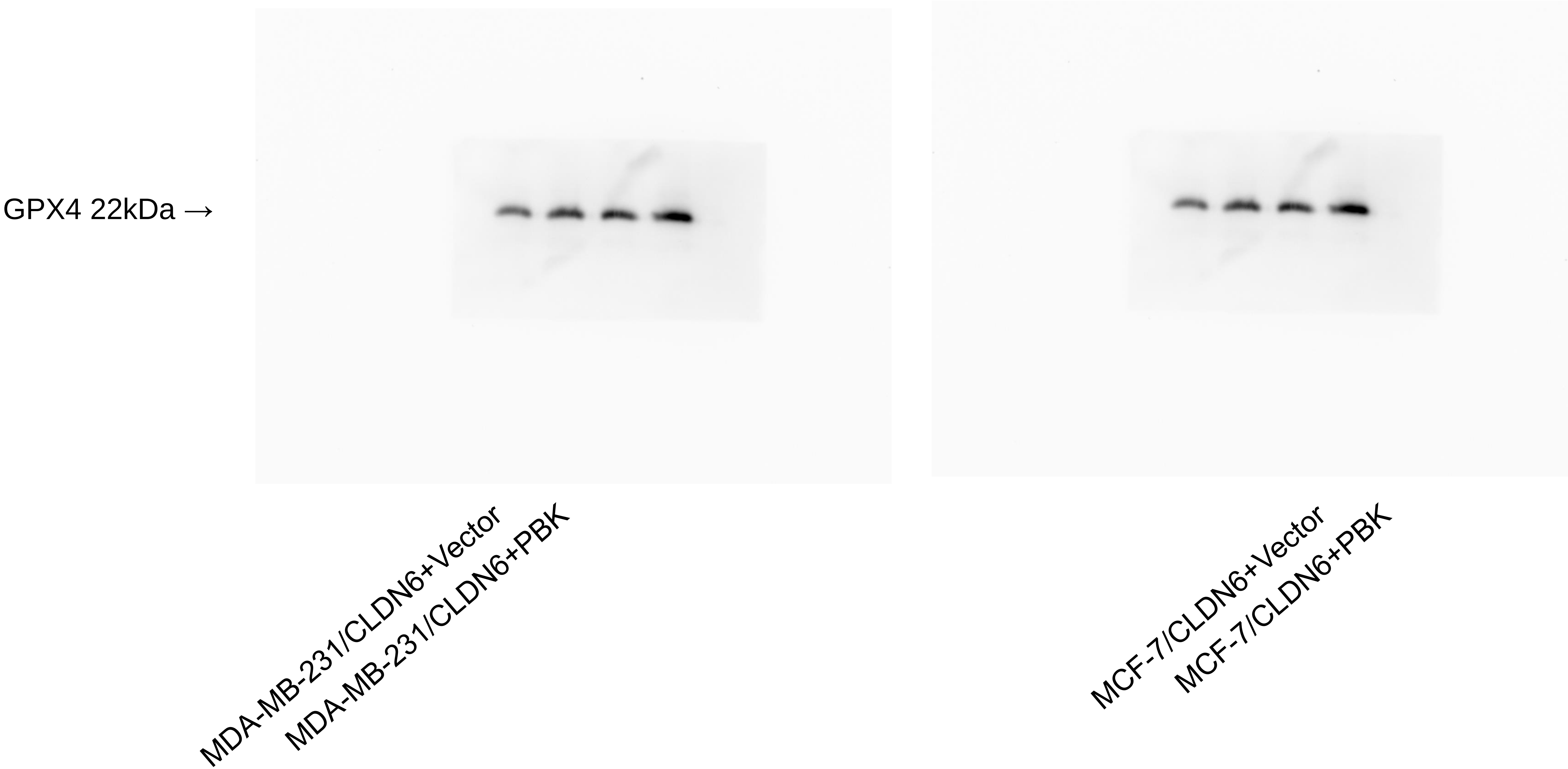
GPX4 22kDa →
MDA-MB-231/CLDN6+Vector
MDA-MB-231/CLDN6+PBK
MCF-7/CLDN6+Vector
MCF-7/CLDN6+PBK

Fig. 4 G

β-actin 42kDa →

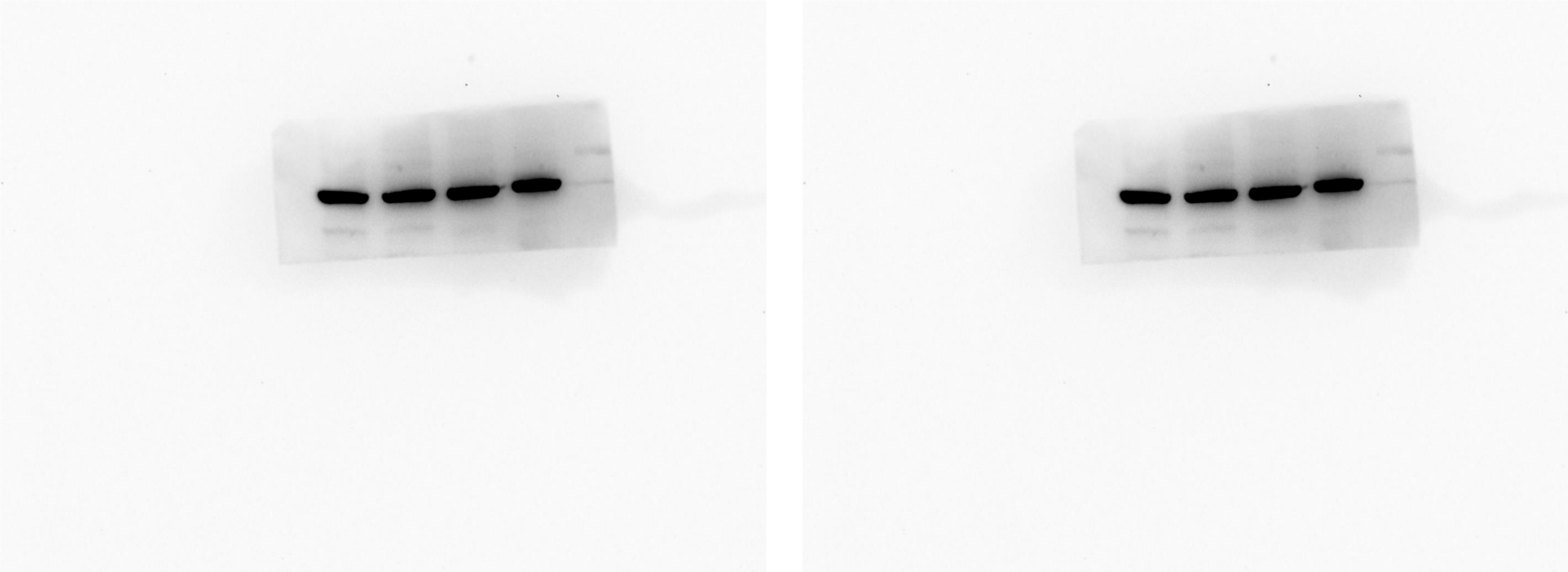

MDA-MB-231/CLDN6+Vector
MDA-MB-231/CLDN6+PBK

MCF-7/CLDN6+Vector
MCF-7/CLDN6+PBK

Fig. 4 H

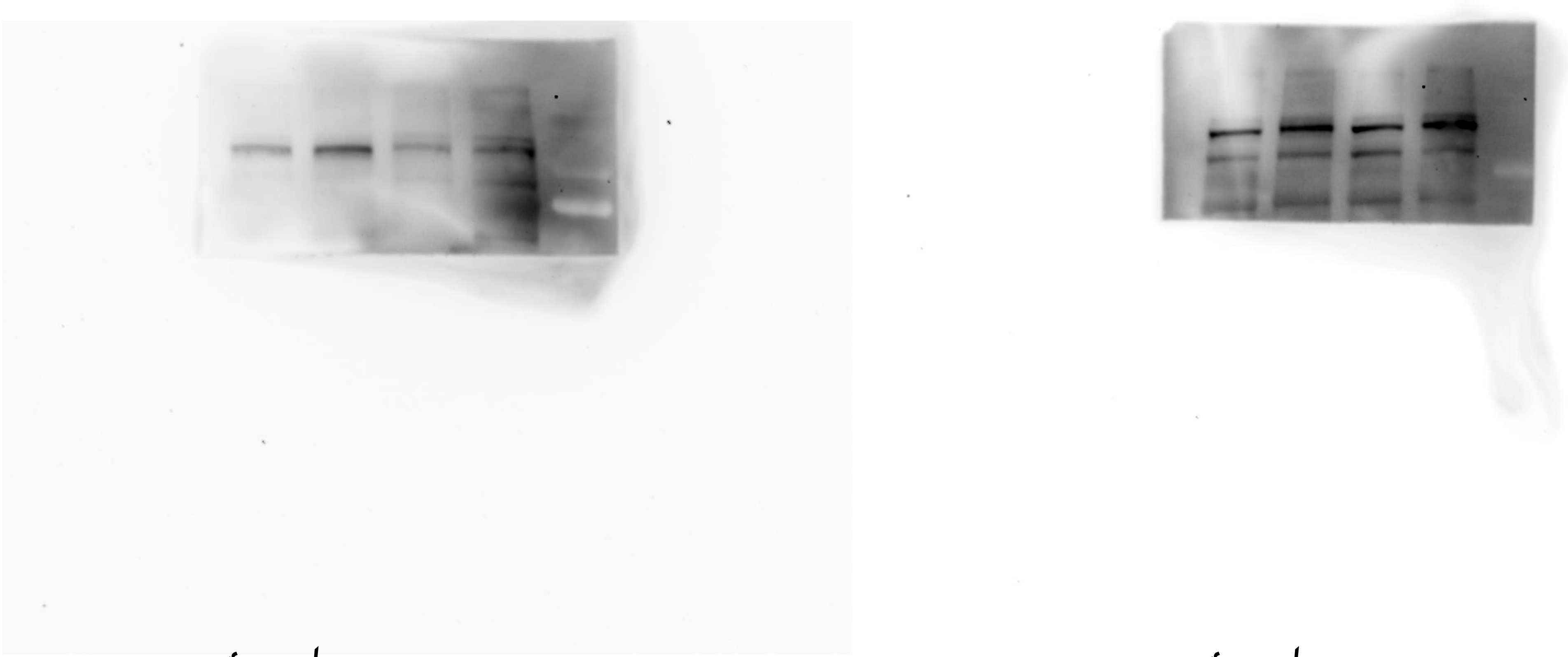
NRF2 110kDa →
MDA-MB-231/CLDN6+Vector
MDA-MB-231/CLDN6+PBK
MCF-7/CLDN6+Vector
MCF-7/CLDN6+PBK

Fig. 4 H

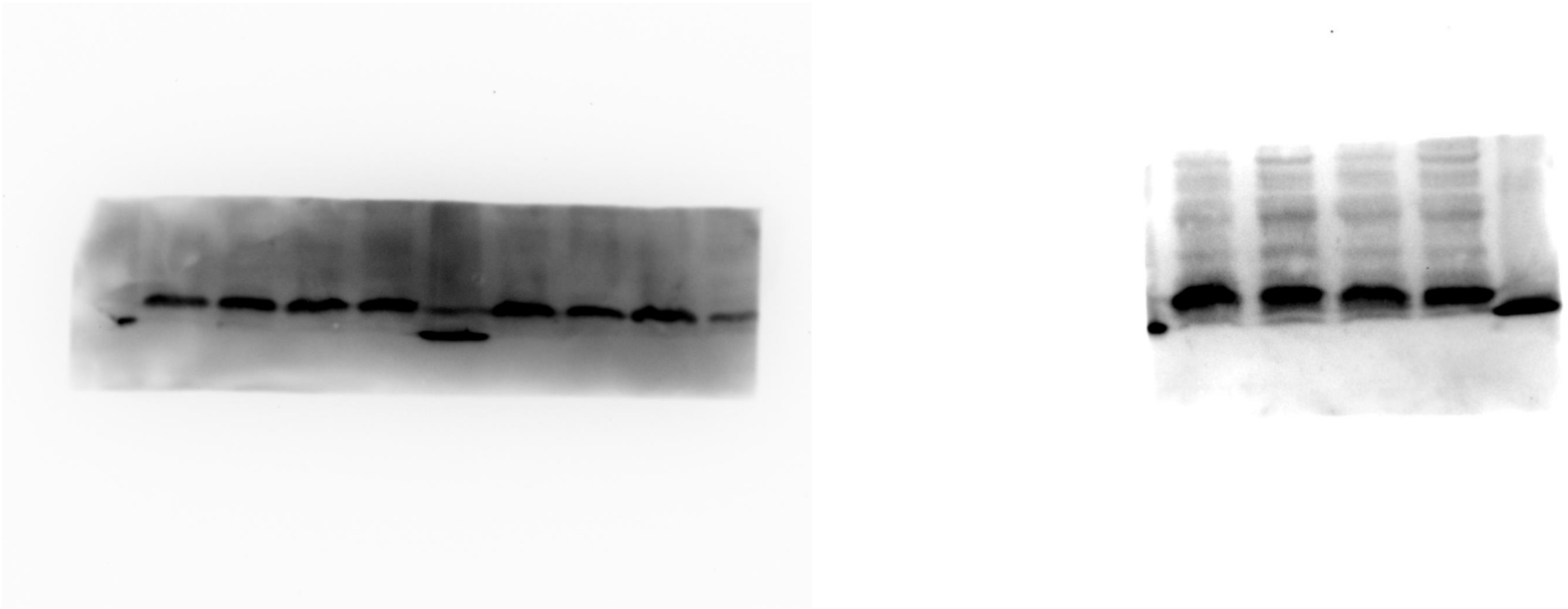
H3 17kDa →
MDA-MB-231/CLDN6+Vector
MDA-MB-231/CLDN6+PBK
MCF-7/CLDN6+Vector
MCF-7/CLDN6+PBK

Fig. 4 H

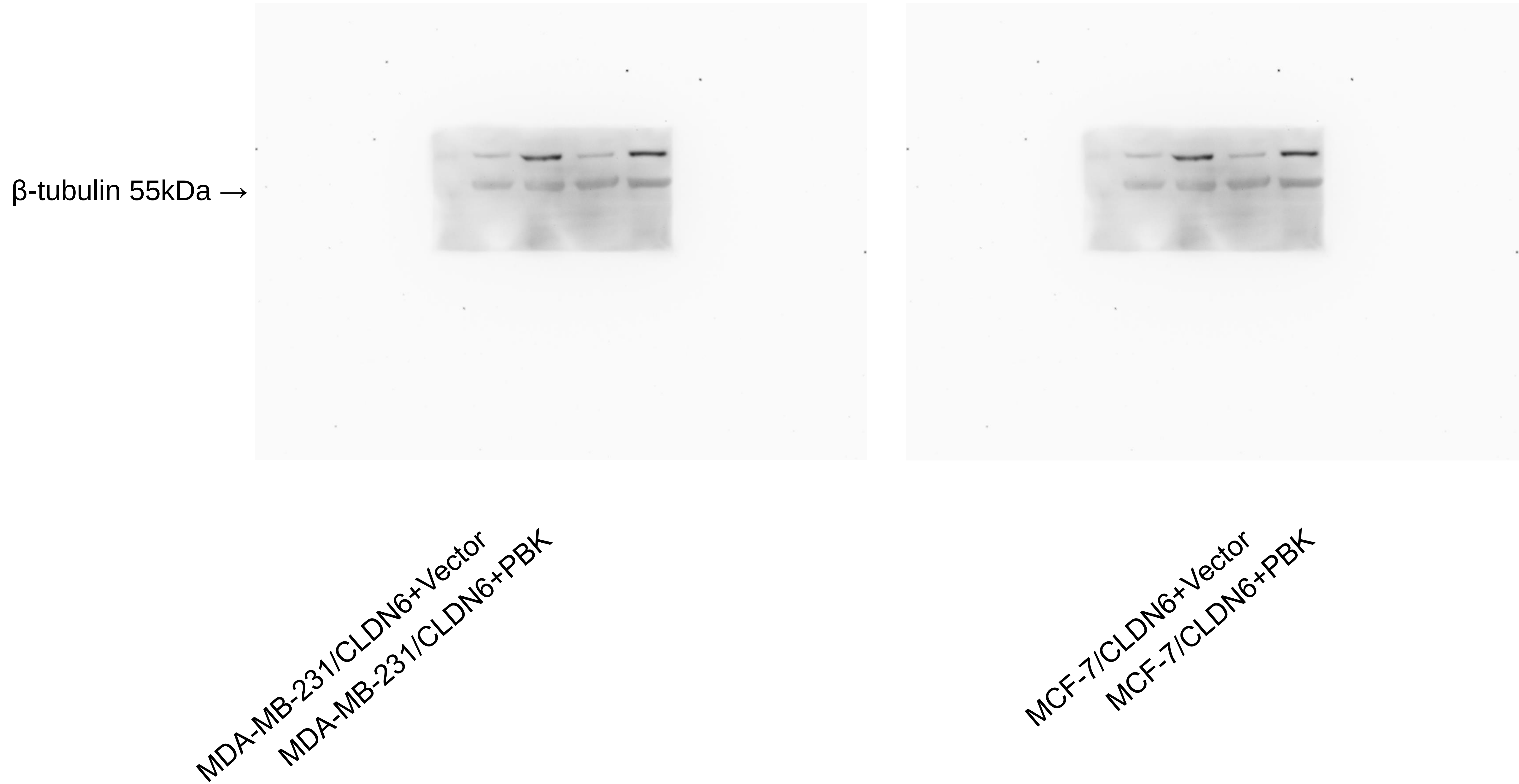
β-tubulin 55kDa →
MDA-MB-231/CLDN6+Vector
MDA-MB-231/CLDN6+PBK
MCF-7/CLDN6+Vector
MCF-7/CLDN6+PBK

Fig. 4 J

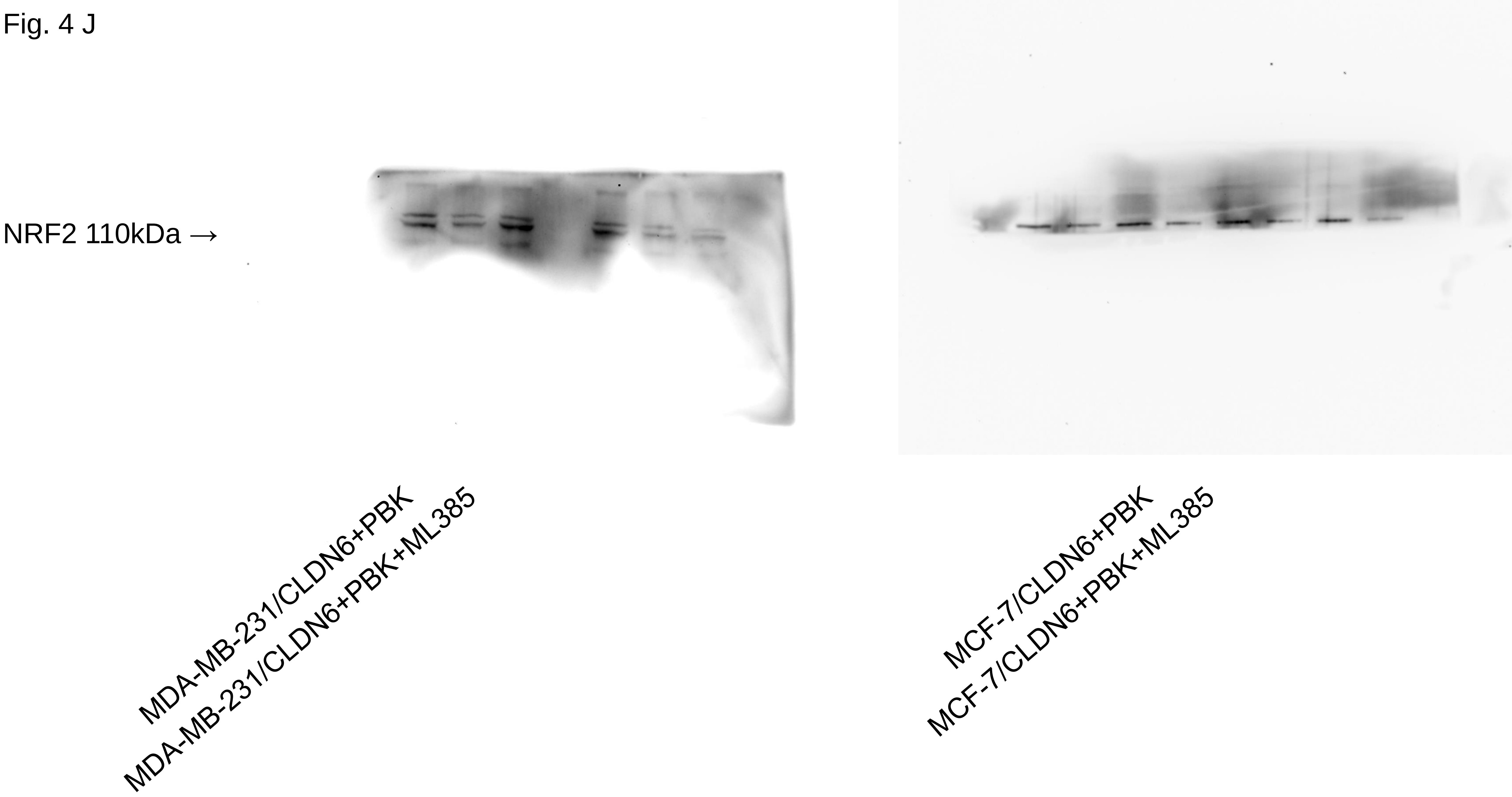
NRF2 110kDa →
MDA-MB-231/CLDN6+PBK
MDA-MB-231/CLDN6+PBK+ML385
MCF-7/CLDN6+PBK
MCF-7/CLDN6+PBK+ML385

Fig. 4 J

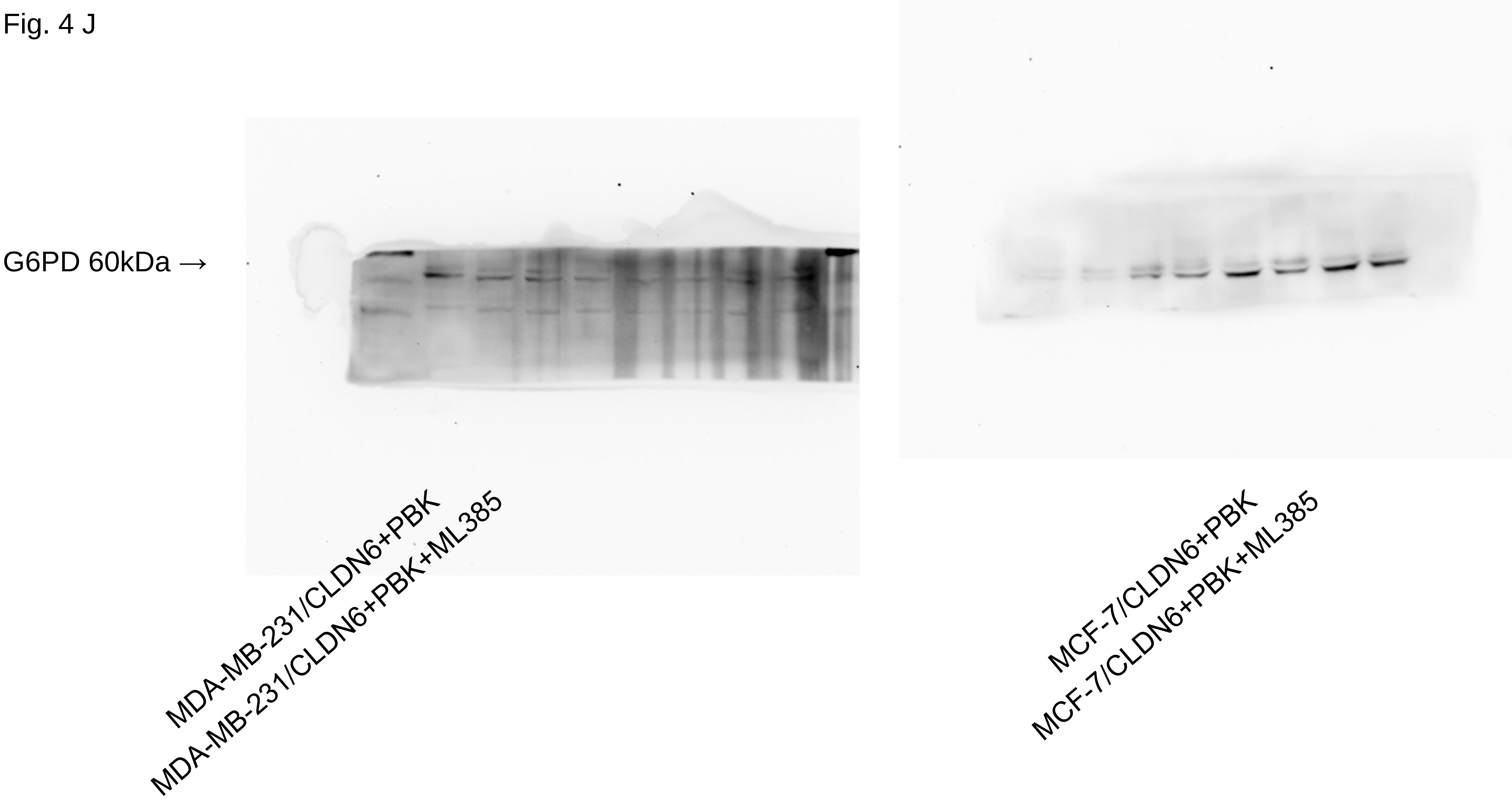
G6PD 60kDa →
MDA-MB-231/CLDN6+PBK
MDA-MB-231/CLDN6+PBK+ML385
MCF-7/CLDN6+PBK
MCF-7/CLDN6+PBK+ML385

Fig. 4 J

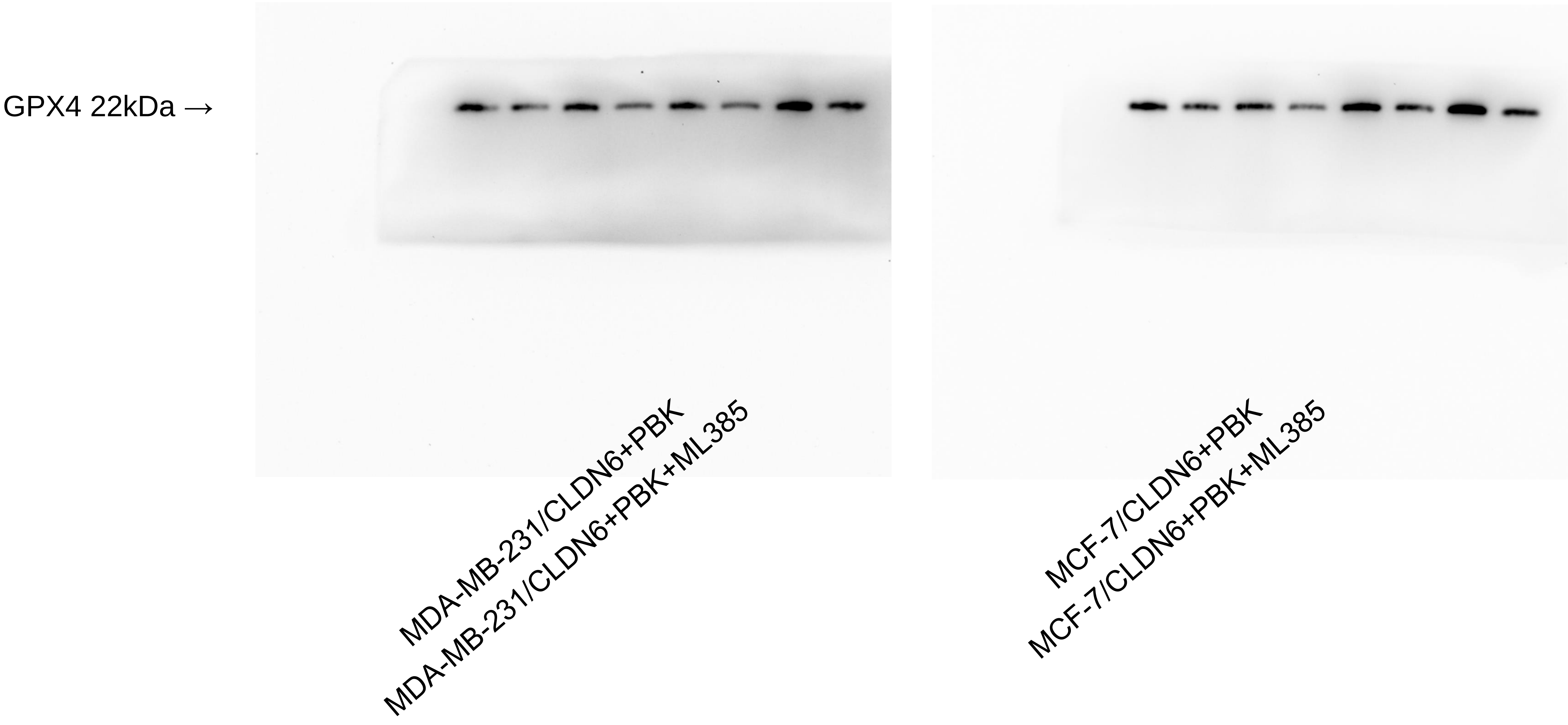
GPX4 22kDa →
MDA-MB-231/CLDN6+PBK
MDA-MB-231/CLDN6+PBK+ML385
MCF-7/CLDN6+PBK
MCF-7/CLDN6+PBK+ML385

Fig. 4 J

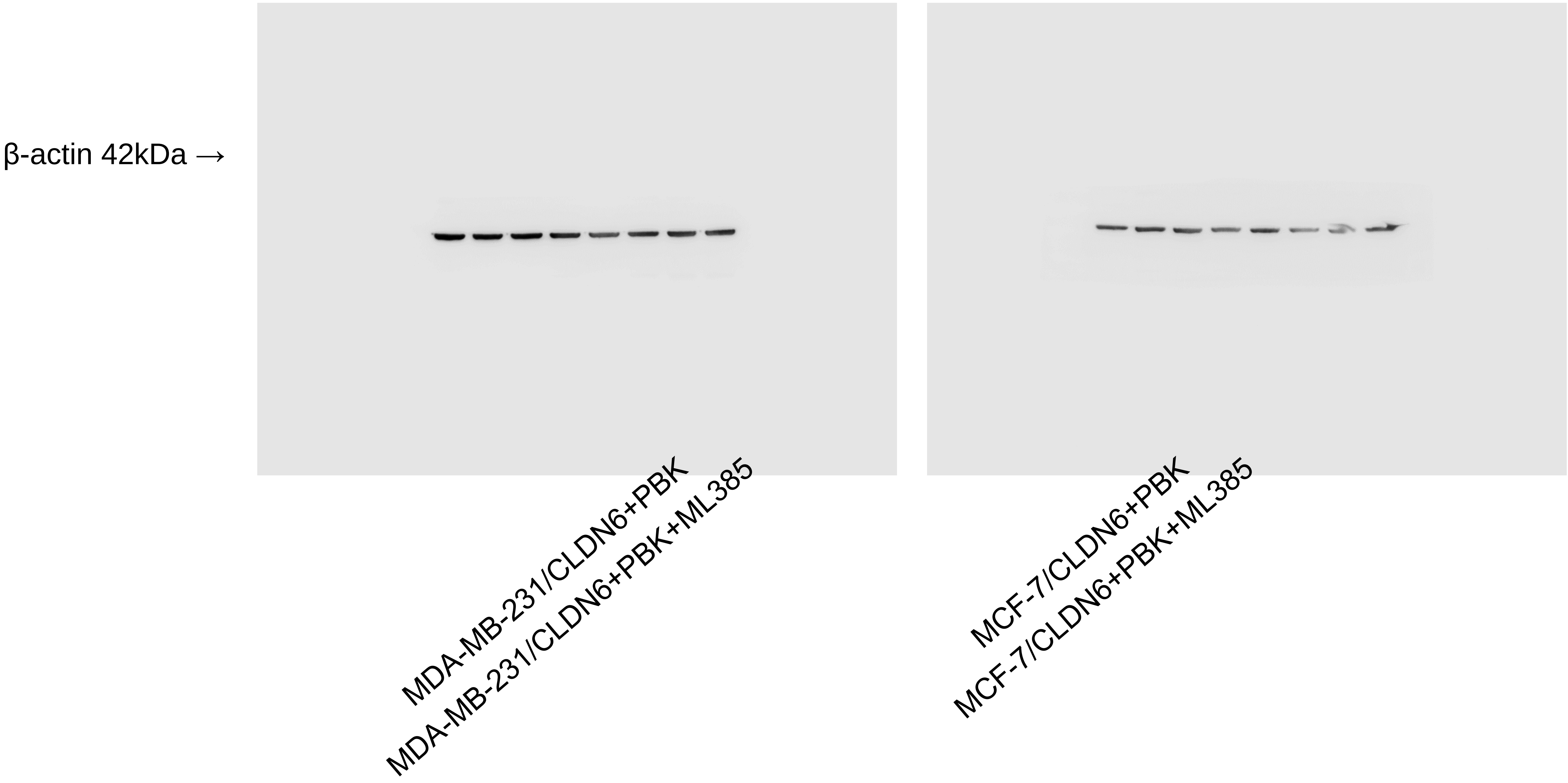
β-actin 42kDa →
MDA-MB-231/CLDN6+PBK
MDA-MB-231/CLDN6+PBK+ML385
MCF-7/CLDN6+PBK
MCF-7/CLDN6+PBK+ML385

Fig. 5 C

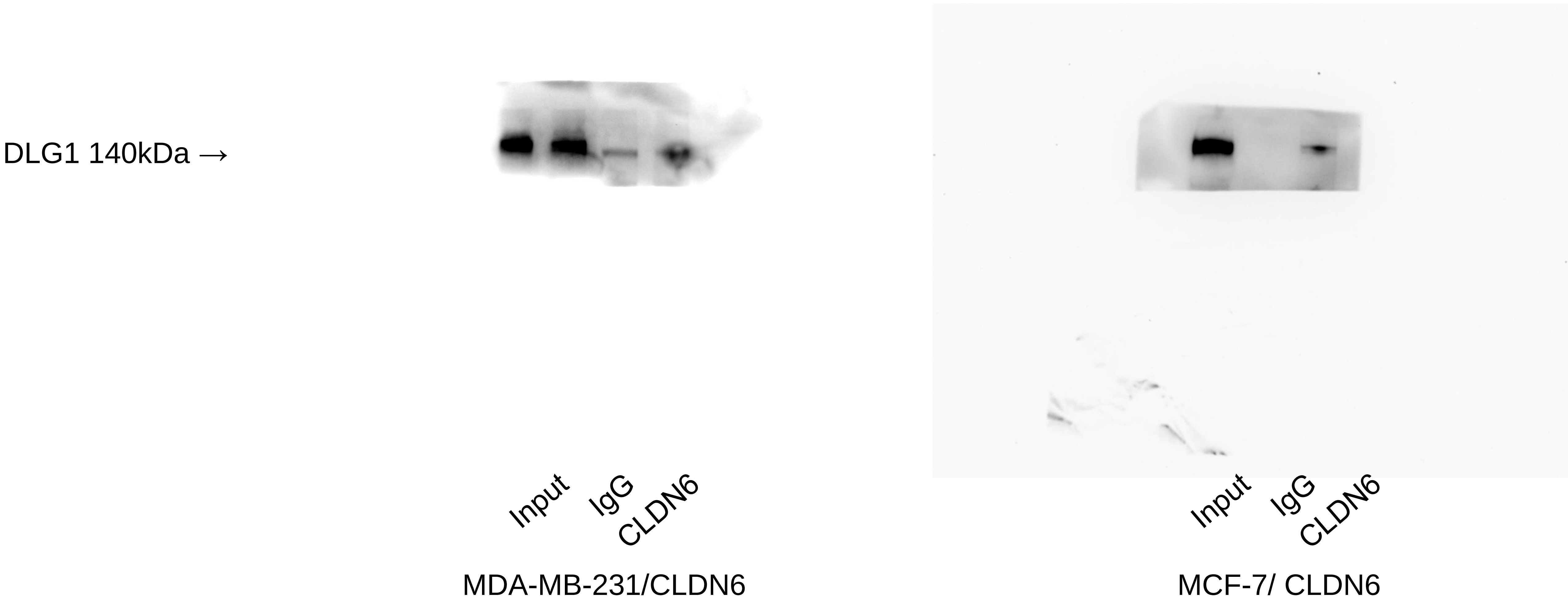
DLG1 140kDa →
Input
IgG
CLDN6
MDA-MB-231/CLDN6
Input
IgG
CLDN6
MCF-7/ CLDN6

Fig. 5 C

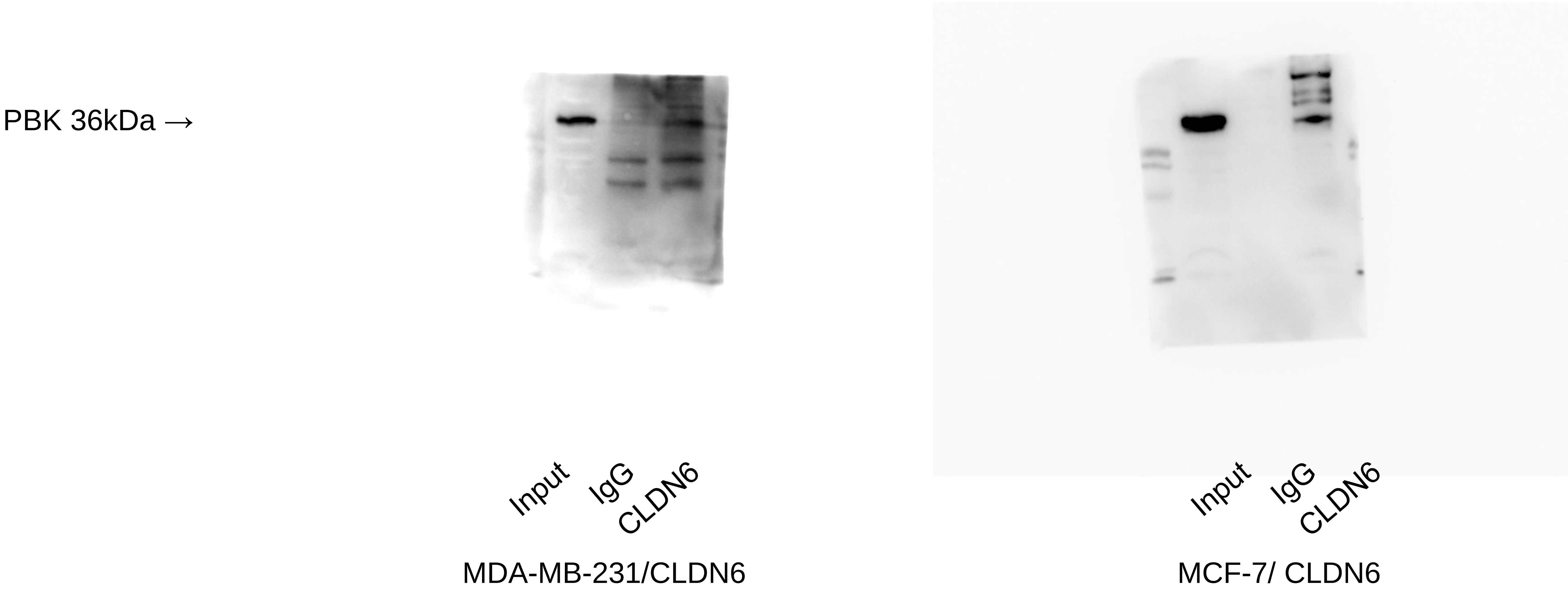

PBK 36kDa →
Input
IgG
CLDN6
MDA-MB-231/CLDN6
Input
IgG
CLDN6
MCF-7/ CLDN6

Fig. 5 G

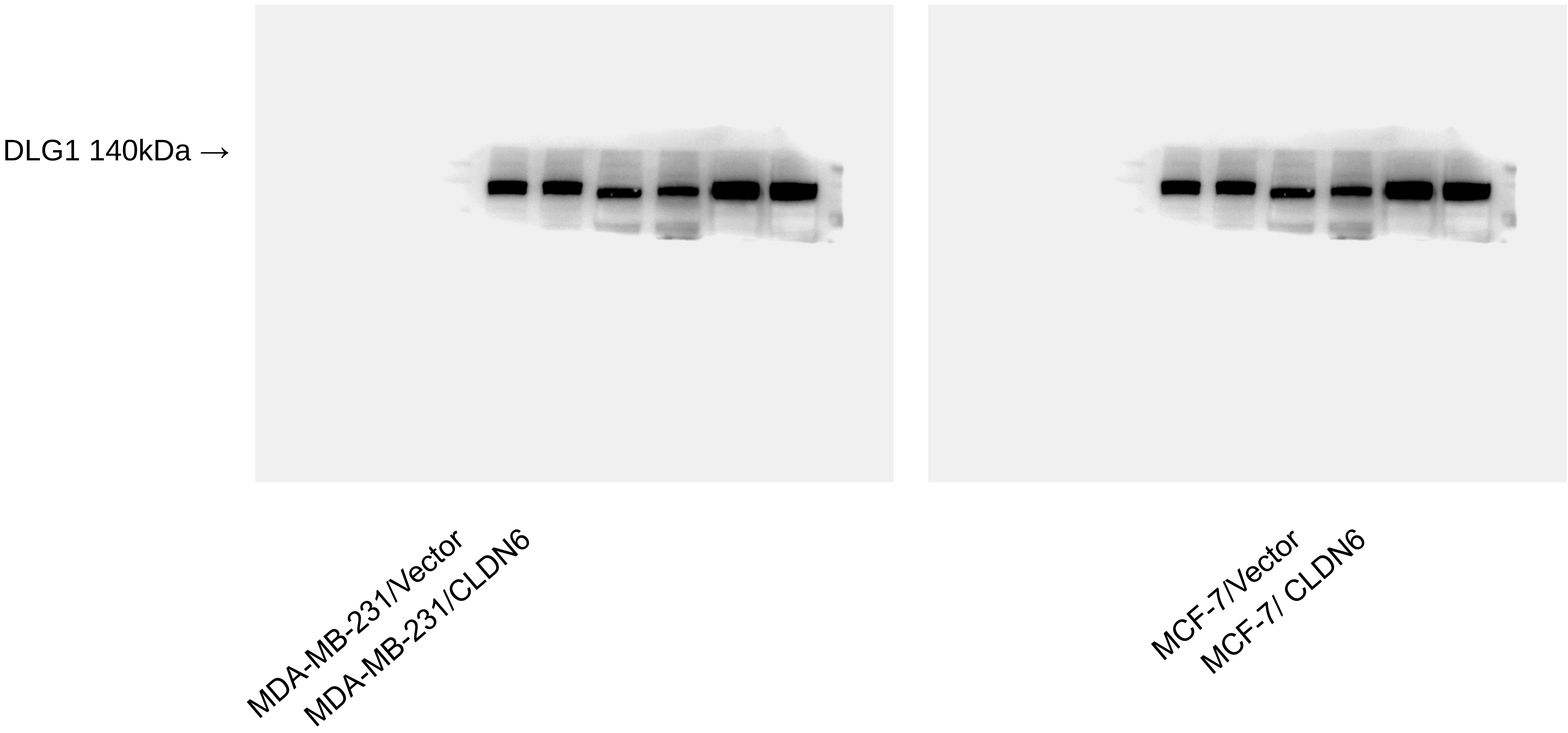
DLG1 140kDa →
MDA-MB-231/Vector
MDA-MB-231/CLDN6
MCF-7/Vector
MCF-7/ CLDN6

Fig. 5 G

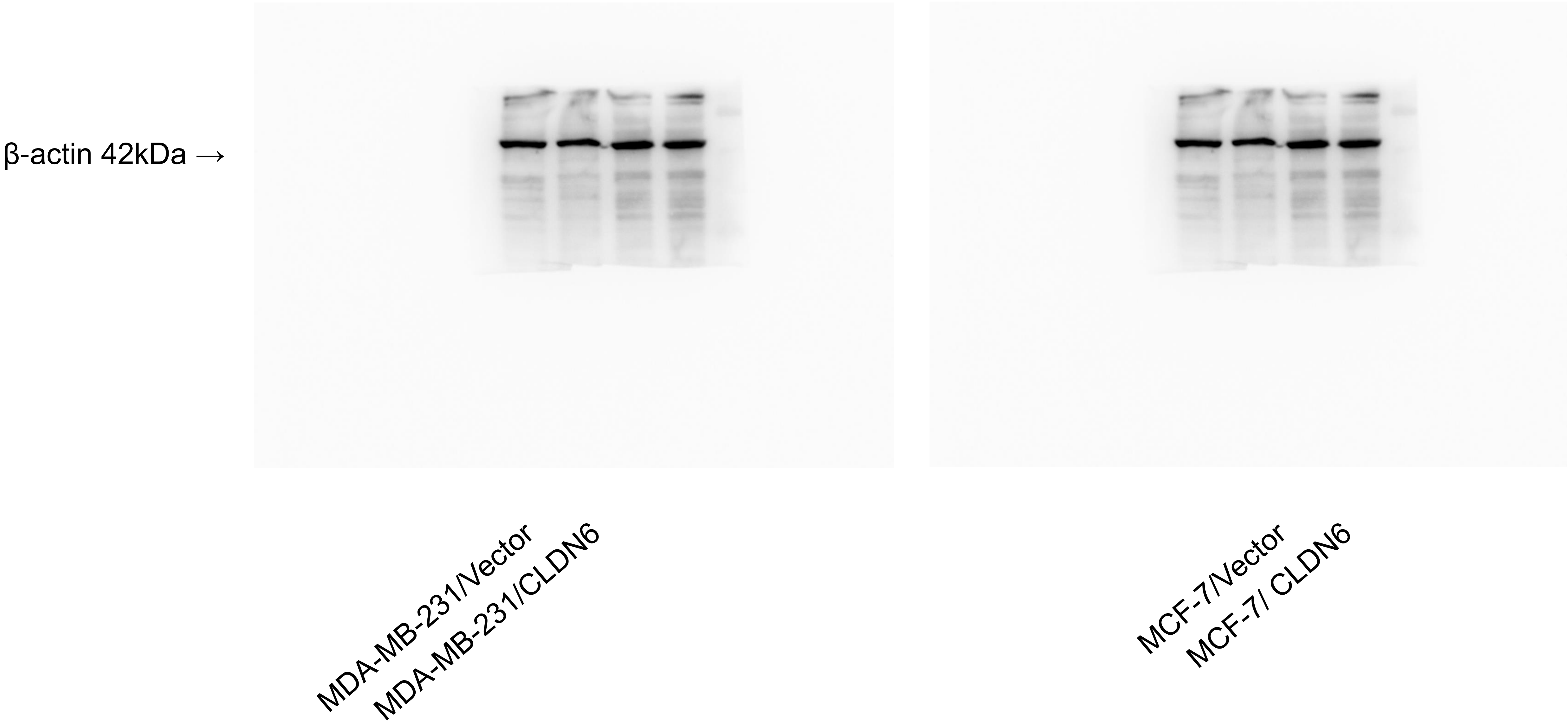

β-actin 42kDa →
MDA-MB-231/Vector
MDA-MB-231/CLDN6
MCF-7/Vector
MCF-7/ CLDN6

Fig. 5 l

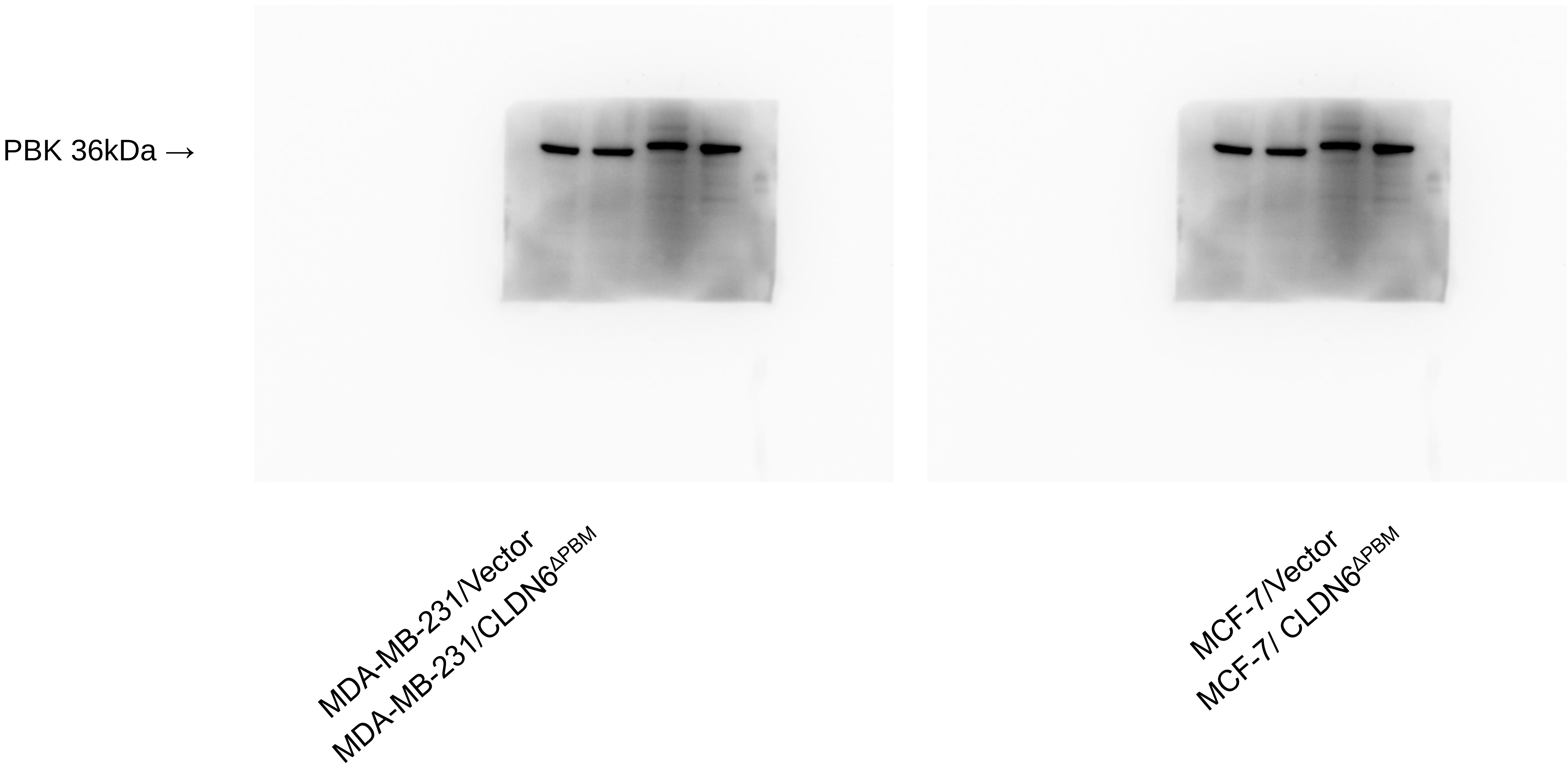
PBK 36kDa →
MDA-MB-231/Vector
MDA-MB-231/CLDN6ΔPBM
MCF-7/Vector
MCF-7/ CLDN6ΔPBM

Fig. 5 I

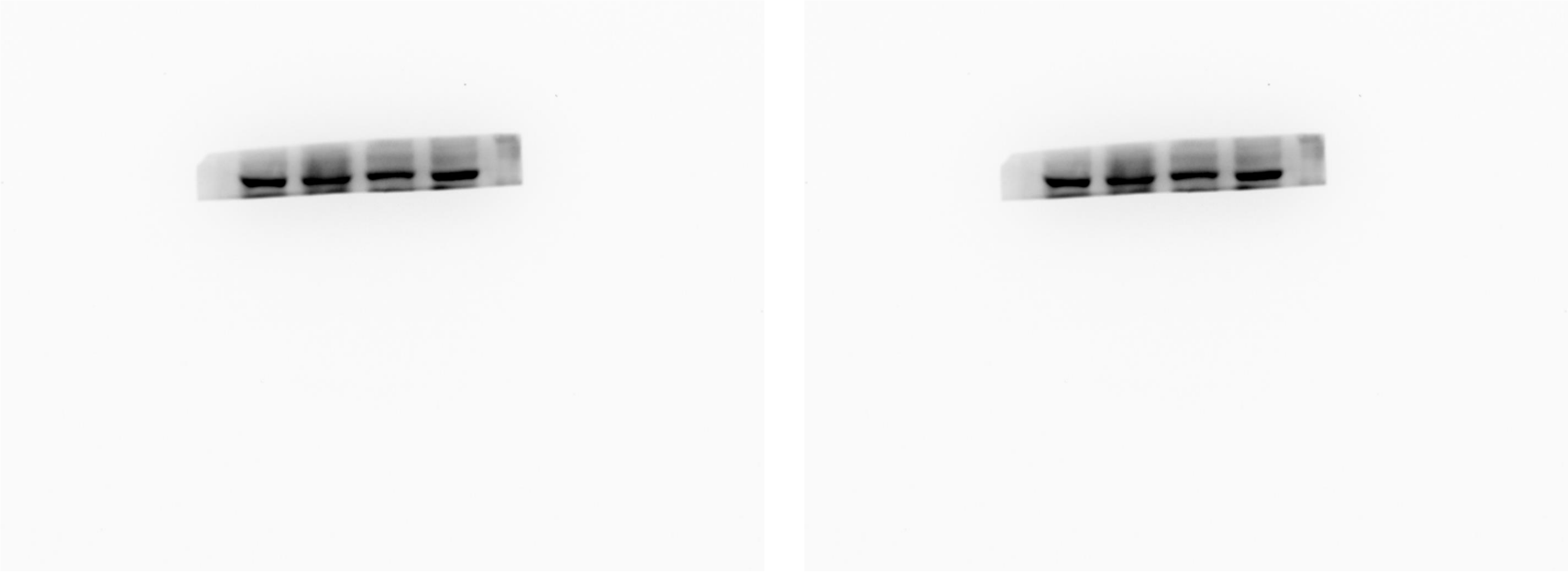
β-actin 42kDa →
MDA-MB-231/Vector
MDA-MB-231/CLDN6$^{\Delta PBM}$
MCF-7/Vector
MCF-7/ CLDN6$^{\Delta PBM}$

Fig. 5 J

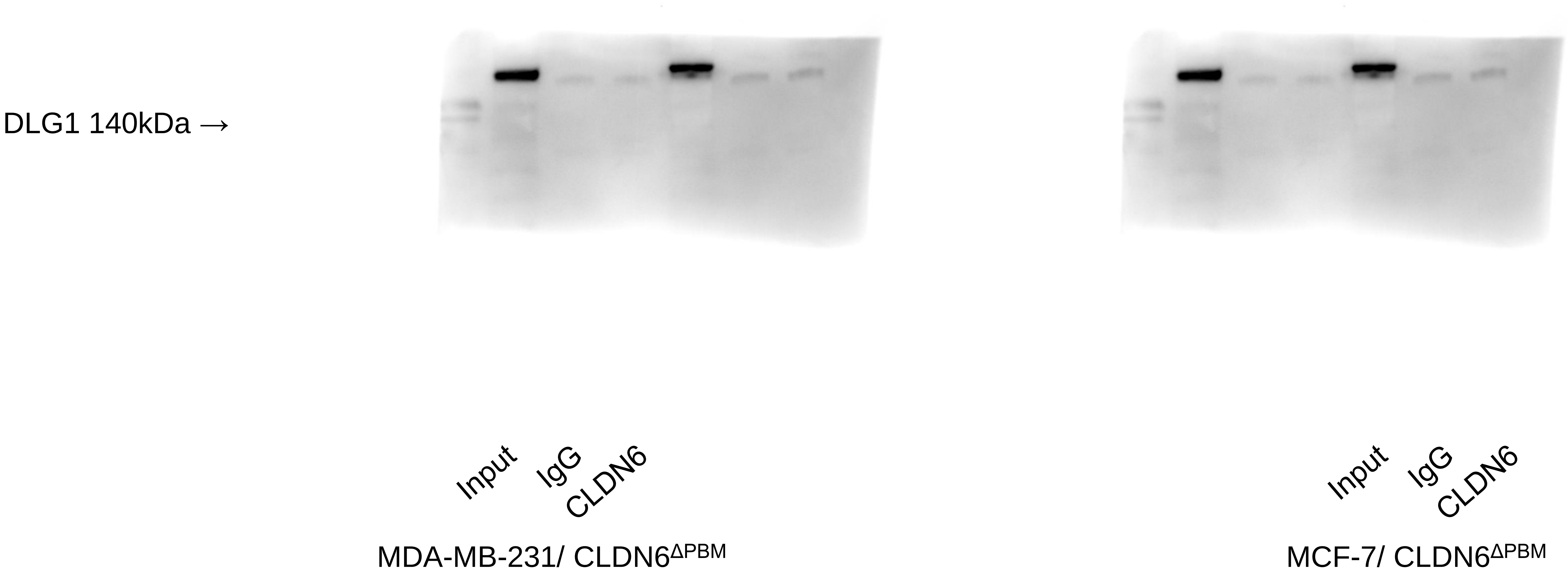
DLG1 140kDa →
Input
IgG
CLDN6
MDA-MB-231/ CLDN6$^{\Delta PBM}$
Input
IgG
CLDN6
MCF-7/ CLDN6$^{\Delta PBM}$

Fig. 5 J

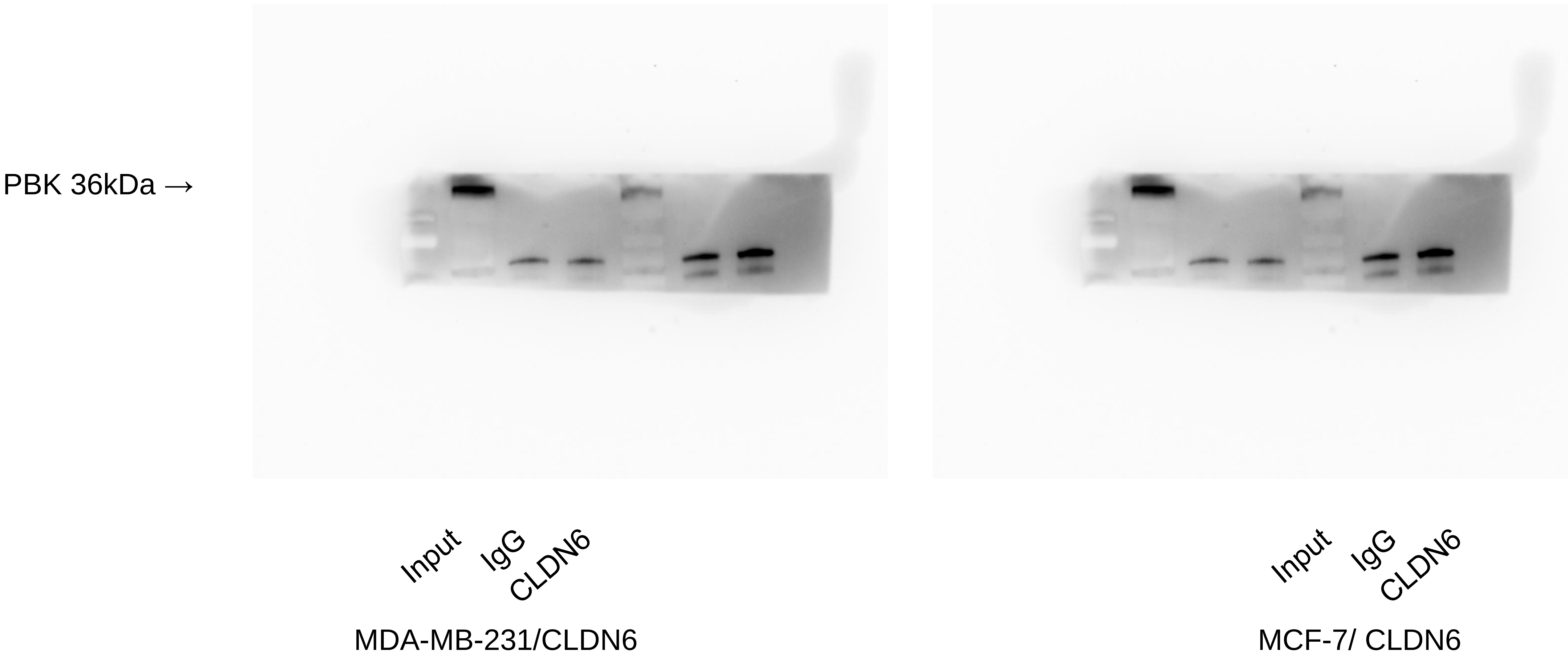
PBK 36kDa →
Input
IgG
CLDN6
MDA-MB-231/CLDN6
Input
IgG
CLDN6
MCF-7/ CLDN6

Fig. 6 E

NRF2 110kDa →

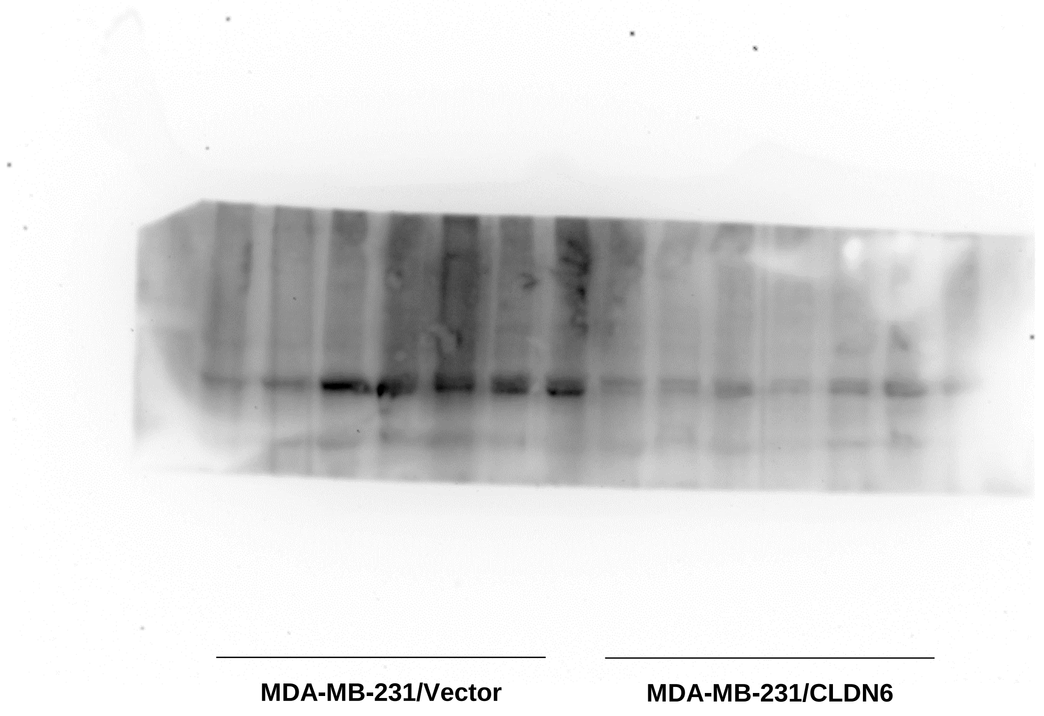

Fig. 6 E

GPX4 22kDa →

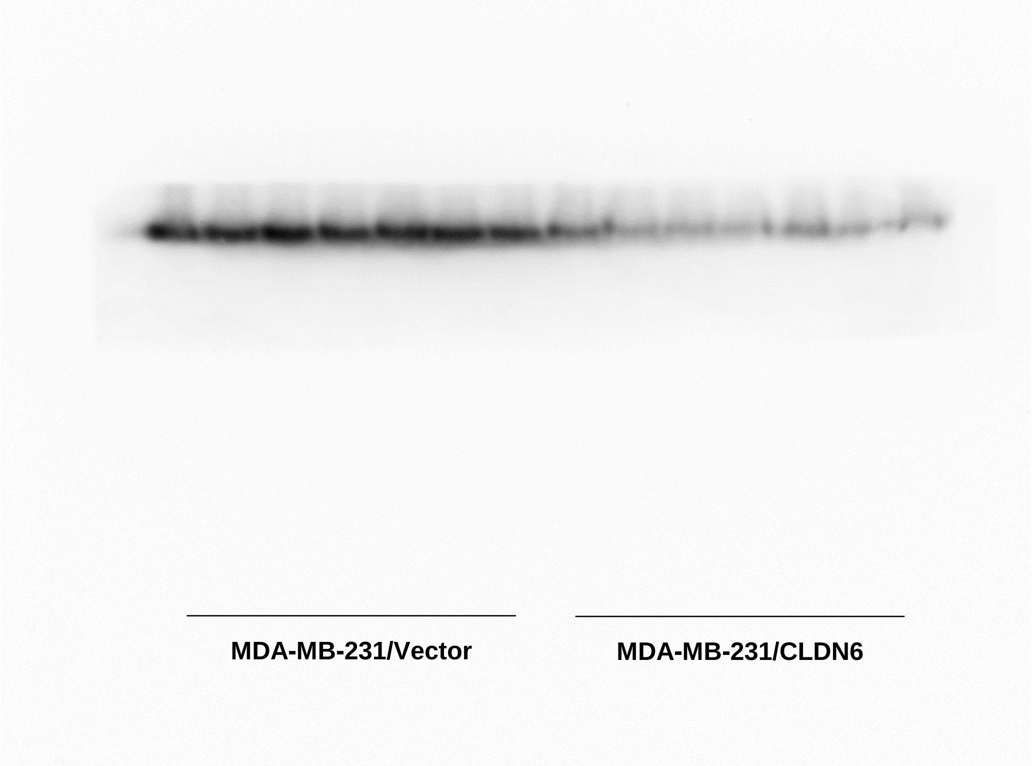

Fig. 6 E

CLDN6 23kDa →

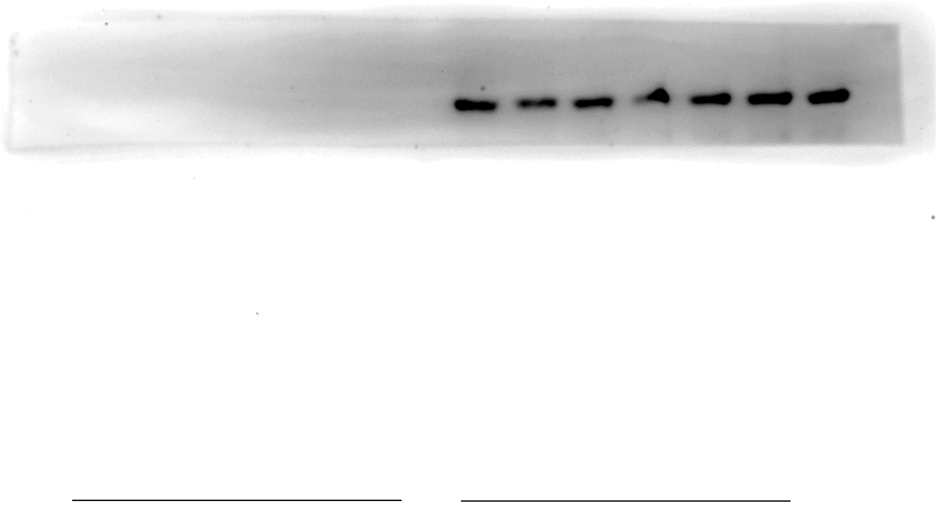

Fig. 6 E

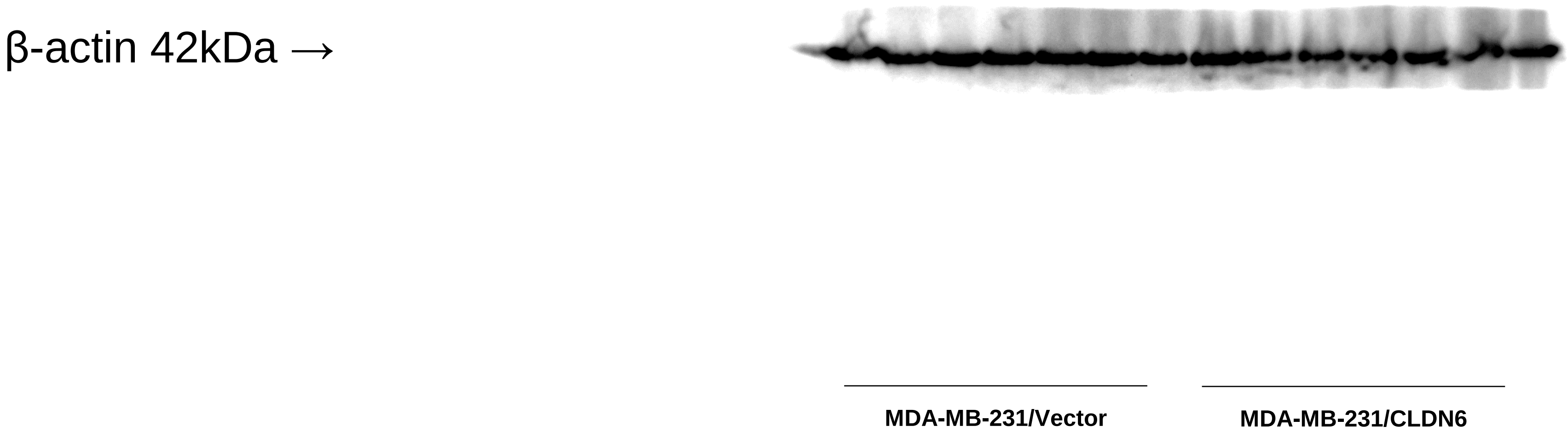

Fig. 6 E

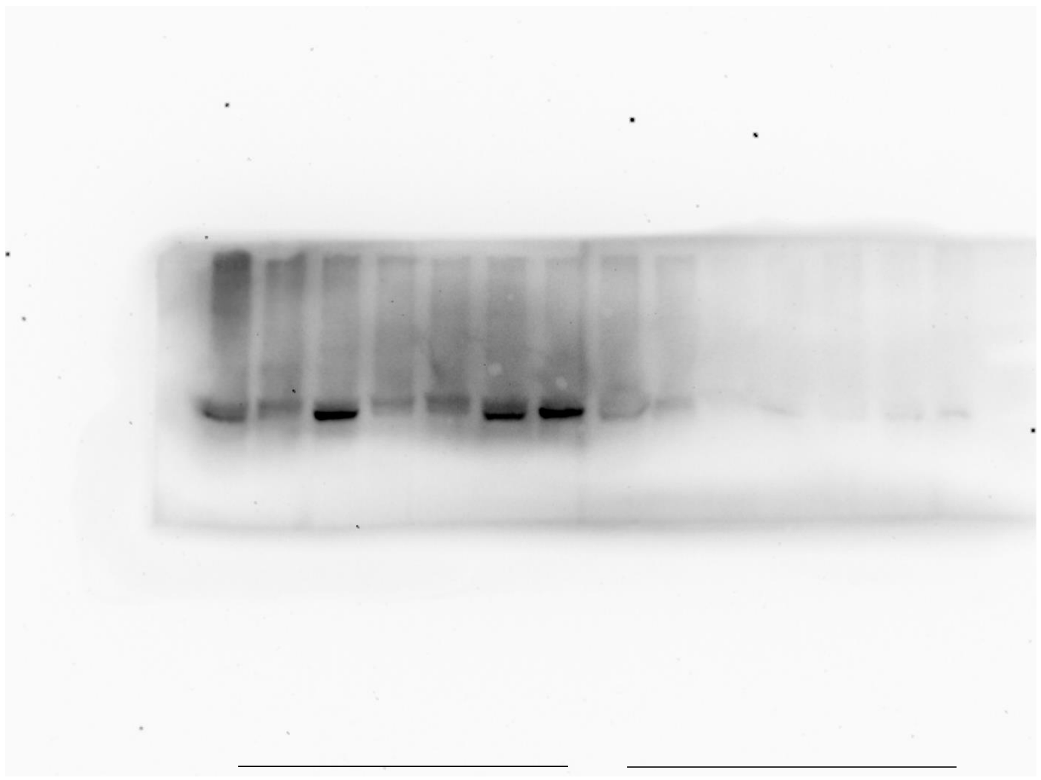

NRF2 110kDa →
MDA-MB-231/Vector+Sorafenib
MDA-MB-231/CLDN6+Sorafenib

Fig. 6 E

GPX4 22kDa →

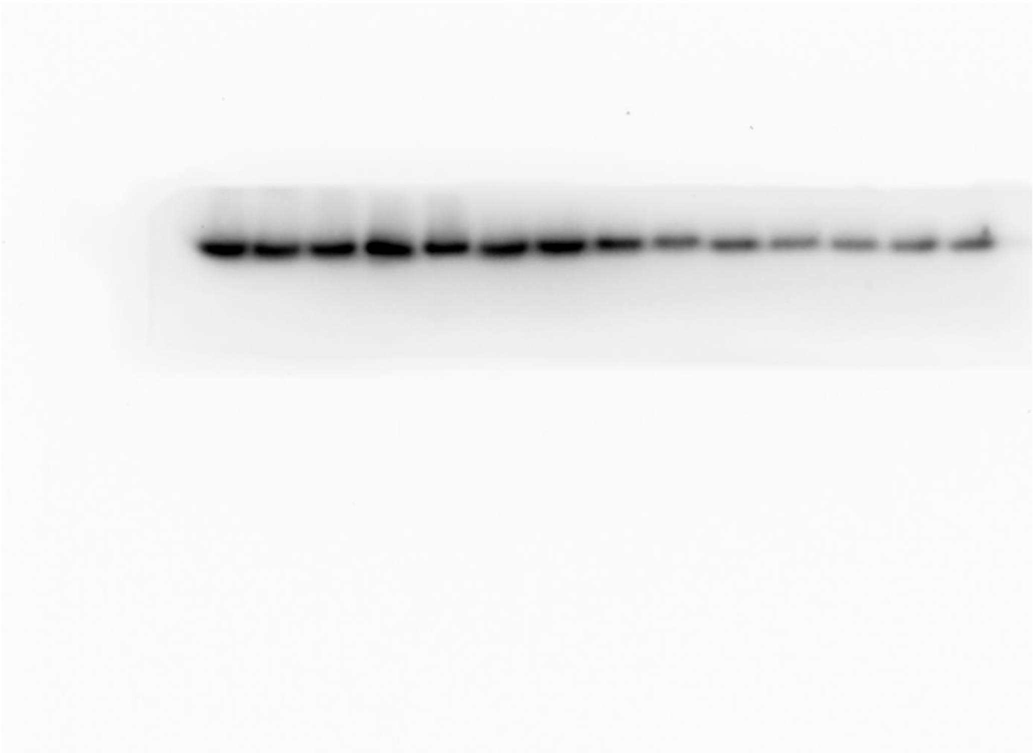

MDA-MB-231/Vector+Sorafenib

MDA-MB-231/CLDN6+Sorafenib

Fig. 6 E

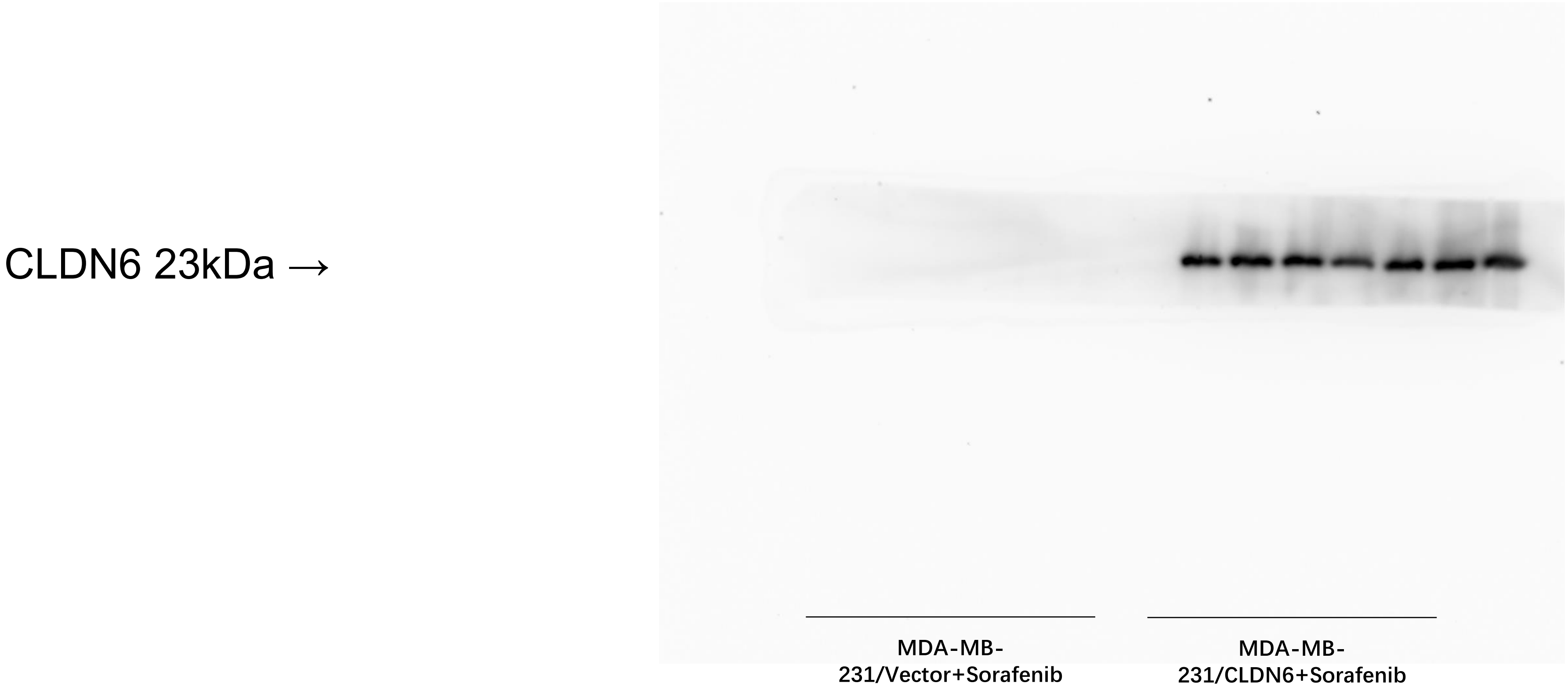
CLDN6 23kDa →
MDA-MB-231/Vector+Sorafenib
MDA-MB-231/CLDN6+Sorafenib

Fig. 6 E

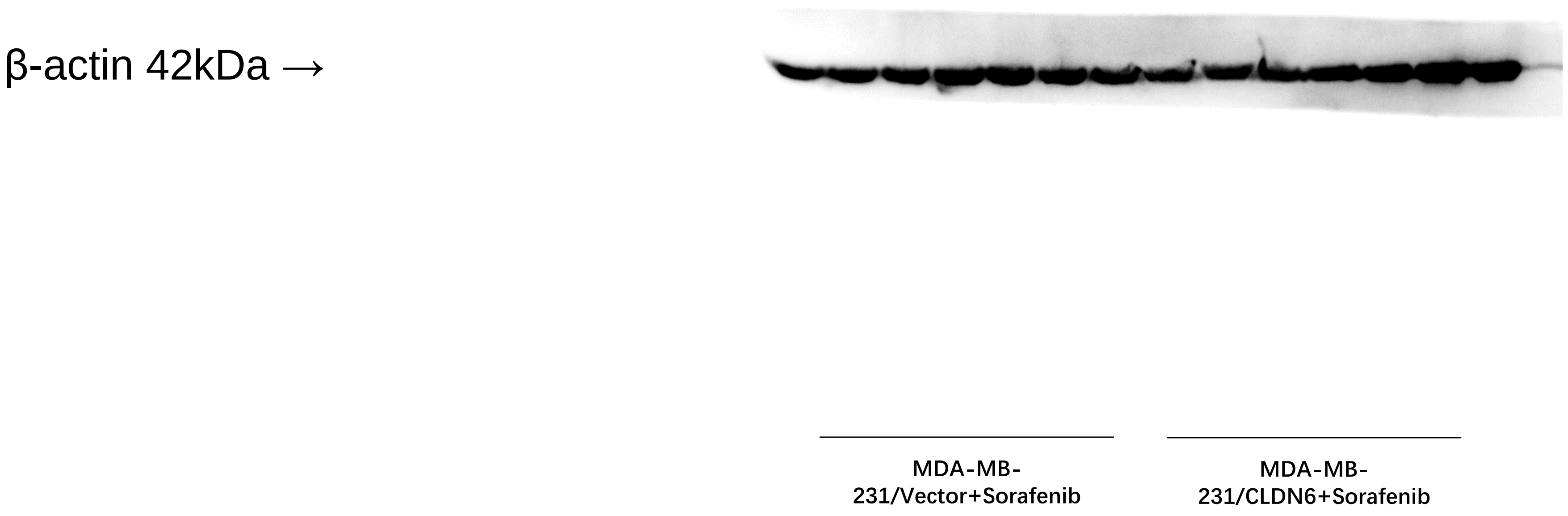
β-actin 42kDa →
MDA-MB-231/Vector+Sorafenib
MDA-MB-231/CLDN6+Sorafenib
